# Single nucleus multi-omics identifies human cortical cell regulatory genome diversity

**DOI:** 10.1016/j.xgen.2022.100107

**Published:** 2022-03-09

**Authors:** Chongyuan Luo, Hanqing Liu, Fangming Xie, Ethan J. Armand, Kimberly Siletti, Trygve E. Bakken, Rongxin Fang, Wayne I. Doyle, Tim Stuart, Rebecca D. Hodge, Lijuan Hu, Bang-An Wang, Zhuzhu Zhang, Sebastian Preissl, Dong-Sung Lee, Jingtian Zhou, Sheng-Yong Niu, Rosa Castanon, Anna Bartlett, Angeline Rivkin, Xinxin Wang, Jacinta Lucero, Joseph R. Nery, David A. Davis, Deborah C. Mash, Rahul Satija, Jesse R. Dixon, Sten Linnarsson, Ed Lein, M. Margarita Behrens, Bing Ren, Eran A. Mukamel, Joseph R. Ecker

**Affiliations:** 1 Genomic Analysis Laboratory, The Salk Institute for Biological Studies, La Jolla, CA 92037, USA; 2 Howard Hughes Medical Institute, The Salk Institute for Biological Studies, La Jolla, CA 92037, USA; 3 Department of Human Genetics, University of California, Los Angeles, Los Angeles, CA 90095, USA; 4 Division of Biological Sciences, University of California, San Diego, La Jolla, CA 92037, USA; 5 Department of Physics, University of California, San Diego, La Jolla, CA 92037, USA; 6 Department of Cognitive Science, University of California, San Diego, La Jolla, CA 92037, USA; 7 Division of Molecular Neurobiology, Department of Medical Biochemistry and Biophysics, Karolinska Institutet, 17177 Stockholm, Sweden; 8 Allen Institute for Brain Science, Seattle, WA 98109, USA; 9 Ludwig Institute for Cancer Research, La Jolla, CA 92093, USA; 10 Center for Epigenomics, Department of Cellular and Molecular Medicine, University of California, San Diego, La Jolla, CA 92093, USA; 11 New York Genome Center, New York, NY 10013, USA; 12 Peptide Biology Laboratory, The Salk Institute for Biological Studies, La Jolla, CA 92037, USA; 13 Computational Neurobiology Laboratory, The Salk Institute for Biological Studies, La Jolla, CA 92037, USA; 14 Department of Neurology, Miller School of Medicine, University of Miami, Miami, FL 33136, USA; 15 Present address: Institute of Experimental and Clinical Pharmacology and Toxicology, Faculty of Medicine, University of Freiburg, 79104 Freiburg, Germany; 16 These authors contributed equally; 17 Senior author; 18 Lead contact

## Abstract

Single-cell technologies measure unique cellular signatures but are typically limited to a single modality. Computational approaches allow the fusion of diverse single-cell data types, but their efficacy is difficult to validate in the absence of authentic multi-omic measurements. To comprehensively assess the molecular phenotypes of single cells, we devised single-nucleus methylcytosine, chromatin accessibility, and transcriptome sequencing (snmCAT-seq) and applied it to postmortem human frontal cortex tissue. We developed a cross-validation approach using multi-modal information to validate fine-grained cell types and assessed the effectiveness of computational data fusion methods. Correlation analysis in individual cells revealed distinct relations between methylation and gene expression. Our integrative approach enabled joint analyses of the methylome, transcriptome, chromatin accessibility, and conformation for 63 human cortical cell types. We reconstructed regulatory lineages for cortical cell populations and found specific enrichment of genetic risk for neuropsychiatric traits, enabling the prediction of cell types that are associated with diseases.

## In brief

Single-cell profiling has enabled unbiased cell-type classification. Rigorous comparison of cell types defined by different modalities requires the joint measurement of multiple signatures in the same cell. Luo et al. have developed single-nucleus methylcytosine, chromatin accessibility, and transcriptome sequencing (snmCAT-seq) and applied it to categorize human brain cortical cell types.

## Graphical abstract

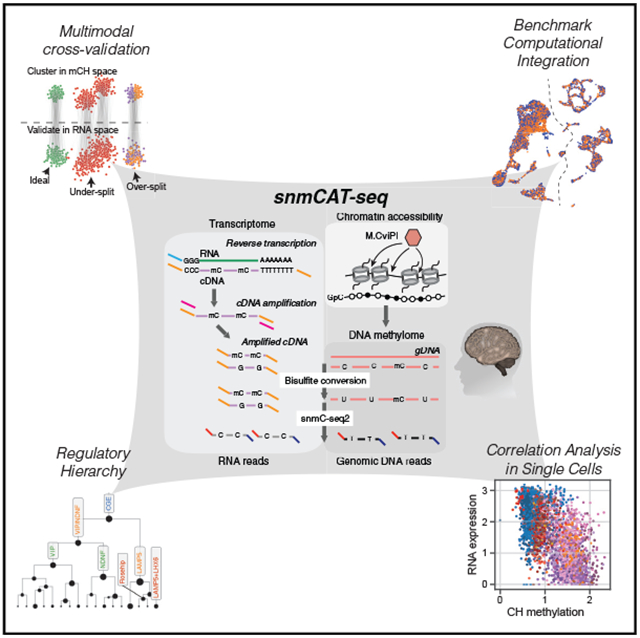

## INTRODUCTION

Single-cell transcriptome, cytosine DNA methylation (mC), and chromatin profiling techniques have been successfully applied for cell-type classification and studies of gene expression and regulatory diversity in complex tissues.^1,2^ The broad range of targeted molecular signatures, as well as technical differences between measurement platforms, presents a challenge for integrative analysis. For example, mouse cortical neurons have been studied using single-cell assays that profile RNA, mC, or chromatin accessibility,^3–7^ with each study reporting its own classification of cell types. Although it is possible to correlate the major cortical cell types identified by transcriptomic and epigenomic approaches, it remains unclear whether fine subtypes can effectively be integrated across different datasets and fused between modalities. Recently, computational methods based on canonical correlation analysis,^8^ mutual nearest neighbors,^9^ or matrix factorization^10^ have been developed to fuse molecular data types. However, validating the results of computational data fusion requires multi-omic reference data comprising different types of molecular measurements made in the same cell.

Single-cell multi-omics profiling provides a unique opportunity to evaluate cell-type classification using multiple molecular signatures.^1^ Most single-cell studies rely on clustering analysis to identify cell types. However, it is challenging to objectively determine whether the criteria used to distinguish cell clusters are statistically appropriate and whether the resulting clusters reflect biologically distinct cell types.^11^ We reasoned that genuine cell types should be distinguished by concordant molecular signatures of cell regulation at multiple levels, including RNA, mC, and open chromatin, in individual cells. Moreover, multi-omic data can uncover subtle interactions among transcriptomic and epigenomic levels of cellular regulation.

Existing methods for joint profiling of transcriptome and mC, such as scM&T-seq and scMT-seq, rely on the physical separation of RNA and DNA followed by parallel sequencing library preparation.^12–14^ Generating separate transcriptome and mC sequencing libraries leads to a complex workflow and increases cost. Moreover, it is unclear whether these methods can be applied to single nuclei, which contain much less polyadenylated RNA than whole cells. Because the cell membrane is ruptured in frozen tissues, the ability to produce robust transcriptome profiles from single nuclei is critical for applying a multi-omic assay for cell-type classification in frozen human tissue specimens.

Here, we describe a single nucleus multi-omic method snmCAT-seq (single-nucleus methylcytosine, chromatin accessibility, and transcriptome sequencing) that simultaneously interrogates transcriptome, mC, and chromatin accessibility without requiring the physical separation of RNA and DNA (see Table 1 for a glossary of genomic-profiling methods discussed in this study). We applied snmCAT-seq to cultured human cells and postmortem human frontal cortex tissues. We further generated an additional 23,005 single-nucleus, droplet-based RNA sequencing (RNA-seq) profiles (snRNA-seq, Table 1) and 12,557 single-nucleus, snATAC-seq-based (Table 1) open chromatin profiles using frozen human frontal cortex tissue.^5^ Using this comprehensive multimodal dataset, we developed computational strategies to tackle two challenges in single-cell biology: (1) how to assess the statistical and biological validity of clustering analyses and (2) how to validate computational approaches to fuse multiple single-cell data types. We then performed integrated analyses of single-cell methylomes for the human frontal cortex comprised of 15,030 cells, including two multi-omic datasets generated by snmCAT-seq and the previously published sn-m3C-seq, a method to simultaneously profile chromatin conformation and mC.^15^ These large datasets enabled the identification of gene-regulatory diversity for 63 finely defined brain cell types at an unprecedented level of data fusion using four levels of molecular signatures (i.e., transcriptome, methylome, chromatin accessibility, and conformation) to define their unique regulatory genomes with cell-type specificity and link them to genetic disease risk variants.

### Design

**Figure 1.**
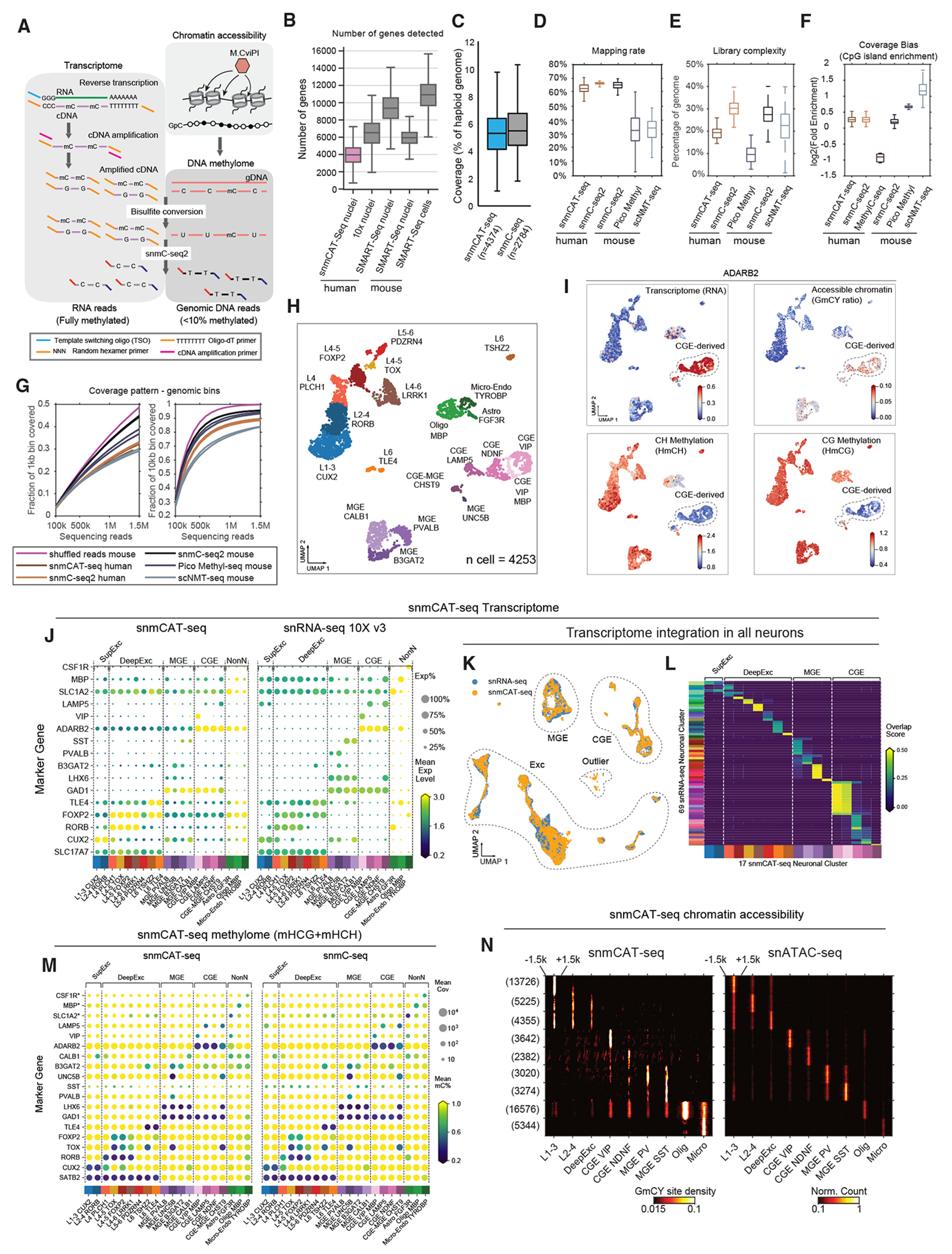
snmCAT-seq generates single-nucleus multi-omic profiles of the human brain (A) Schematic diagram of snmCAT-seq. (B) Boxplot comparing the number of genes detected in each cell or nucleus by different single-cell or single-nucleus RNA-seq technologies (C) Boxplot comparing the genome coverage of single-nucleus methylome between snmCAT-seq and snmC-seq. (D–G) snmCAT-seq methylome was compared to other single-cell methylome methods with respect to mapping rate (D), library complexity (E), enrichment of CpG islands (F), and coverage uniformity (G). (H) UMAP embedding of human frontal cortex snmCAT-seq profiles. (I) UMAP embedding of transcriptome, methylome, and chromatin accessibility profiled by snmCAT-seq for *ADARB2*. The cells are colored by gene expression (CPM, counts per million), chromatin accessibility (MAGIC imputed GmCY ratio, see STAR Methods), non-CG DNA methylation (HmCH ratio normalized per cell), and CG DNA methylation (HmCG ratio normalized per cell). (J) Comparison of marker gene expression between clusters identified using snmCAT-seq and matching clusters identified using snRNA-seq. The matching clusters were merged from original snRNA-seq clusters based on cell integration and label transfer (see STAR Methods). Dot sizes represent the fraction of cells with detected gene expression. Dot colors represent the mean expression level across the cells with detected gene expression. (K) UMAP embedding of snmCAT-seq transcriptome and snRNA-seq cells after integration. (L) Confusion matrix comparing snmCAT-seq clusters to snRNA-seq clusters. The plot is colored by overlapping scores between clusters. (M) Comparison of marker gene non-CG methylation (HmCH) between clusters identified using snmCAT-seq and matching clusters identified using snmC-seq. Dot sizes represent the mean cytosine coverage per cell. Dot colors represent the mean HmCH ratio. *For non-neuronal cell markers, gene body CG methylation (HmCG) levels were compared between snmCAT-seq and snmC-seq. (N) Comparison of chromatin accessibility profiled by snmCAT-seq and snATAC-seq at cell-type-specific open chromatin sites. The left and right heatmaps show the density of methylated GCY sites and the density of ATAC-seq reads, respectively. The elements of all boxplots are defined as the following: center line, median; box limits, first and third quartiles; whiskers, 1.5× interquartile range.

Simultaneous DNA methylcytosine and transcriptome sequencing using snmCAT-seq allows RNA and DNA molecules to be molecularly partitioned by incorporating 5’-methyl-dCTP (2’-deoxy-5-methylcytidine 5’-triphosphate) instead of dCTP (deoxycytidine triphosphate) during reverse transcription of RNA (Figure 1A). We treated single cells and nuclei with Smart-seq or Smart-seq2 reactions for *in situ* cDNA synthesis and amplification of full-length cDNA (Table 1).^18,20^ Replacing dCTP by 5’-methyl-dCTP results in fully cytosine-methylated double-stranded cDNA amplicons. Following bisulfite treatment converting unmethylated cytosine to uracil, sequencing libraries containing both cDNA- and genomic DNA-derived molecules were generated using snmC-seq2 (Table 1).^4,16^ With this strategy, all sequencing reads initially derived from RNA are completely cytosine methylated and do not show C-to-U sequence changes during bisulfite conversion. By contrast, more than 95% of cytosines in mammalian genomic DNA are unmethylated and converted by sodium bisulfite to uracils that are read during sequencing as thymine.^21^ In this way, sequencing reads originating from RNA and genomic DNA can be distinguished by their total mC density. Because 70%–80% of CpG dinucleotides are methylated in mammalian genomes, we used the read-level non-CG methylation (mCH) to uniquely partition sequencing reads into RNA or DNA bins. Specifically, we expect the level of mCH for all RNA-derived reads to be greater than 90%, while, for DNA-derived reads, the level is no more than 50% even considering the enrichment of mCH in adult neurons.^22^ Using this threshold, only 0.02% ± 0.01% of single-cell methylome reads (n = 100 cells profiled with snmC-seq2^23^) were misclassified as transcriptome reads and only 0.23% ± 0.17% of single-cell RNA-seq reads (n = 100 cells profiled with Smart-seq^6^) were misclassified as methylome reads (Figure S1A). For a snmCAT-seq profile containing 90% of methylome reads and 10% of transcriptome reads, the estimated specificity for classifying methylome and transcriptome reads is 99.997% and 99.97%, respectively. These results show that RNA- and DNA-derived snmCAT-seq reads can be effectively separated. We extended the multi-omic profiling to include a measure of chromatin accessibility by incorporating the nucleosome occupancy and methylome-sequencing assay (NOMe-seq; Figure 1A; Table 1).^14,17,24,25^ In the snmCAT-seq assay, regions of accessible chromatin are marked by treating bulk nuclei with the GpC methyltransferase M.CviPI prior to fluorescence-activated sorting of single nuclei into the reverse transcription reaction (Figure 1A). A detailed bench protocol for snmCAT-seq and future updates to the method can be found at https://www.protocols.io/view/snmcat-v1-bwubpesn.

## RESULTS

### Joint analysis of RNA and DNA methylome in cultured human cells

We first tested the efficacy of the joint profiling of RNA and DNA methylome by applying snmCAT-seq to either single whole cells or single nuclei of cultured human H1 embryonic stem cells and HEK293 cells (Tables S1 and S2), without the labeling of accessible chromatin using GpC methyltransferase. snmCAT-seq transcriptome profiling detected 4,220 ± 1,251 genes from single whole cells using exonic reads and 4,531 ± 1,888 genes using both exonic and intronic reads (Figure S1B). Similar to previously reported single-nuclei RNA-seq datasets, a minor fraction (17.3% ± 6.1%) of snmCAT-seq transcriptome reads generated from single nuclei were mapped to exons, whereas 68.1% ± 15.2% of snmCAT-seq reads generated from single cells were mapped to exons (Figure S1C). Transcriptome reads accounted for 22.2% ± 13.6% and 9.2% ± 6.5% of all mapped reads for snmCAT-seq data generated from single cells or nuclei, respectively (Figure S1D). The snmCAT-seq profiles could clearly separate H1 and HEK293 cells by their transcriptomic signatures^26^ (Figures S1E and S1F) and recapitulate specific gene expression signatures (Figure S1G).

To assess whether the two cell types could be distinguished using mC signatures derived from snmCAT-seq, we performed tSNE using the average CG methylation (mCG) level of 100 kb non-overlapping genomic bins (Figures S1H and S1I). As exemplified by the NANOG and CRNDE loci (Figure S1J), snmCAT-seq produced mC profiles highly consistent with data generated from bulk methylomes.^27^ snmCAT-seq data generated from both single cells and single nuclei identified global mC differences between H1 and HEK293T cells, showing that H1 cells are more methylated in both CG (83.6%) and non-CG (1.3%) contexts compared with HEK293T cells (mCG: 60.1%, no significant mCH detected, Figures S1K–S1N).^21^ To examine whether local mC signatures can be recapitulated in snmCAT-seq, we identified differentially methylated regions (DMRs) from bulk H1 and HEK293 methylomes. Plotting mCG levels measured using snmCAT-seq profiles across DMRs showed highly consistent patterns compared to bulk cell methylomes (Figures S1O and S1P).

### Multi-omic profiling of postmortem human brain tissue with snmCAT-seq

We generated snmCAT-seq profiles from 4,358 single nuclei isolated from postmortem human frontal cortex tissue from two young male donors (21 and 29 years old, Tables S3 and S4). The data quality was similar to datasets generated from nuclei isolated from cultured human cells with respect to the fraction of sequencing reads mapped to the transcriptome (Figure S2A), the fraction of transcriptome reads mapped to introns and exons (Figure S2B), and the number of genes detected (Figure 1B). Compared with snmC-seq and snmC-seq2 data generated from human single nuclei,^4,16^ the DNA methylome component of snmCAT-seq had comparable genomic coverage (Figure 1C) and mapping efficiency (Figure 1D) and showed only moderately reduced library complexity (Figure 1E) with similar coverage uniformity (Figures 1F and 1G).

To compare each data modality profiled by snmCAT-seq with their corresponding single-modality assays, we first identified 20 cell types by multi-modal clustering analysis using transcriptome, methylome, and chromatin accessibility. We used RNA abundance across the gene body for the transcriptome, mCH and mCG level of chromosome non-overlapping 100-kb bins, and binarized NOMe-seq signal of 5-kb bins for chromatin accessibility (see STAR Methods). For snmCAT-seq, the HCH context was counted for CH methylation, and HCG is counted for CG methylation to exclude GCH and GCG sites that can be methylated by M.CviPI. We identified highly variable features and calculated principal components separately for each modality. We observed substantial differences across data modalities in their ability to resolve cell populations using the top 10 principal components (Figure S2G). Therefore, only informative principal components from each data modality were concatenated as the input features for multi-modal clustering and visualization using uniform manifold approximation and projection (UMAP)^28^ of the three data types (Figures 1H and 1I). The selection of informative principal components for multi-modal clustering is agnostic to the type of molecular profile being analyzed and could be generalized to other multi-omic approaches. We found non-CG methylation as the most distinguishing measurement explaining 63.7% of the total variance, while CG methylation, RNA abundance, and NOMe-seq signal each explained 15.8%, 20.2%, and 0.4% of the variance, respectively (Figure S2C). These cell types were effectively separated by performing dimensionality reduction using each data type (Figures S2D–S2F). The comparison of homologous clusters between snmCAT-seq transcriptome and snRNA-seq (Table S5) shows a robust global correlation: Pearson r = 0.82 for both parvalbumin (PV)-expressing inhibitory neurons (medial ganglionic eminence [MGE]_PVALB, p = 1 × 10^−145^) and superficial layer excitatory neurons (L1-3 CUX2, p = 3 × 10^−301^) (Figures S2H and S2I). Moreover, highly consistent expression patterns of cell-type signature genes were observed (Figure 1J).

To test whether snmCAT-seq transcriptome data can be integrated with snRNA-seq (Table 1), we integrated snRNA-seq and the transcriptome component of snmCAT-seq using a mutual nearest neighbor approach^29^ (Figures 1K and 1L). The integration confirmed that the cell types identified using the snmCAT-seq transcriptome are strongly correlated with the cell types found using snRNA-seq. Similar to the transcriptome, both mCH and mCG profiles correlate strongly between methylomes generated with snmCAT-seq and snmC-seq2 either globally (Figures S2J and S2K) or at cell-type-specific signature genes (Figure 1M).

The presence of high levels of mCH in the human brain confounds the analysis of chromatin accessibility using methylation at GpC sites (GmC). However, we found that in GCT and GCC sequence contexts, GmC introduced by M.CviPI greatly surpasses the levels of native methylation by 6.4- and 16-fold, respectively (Figure S2L). Thus, for snmCAT-seq, we focused our analyses of chromatin accessibility on GmC at GCY (Y = C or T) sites in the genome. We further developed a computational strategy to first identify significantly methylated GCY (GmCY) sites using a hidden Markov model approach^30^ followed by the calling of open chromatin regions using the frequency of GmCY sites. Chromatin accessibility measured by the frequency of GmCY sites correlates closely with snATAC-seq signal at cell-type-specific open chromatin sites both globally (Figures 1N, S2M, and S2N, p value < 2.2 × 10^−308^) and at cell-population-specific genes such as *BDNF, POU3F2, DLX2/3*, and *SOX11* (Figure S2O). In addition, open chromatin regions identified with GmCY frequency overlapped substantially with regions found using snATAC-seq (Figures S2P and S2Q). In summary, snmCAT-seq can simultaneously profile transcriptome, methylome, and chromatin accessibility in single nuclei, accurately recapitulating cell-type signatures for each data type.

### Paired RNA and mC profiling enables cross-validation and quantification of over-/under-splitting for single-cell clusters

**Figure 2.**
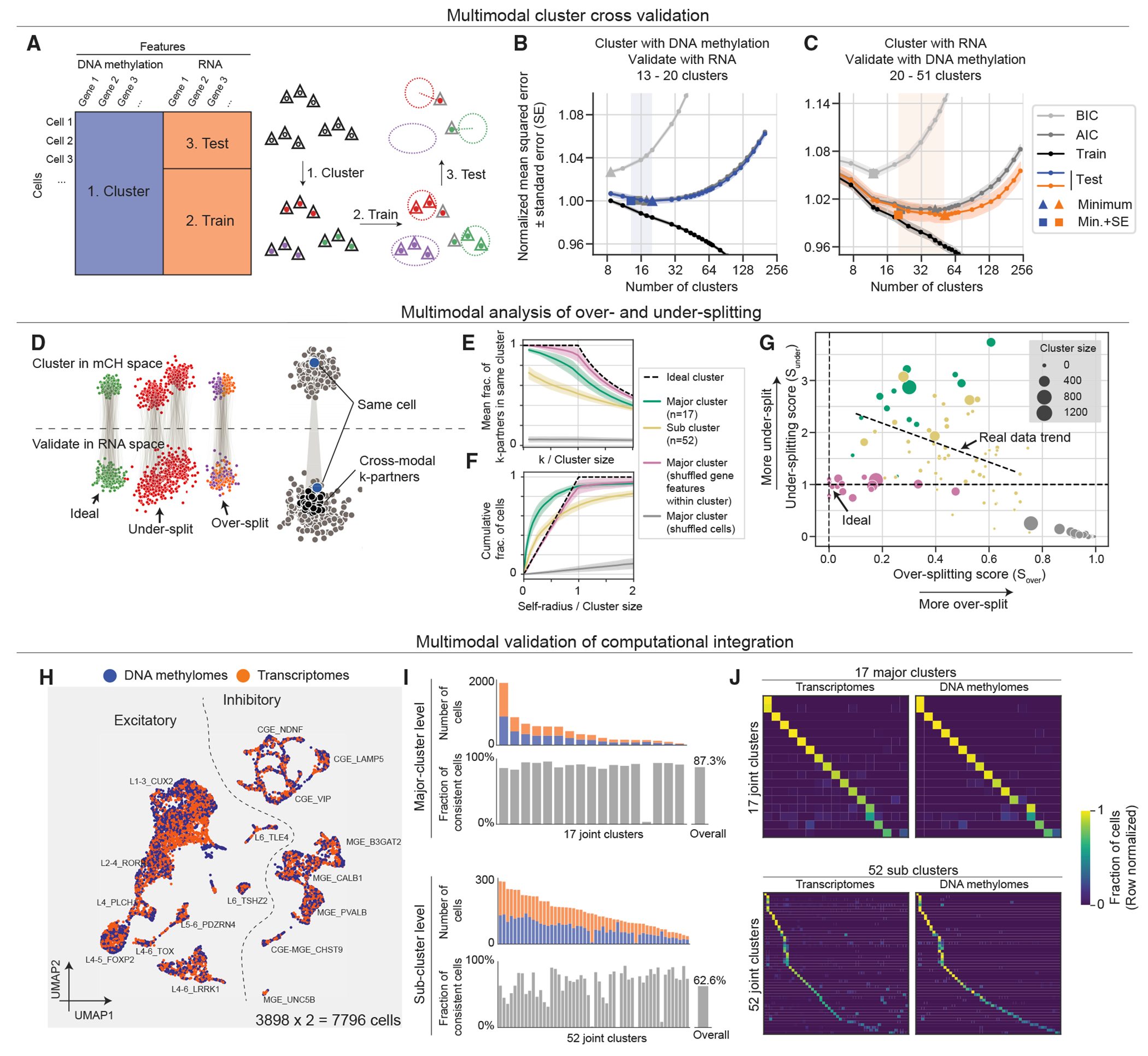
Integrative analysis of RNA and mC features cross-validates neuronal cell clusters (A) Schematic diagram of the cluster cross-validation strategy using matched single-cell methylome and transcriptome profiles. (B and C) Mean squared error, Akaike information criterion (AIC), and the Bayesian information criterion (BIC) between RNA expression profile (B) or mCH (C) of individual cells and cluster centroids were plotted as a function of the number of clusters. The shaded region in each plot highlights the range between the minimum and the minimum + standard error for the curve of test-set error. Cross-validation analysis was performed in reciprocal directions by performing Leiden clustering using mC (B) or RNA (C) profiles followed by cross-validation using the matched RNA (B) and mC (C) data, respectively. (D) Schematic diagram of the over- and under-splitting analysis using matched single-cell methylome and transcriptome profiles. (E) Over-splitting of mC-defined clusters was quantified by the fraction of cross-modal *k*-partners found in the same cluster defined by RNA. Shades indicate confidence intervals of the mean. (F) Under-splitting of clusters was quantified as the cumulative distribution function of normalized self-radius. (G) Scatterplot of over-splitting (S_over_) and under-splitting (S_under_) scores for all neuronal clusters. Dot sizes represent cluster size. The actual data trend shows a linearly regressed line on both major clusters and sub-clusters. (H) Joint UMAP visualization of snmCAT-seq transcriptome and methylome by computational fusion using the SingleCellFusion method, assuming snmCAT-seq transcriptomes and methylome were derived from independent datasets. (I) Accuracy of computational fusion determined by the fraction of cells with matched transcriptome and epigenome profile grouped in the same cluster. (J) Confusion matrix normalized by each row. Each row shows the fraction of cells from each joint cluster that are from each cluster defined in Figure 4. Transcriptomes and DNA methylomes are quantified separately.

A fundamental challenge for single-cell genomics is to objectively determine the number of biologically meaningful clusters in a dataset.^11^ Cross-dataset integration of the same data type or fusion of distinct data types can be used to assess cluster robustness, but it may be limited by systematic differences between the datasets or modalities used.^31^ To address this, we devised a novel cross-validation procedure using matched transcriptome and DNA methylation information to estimate the number of reliable clusters supported by both modalities in snmCAT-seq data (3,898 neurons, Figure 2A). We first clustered the cells with different resolutions using mC information, then tested how well each clustering is supported by the matched transcriptome profiles. We used the cross-validated mean squared error between the RNA expression profile of individual cells and the cluster centroid as a measure of cluster fidelity (Figures 2B and 2C). Mean squared error for cells in the training set decreased monotonically with the number of clusters, whereas over-clustering leads to an increase in mean squared error for the test set. The U-shaped mean squared error curve shows that aggressively splitting cells into fine-scale clusters based on mC signatures is not supported by corresponding RNA signatures. The cluster resolution with the minimum mean squared error represents the finest subdivision of cells that is well supported across both modalities. In addition to directly evaluating error on a test set, the Akaike information criterion (AIC) and the Bayesian information criterion (BIC) of the training set were also applied to estimate test error (see STAR Methods; Figures 2B, 2C, and S3A). Indeed, AIC curves largely overlapped with test errors and gave similar estimates of the optimum cluster numbers; BICs consistently reach smaller optimums than the other two metrics as they penalize model complexity more stringently. Using these approaches, we found a range of 20–50 clusters with strong multimodal support in the current snmCAT-seq dataset (Figures 2B and 2C). The same approach can also be applied to each individual modality separately to identify the number of clusters supported by DNA methylation features and by RNA features, respectively (Figure S3A).

The above-mentioned approach objectively identified a range of appropriate cluster resolutions for the whole dataset. To assess the quality of individual clusters, we further developed metrics to quantify over-splitting and under-splitting (STAR Methods; Figures 2D and S3B). After jointly embedding mC and RNA data in a common low-dimensional space,^32^ we defined a graph connecting each cell to *k* cells with the greatest cross-modality similarity (called *k*-partners). An over-splitting score was calculated as the fraction of each cell’s *k*-partners that are not in the same cluster (STAR Methods; Figures 2D and 2E). We assessed the over-splitting of 17 major neuronal clusters and 52 neuronal sub-clusters (Figure 4; Table S6) identified by single-cell methylomes and found that major clusters resemble ideal, homogeneous clusters (simulated by shuffling gene features) with low over-splitting scores (Figures 2E, S3C, and S3E), with only 1/17 major clusters having an over-splitting score ≥ 0.6. Most sub-clusters also had relatively little over-splitting; only 10/52 sub-clusters had an over-splitting score ≥ 0.6 (Figures S3C and S3E).

To assess under-splitting, we reasoned that if a cluster cannot be further split (no under-splitting), all its cells should be statistically equivalent. Therefore, each cell’s mC profile should be no more correlated with its own RNA profile than with the RNA profile of any other cell of the same type. By contrast, an under-split cluster will contain some residual discrete or continuous variation that is correlated between modalities. We tested this by defining the self-radius (the distance between mC and RNA profiles of the same cell; see STAR Methods) for each cell and comparing the distribution of self-radii for each cluster with that expected for homogeneous clusters using a permutation procedure. We found that major neuronal clusters had substantial within-cluster variation across cells, indicating that they are under-split (Figures 2F, S3D, and S3F). By contrast, subtypes resembled ideal (shuffled) clusters to a greater degree. Combining both scores, we quantitatively mapped the lumper-splitter tradeoff in terms of the degree of over- and under-splitting for each major type or subtype (Figure 2G).

The fusion of single-cell genomic data across multiple data types has been a focus of recent computational studies, yet existing methods lack validation on ground truth from experimental single-cell multi-omic datasets.^33^ By treating snmCAT-seq transcriptome and mC profiles as if they were generated from different single cells, we could test the performance of computational data fusion using Seurat,^8^ Harmony,^34^ Scanorama,^9^ LIGER,^10^ and SingleCellFusion (STAR Methods; Figures 2H and S3G–S3K, first row). To evaluate the fusion at the cell level, we calculated the self-radius as mentioned above and determined mis-fused events by normalized self-radius >0.3 (Figures S3G–S3K, second row). We also quantified the cluster level accuracy as the fraction of cells whose transcriptome and mC profiles were assigned to the same cluster (Figures 2I and S3G–S3K, third row). Overall, SingleCellFusion and Seurat outperform the other tools, with SingleCellFusion achieving the lowest mis-fusion ratio (5.7%) and highest overall major celltype-level accuracy (87.3%) (Figures 2I, 2J, and S3G). We also tested the SingleCellFusion accuracy at the subtype level. As expected, computational fusion of fine-grain clusters was less accurate (62.6%) and more variable across clusters (Figure 2I), potentially because of the greater degree of over-clustering (Figure 2E).

### Diverse correlation between gene body mCH and gene expression

**Figure 3.**
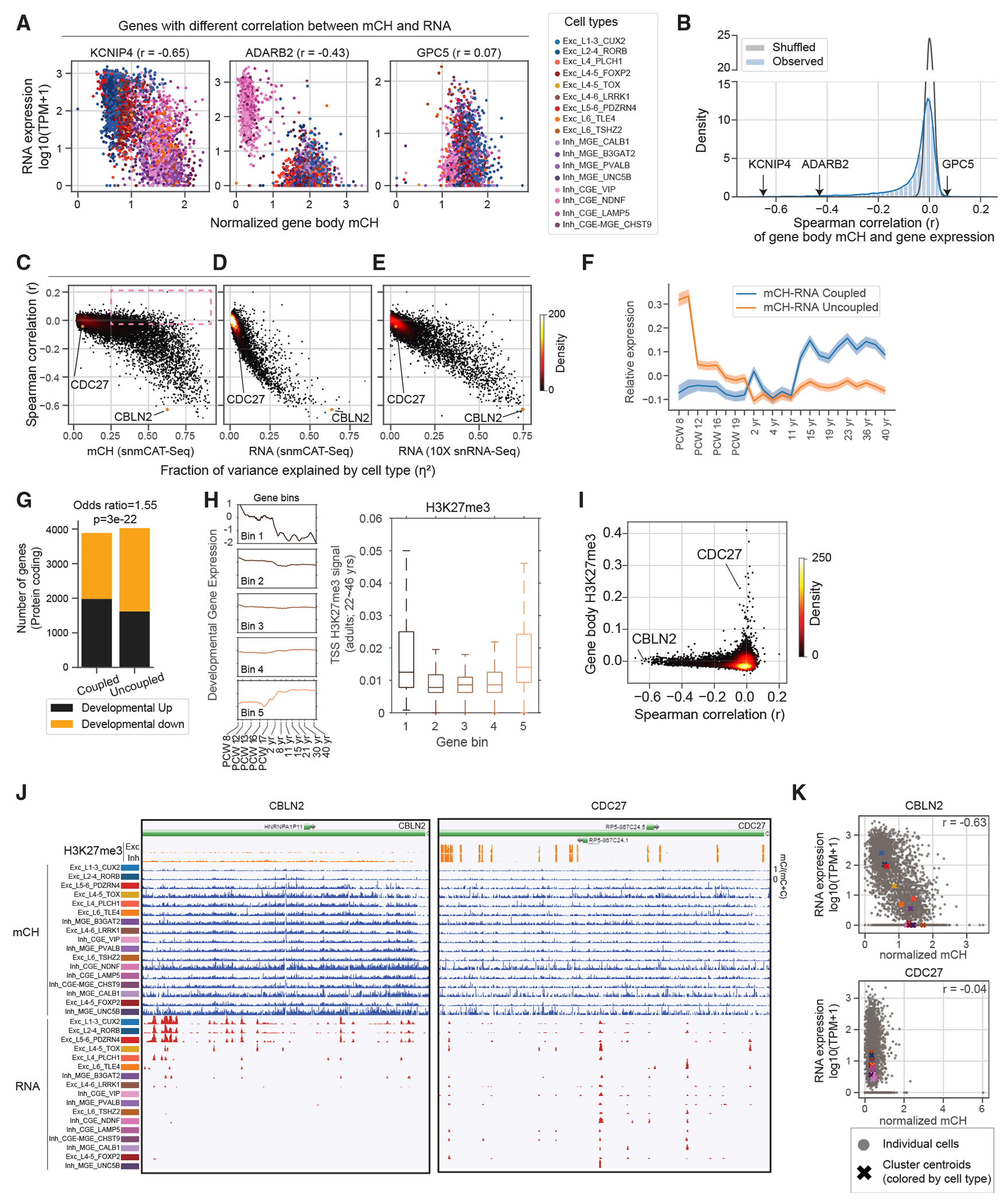
Single-cell correlation analysis of RNA expression and gene body non-CG methylation (A) Scatterplots of gene body mCH (normalized by the global mean mCH of each cell) and gene expression (log_10_(TPM+1)) of example genes (KCNIP4, ADARB2, GPC5) across all neuronal cells. Cells are colored by major cell types defined in Figure 4. The Spearman correlation coefficient (r) is shown for each example gene. (B) Distribution of Spearman correlation coefficient between gene expression and gene body mCH. Blue represents the actual distribution; gray represents the distribution with randomly shuffled cell labels. (C–E) Scatterplot of correlation coefficient of gene body mCH and RNA versus the fraction of variance explained by cell type (*η*^2^) from 3 different datasets/features: snmCAT-seq mCH (C), snmCAT-seq RNA (D), and snRNA-seq (E). (F) Line plot of mean relative expression over developmental time points with 2 different gene groups (mCH-RNA coupled in blue; mCH-RNA uncoupled in orange). Relative expression level is defined as the log2(RPKM) minus mean log2(RPKM) over all time points for each gene. Shaded areas indicate the standard error of the mean. (G) Barplot of the number of protein coding genes in each of the 4 categories according to whether it’s developmentally up- or downregulated and whether its mCH-RNA is coupled or not. (H) Left: line plots of mean relative expression level over developmental time points for 5 gene bins. Genes are binned by gene expression ratio between early fetal (PCW 8–9) and adult (>2 years). Right: boxplot of TSS H3K27me3 signals at each of the 5 gene bins. (I) Scatterplot of Spearman correlation of gene body mCH and gene expression versus the mean H3K27me3 signal in neurons at gene-body level. The H3K27me3 ChIP-seq data are from purified glutamatergic and GABAergic neurons from human frontal cortex.^36^ (J) Genome browser track visualization of CBLN2 (mCH-RNA coupled) and CDC27 (uncoupled). (K) Gene-level signal of CBLN2 and CDC27: scatterplot of normalized gene body mCH versus gene expression for all neuronal cells. Raw mCH level is normalized by the global mean mCH level of each cell. The elements of all boxplots are defined as the following: center line, median; box limits, first and third quartiles; whiskers, 1.5× interquartile range.

Using the paired profiling of transcriptome and mC by snmCAT-seq, we found diverse patterns of correlation between mCH and gene expression across thousands of single cells. Figure 3A shows examples of three distinct types of correlations between gene body mCH and gene expression. *KCNIP4* shows an inverse correlation between mCH and RNA across a broad range of cell types. *ADARB2* is a marker gene for caudal ganglionic eminence (CGE)-derived inhibitory cells and showed a strong inter-cluster correlation but no intra-cluster correlation between mCH and RNA. Finally, *GPC5* has a gradient of mCH across clusters (low in CGE VIP [asoactive Intestinal Polypeptide] expressed neurons, high in L1-3 CUX2) but no corresponding pattern of differential gene expression across cell types. Applying this correlation analysis to all 13,637 sufficiently covered genes, we found that 38% (n = 5,145) have a significant negative correlation between mCH and RNA (mCH-RNA coupled, FDR < 5%). The majority of genes (62%) had no apparent correlation that could be distinguished from noise (mCH-RNA uncoupled, Figure 3B). The pattern of correlation was highly consistent between the specimens we profiled and robust with respect to normalization and data smoothing (Figures S4A–S4H). We found that mCH-RNA correlation is correlated (r = 0.63) with mCG-RNA correlation, consistent with previous findings^4,35^ (Figure S4I). Genes with a significant correlation between mCH and gene expression are longer, are more highly expressed, show greater chromatin accessibility, and are enriched in neuronal functions (Figures S4J–S4M).

We further investigated the factors that determine the degree of correlation between mCH and RNA for each gene. We reasoned that housekeeping genes with a strong expression and little variation across cell types would show weak mCH-RNA correlation, whereas mCH-RNA coupling is enriched in genes with cell-type-specific expression. We quantified the cell-type specificity of gene expression and DNA methylation by calculating the fraction of variance in gene expression explained by cell type (*RNA η*^2^ and *mCH η*^2^; Figures 3C–3E and S4N). Consistent with our hypothesis, genes with greater *RNA η*^2^ had a stronger inverse correlation between mCH and RNA (Figures 3D and 3E). Notably, we found a large number of genes (n = 1,243) with strong gene body mCH diversity across cell types (*mCH η*^2^> 0.25) but no apparent correlation between mCH and RNA (r < −0.03) (box in Figure 3C). This suggests that the lack of correlation between mCH and gene expression is driven by variability in gene expression within cell types despite conserved DNA methylation signatures.

The accumulation of mCH in the frontal cortex starts from the second trimester of embryonic development and continues into adolescence.^22,37^ The developmental dynamics of mCH motivated us to compare the developmental expression of mCH-RNA coupled and uncoupled genes. We found that mCH-RNA uncoupled genes, on average, are highly expressed during early fetal brain development (postconceptional weeks [PCW] 8–9) and are later repressed, whereas the expression of mCH-RNA coupled genes is moderately increased during development (Figure 3F). Consistently, developmentally downregulated genes are significantly enriched in the mCH-RNA uncoupled group (Figure 3G). We speculated that the developmentally downregulated genes may be repressive by alternative epigenomic marks such as histone H3K27 trimethylation (H3K27me3), which leads to the uncoupling of RNA and gene body mCH. By binning all the genes by their expression dynamics during brain development, we indeed found that the promoters of both down- and upregulated genes are enriched in H3K27me3 and depleted in active histone marks (Figures 3H and S4O). We directly compared mCH-RNA correlation and H3K27me3 in purified human cortical glutamatergic and GABAergic neurons^36^ and found that genes with strong H3K27me3 signal clearly show weak correlations between gene body mCH and gene expression (e.g., CDC27; Figures 3I–3K). In summary, although mCH and gene expression are clearly inversely correlated at a global scale, substantial variations can be observed from genes to genes at a single-cell level and can be partially explained by the presence of alternative epigenetic pathways such as polycomb repression.

### Multi-omic integration of chromatin conformation, transcriptome, methylome, and chromatin accessibility

**Figure 4.**
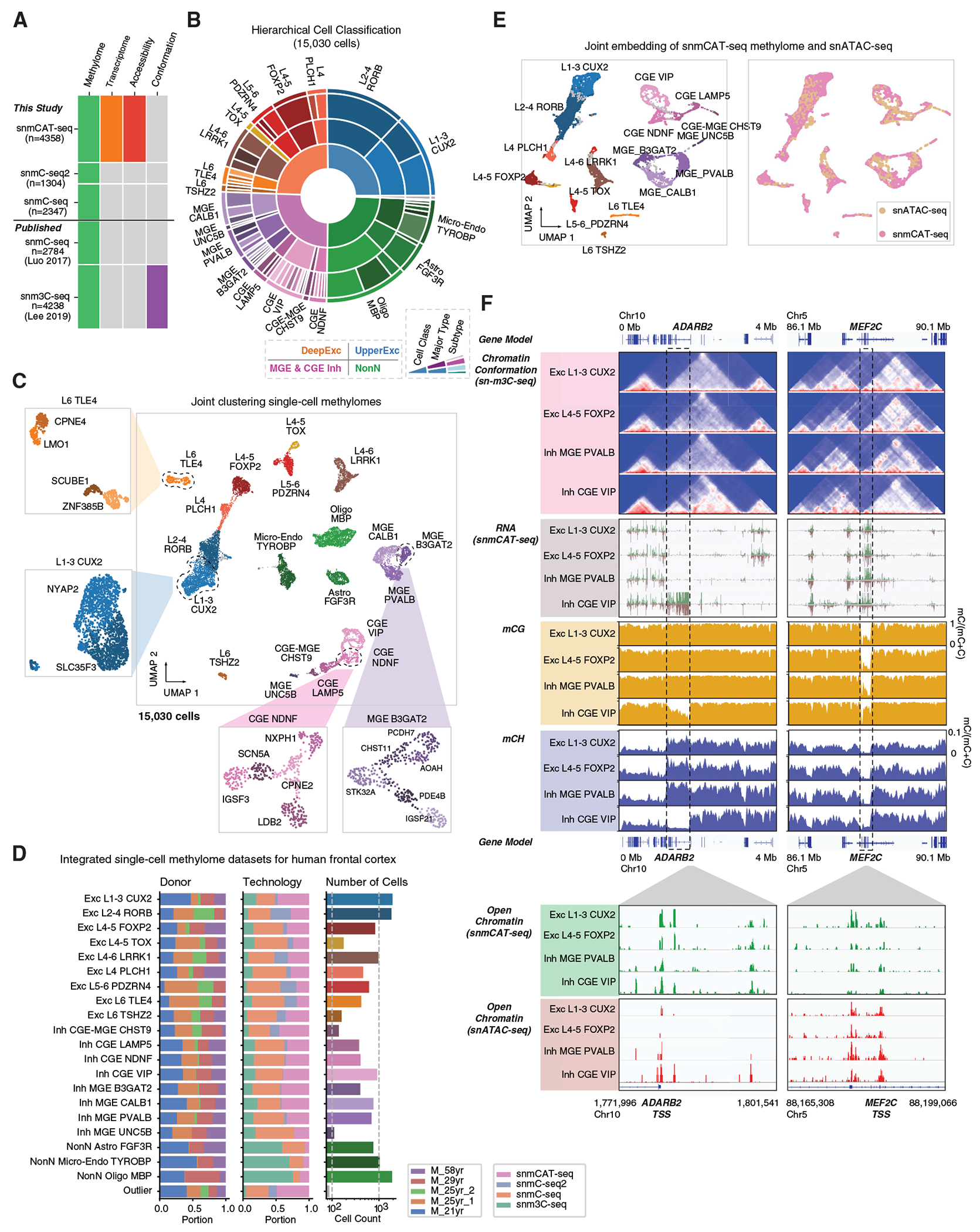
Integrated epigenomic atlas of the human frontal cortex (A) Methylome-based technologies and datasets included in the integrative analysis. (B) Sunburst visualization of the two-level methylome ensemble clustering analysis. The 4 cell classes (inmost ring) and 20 major cell types (middle ring and outer annotation) are identified in level 1 analysis, and the 63 subtypes are identified in level 2 analysis. (C) UMAP embedding of 15,030 cells colored and labeled by major cell types from level 1 analysis. Several examples of level 2 analysis are shown in insets with UMAP colored and labeled by subtypes. (D) Donor (left) and technology (middle) composition and cell count (right) of each major cell type. (E) UMAP embedding of the cross-modality fusion of snmCAT-seq methylome and snATAC-seq profiles. The left panel is colored and labeled by level 1 major cell types; the right panel is colored and labeled by the technologies. (F) Browser views of multi-modal data integration for *ADARB2* and *MEF2C* gene in four major cell types.

The snmCAT-seq dataset for the human frontal cortex was combined with previously published human frontal cortex datasets (Table S3): sn-m3C-seq, which simultaneously profiles mC and chromatin conformation,^15^ and snmC-seq methylomes for single neurons.^4^ We additionally generated new snmC-seq and snmC-seq2 data for the frontal cortex from two independent donors (Table S3). These datasets can be readily integrated using single-nucleus methylomes as the common modality (Figure 4A). To identify both major cell types and subtypes of frontal cortex, we integrated 15,030 single-cell methylomes generated by snmC-seq (n = 5,131), snmC-seq2 (n = 1,304), snmCAT-seq (n = 4,358), and sn-m3C-seq (n = 4,238) prior to the clustering analysis (Table S6). We used an iterative clustering approach to identify 20 major cell populations including 9 excitatory neuron types, 8 inhibitory neuron types, and 3 non-neuronal cell types in the first round of clustering (Figures 4B and 4C). A second round of iterative clustering of each major cell type identified 63 cell subtypes, including 19 excitatory neuronal subtypes, 33 inhibitory neuronal subtypes, and 11 non-neuronal cell subtypes (Figures 4B and 4C). Each fine-grained cell subtype can be distinguished from any other cell type by at least 10 mCH signature genes for neuronal clusters or 10 mCG signature genes for non-neuronal clusters. Consistent with our previous results,^4^ as well as transcriptomic studies,^38^ we found greater diversity among human cortical inhibitory neurons than among excitatory cells (Figure 4C). The methylome data generated by these diverse multi-omic methods and from multiple donors were uniformly represented in major cell type and subtype clusters (Figure 4D).

We next performed fusion of single-cell methylome and snATAC-seq (Figure 4E; Table S7) profiles by transferring the cluster labels defined by mC into ATAC-seq cells using a nearest neighbor approach^29^ that was adapted for epigenomic data and implemented in a new software package (https://github.com/mukamel-lab/SingleCellFusion; see STAR Methods). For each cell population, we reconstructed four types of molecular profiles: transcriptome (from snmCAT-seq), methylome (from snmC-seq1/2 and sn-m3C-seq), chromatin accessibility (from snmCAT-seq mGCY frequency or snATAC-seq), and chromatin conformation sn-m3C-seq^15,39^ (Figure 4F). This integrative analysis revealed extensive correlations across epigenomic marks at cell-type signature genes. For example, *ADARB2* is a signature gene of inhibitory neurons derived from the CGE. In CGE-derived VIP neurons, *ADARB2* was associated with abundant transcripts, reduced mCG and mCH, and distinct chromatin interactions compared with other neuron types (Figure 4F). In contrast, in VIP neurons, the *MEF2C* locus showed lower transcript abundance (TPM [transcripts per million], L1-3 CUX2: 75.8; L4-5 FOXP2: 80.2; MGE PVALB: 77.5; CGE VIP: 49.0), reduced chromatin interaction, and more abundant gene body mCG (Figure 4F). Although nearly identical open chromatin sites were identified at the promoter regions of *ADARB2* and *MEF2C* using snmCAT-seq GpC methylation and snATAC-seq, the two methods revealed distinct cell-type specificity of chromatin accessibility. At the *ADARB2* promoter, snATAC-seq, but not the snmCAT-seq GpC methylation profile, showed enriched chromatin accessibility in VIP neurons. However, at the *MEF2C* promoter, snmCAT-seq GpC methylation indicated a depletion of open chromatin in VIP neurons, which is more consistent with the reduced gene expression and increased gene body mCG in this inhibitory cell population. The cause of these differences in measures of chromatin accessibility is not clear, and further work is needed to clarify their respective sensitivities and biases.^30^

### snmCAT-seq identifies RNA and mC signatures of neuronal subtypes

**Figure 5.**
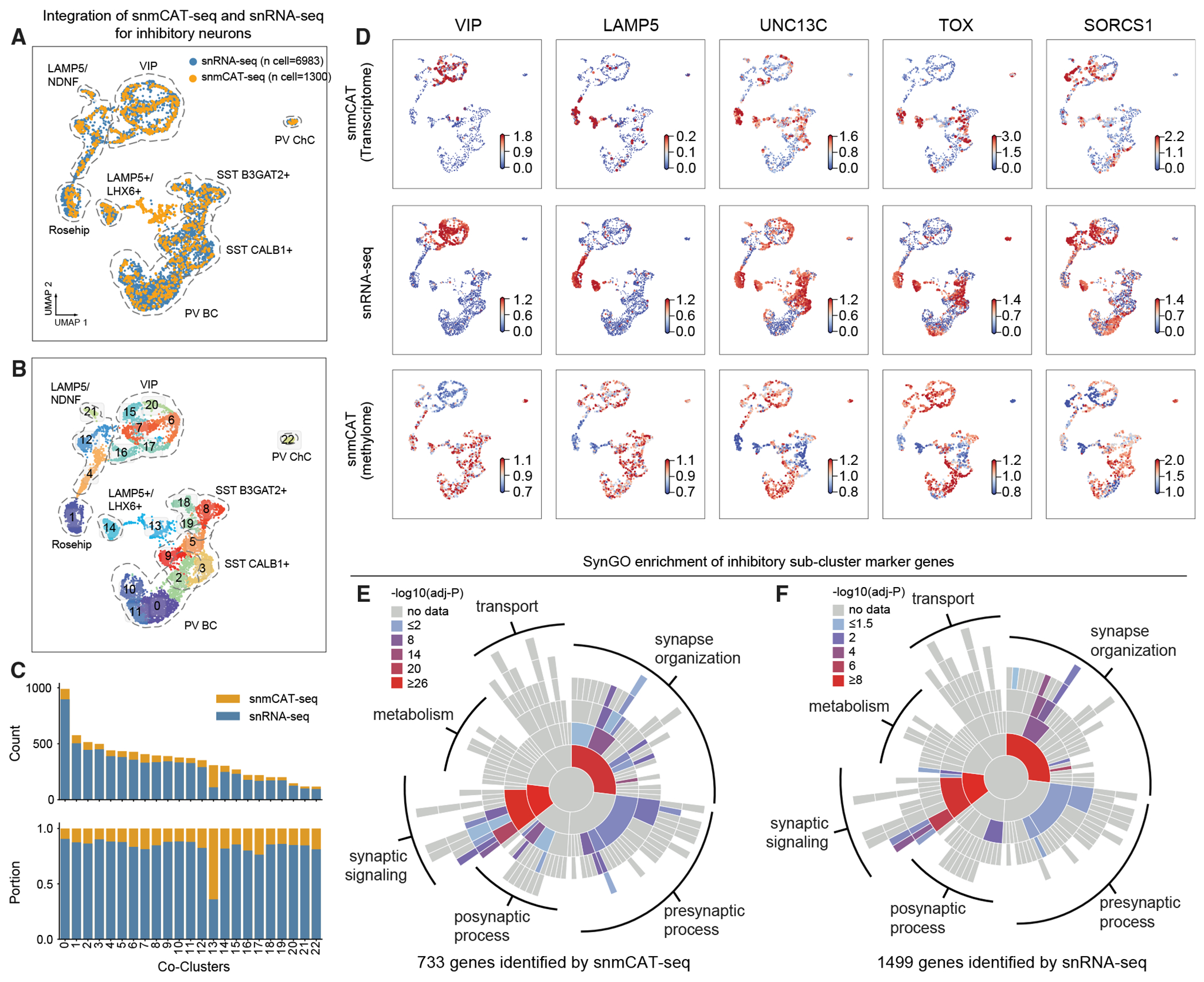
snmCAT-seq identifies RNA and mC signatures of neuronal subtypes (A and B) UMAP embedding of snmCAT-seq transcriptome and snRNA-seq for all the inhibitory neurons after mutual nearest neighbor (MNN)-based integration with the cells colored by technology (A) and joint clusters (B). (C) The composition of cells profiled by snmCAT-seq and snRNA-seq in inhibitory neurons joint clusters (same cluster IDs as shown in B). The upper and lower barplots show the counts and portion of cells profiled by the two technologies in each joint cluster, respectively. (D) Normalized expression and gene body mCH rate of inhibitory neuron subtype marker genes quantified using snmCAT-seq and snRNA-seq. (E and F) Sunburst visualization of inhibitory cell-type marker gene enrichment in SynGO biological process terms. Each sector is a SynGO term colored by -log10(adjusted p value) of snmCAT-seq transcriptome marker gene (E) or snRNA-seq marker gene (F) enrichment.

The integration of 15,030 single-cell methylomes allowed the determination of fine-grained brain cell subtypes with a sensitivity comparable to snRNA-seq (Figures 4B and 4C). For example, we identified 15 subtypes of CGE-derived inhibitory neurons using single-cell methylomes, whereas 26 subtypes were identified by snRNA-seq.^38^ To find whether snmCAT-seq can recapitulate the molecular signatures of neuronal subtypes, we integrated snmCAT-seq transcriptome with snRNA-seq datasets for inhibitory neurons followed by joint clustering (Figures 5A, 5B, and S5A). Individual nuclei profiled with snmCAT-seq transcriptome and snRNA-seq were uniformly distributed across joint clusters except for cluster 13 (Figures 5B and 5C), suggesting that, in general, the snmCAT-seq transcriptome recapitulates the full range of inhibitory neuron diversity. Cluster 13 contained snmCAT-seq data with lower numbers of transcriptome reads (Figure S5B), but the methylome profiles of the same cells showed acceptable quality and were robustly co-clustered with other inhibitory neurons (Figures S5B and S5C). Similarly, integration of snmCAT-seq transcriptomes and snRNA-seq for excitatory neurons and non-neuronal cells showed that brain-cell-type diversity across all cell classes can be recapitulated from the snmCAT-seq transcriptome profiles (Figures S5D–S5K). We further compared the expression of a panel of signature genes for inhibitory neuron subpopulations and found that snmCAT-seq transcriptome and snRNA-seq identified highly consistent expression patterns (Figure 5D). Lastly, we identified cell-type marker genes across inhibitory neuronal populations using transcriptome profiles generated with either snmCAT-seq or snRNA-seq (Table S8). Analysis of the marker genes using a database curated for neuronal functions, SynGO,^40^ revealed consistent enrichment in ontological categories associated with synaptic signaling and synapse organization for inhibitory neuron marker genes identified with both snmCAT-seq transcriptome and snRNA-seq data (Figures 5E and 5F).

### DNA methylation signatures of hierarchical transcription factor regulation in neural lineages

**Figure 6.**
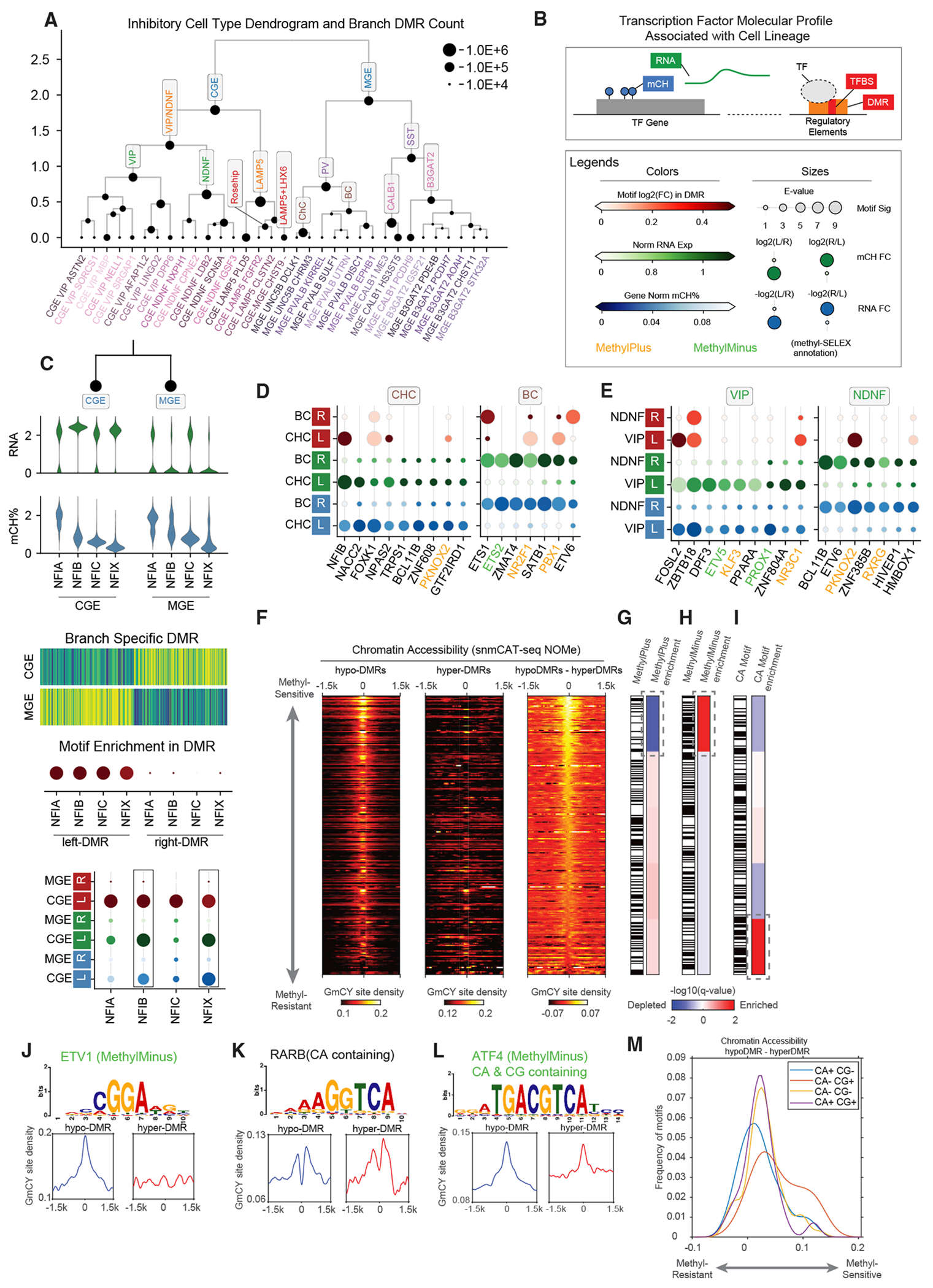
DMR phylogeny and transcription factor hierarchy in the human cortex (A) Inhibitory neuron subtype dendrogram. The node size represents the number of DMRs detected between the left and right branches. Nodes corresponding to known inhibitory cell type groups are annotated in the dendrogram. (B) Schematics of the three levels of molecular information we use to identify candidate TFs related to the specific lineage. (C) The workflow of TF analysis using the NFI family as an example. Three types of information are gathered for each of the TF genes: (1) RNA expression, (2) gene body mCH level, and (3) TF motif enrichment in the branch-specific DMR. We create a combined dot plot view for all three kinds of information; the genes that show lineage specificity in both 1 and 2 are circled by black boxes. (D and E) The dot plot view for TFs showing ChC versus BC (D) or VIP versus NDNF (E) specificity in motif enrichment, RNA, or mCH levels. Colors for every two rows from top to bottom: TF motif enrichment log2(fold change), branch mean expression log(1 + CPM), lineage mean gene body mCH level. Sizes for every two rows from top to bottom: E value of the motif enrichment test, relative fold change of expression level, relative fold change of mCH level between the two branches. Colors for the motif names: TF motif methylation preference annotated by methyl-SELEX experiment,^45^ orange indicates MethylPlus, green indicates MethylMinus. (F) The binding of TFs to hypermethylated regions validated by chromatin accessibility measurement using the snmCAT-seq NOMe-seq profile. (G–I) Enrichment or depletion of MethylPlus TFs (G), MethyMinus TFs (H), and TFs whose binding motif contains CA dinucleotides (I). (J–L) Examples of chromatin accessibility profiles at the binding motifs of ETV1 (MethylMinus) (J), RARB (motif contains CA) (K), and ATF4 (motif contains CA and CG) (L). (M) Comparison of the chromatin accessibility at the binding motifs containing CA or CG dinucleotides.

Temporally regulated expression of transcription factors (TFs) during specific developmental stages is critical for neuronal differentiation.^41,42^ We hypothesized that the cell-type hierarchy reconstructed from mC information reflects the developmental lineage of human cortical neurons. If so, then key transcription factors that specify neuronal lineage can be identified for each branch of the hierarchy. We separately constructed hierarchies for inhibitory and excitatory neurons based on the concatenated principal components of mCH and mCG (Figures 6A and S6A). The inhibitory neuron hierarchy comprises two major branches corresponding to MGE- and CGE-derived cells (Figure 6A). These major populations contain intermediate neuronal populations, such as PVALB-expressing basket dell (BC) and chandelier cell (ChC), or the recently reported LAMP5-expressing Rosehip neurons (Figure 6A).^43^ At the finest level, the hierarchy contains 33 neuronal subtypes (Figure 6A). To identify TFs involved in the specification of neuronal lineages, we compared three levels of molecular information for each of 1,639 human TFs^44^ between the daughter branches (Figure 6B). To assess the genome-wide DNA binding activity of the TF at regulatory elements, we used enrichment of DNA binding sequence binding motifs in differentially methylated regions (DMRs). To assess TF gene expression, we used both mRNA expression and TF gene body mCH level.

Our integrated strategy taking advantage of matched information for TF motif enrichment, transcript abundance, and TF gene body mCH level allowed us to distinguish the relative importance of closely related TFs sharing a common binding motif based on their cell-type-specific expression^46^ (Figure 6C). For example, we predicted that NFIB and NFIX contribute to CGE lineage specification because they show greater RNA abundance and stronger gene body mCH depletion than closely related TFs NFIA and NFIC. We systematically applied this approach across the excitatory neuron hierarchy (Figures S6B–S6D) and the inhibitory neuron hierarchy (Figures S6E–S6H), using 579 curated motifs from the JASPAR 2018 CORE vertebrates database.^47^ Many predicted lineage regulators were homologous to cell-type lineage regulators in mouse cortical development, such as NFIX and NFIB for CGE-derived neurons (Figure S6E), or LHX6, SOX6, and SATB1 for MGE-derived neurons (Figure S6E).^42,48^ The motifs of some TFs were also recurrently enriched in multiple lineages. For example, the NFIB gene^49^ is not only specific to CGE neurons but also highly expressed and hypomethylated in PV-expressing ChCs but not BCs (Figure 6D). The same expression pattern of NFIB was found in a comparison of mouse ChC-BC.^48^ These findings provide cogent evidence that the conserved major cell types of human and mouse^38^ also have shared basic rules of TF regulation. The same TF gene may perform multiple roles in different cell-type lineages.

Previous studies, including ours, have found that discrete genomic regions with reduced mCG (hypomethylated DMRs) mark active regulatory elements.^35,50–52^ We expected that TF binding motifs would be enriched in hypomethylated DMRs for cell types in which the TF gene is actively expressed and has low gene-body mCH. However, we identified several TFs with an opposite pattern: their binding motif was enriched in the hypomethylated DMRs of the alternative lineage showing low TF expression and high gene body mCH. For example, the motifs of NR2F1 and PBX1 were enriched in the hypomethylated DMRs of ChCs, but both TFs were actively expressed in BCs and not ChCs (Figure 6D). Similarly, the PKNOX2 motif was enriched in hypomethylated DMRs of VIP cells, yet *PKNOX2* is preferentially expressed in NDNF neurons (Figure 6E). These data suggest that certain TFs can preferentially bind to hypermethylated regions (i.e., hypomethylated regions in the alternative lineage). This non-classical preference for methylated binding sites has been extensively demonstrated in *in vitro* studies.^45,53^ In particular, Yin et al.^45^ used an *in vitro* assay to bind each recombinant TF protein to a pool of synthetic DNA (methyl-SELEX). They identified hundreds of TFs whose binding is inhibited (MethylMinus) or promoted (MethylPlus) by the presence of methylated CpG sites in their binding motifs. We analyzed the *in vivo* binding of MethylPlus TFs to hypermethylated DNA by analyzing chromatin accessibility measured by the snmCAT-seq NOMe-seq profile (Figure 6F) as well as snATAC-seq (Figure S6I). We quantified the average chromatin accessibility at TF binding motifs that are lowly methylated (overlapping with hypomethylated DMRs) or highly methylated (overlapping with hypermethylated DMRs) (Figures 6F and S6I) and used the difference in chromatin accessibility to determine the *in vivo* sensitivity of each TF to cytosine methylation. Using both chromatin accessibility assays (NOMe-seq and ATAC-seq), we found a general agreement between our *in vivo* approach and the *in vitro* methyl-SELEX results with MethylMinus TFs showing enrichment in the upper part of Figures 6F and S6I (e.g., ETV1 in Figure 6J), which showed a greater difference in chromatin accessibility between lowly and highly methylated TF motifs (Figures 6H and S6K). Consistently, MethylPlus TFs are strongly depleted in the upper part of Figures 6F and S6I (Figures 6G and S6J). Therefore, our joint analysis of mC and chromatin accessibility using snmCAT-seq provided *in vivo* evidence for the modulation of TF binding by cytosine methylation.

Lastly, we examined the correlation between chromatin accessibility and the presence of CA dinucleotide in the TF binding motifs because CA is the predominant sequence context of mCH in the human brain.^22^ Intriguingly, we found a significant enrichment of TF binding motifs containing CA dinucleotides in the lower part of Figures 6F and S6I, using either NOME-seq or ATAC-seq to quantify chromatin accessibility (Figures 6I and S6L), suggesting that the accessibility of TF binding motifs containing CA is less affected by mC. Across all TF binding motifs examined, the accessibility of motifs containing both CA and CG dinucleotides (CA+ CG+, p value = 1 × 10^−4^, e.g., ATF4, Figure 6L) or only CA (CA+ CG−, p value = 5.7 × 10^−6^, e.g., RARB, Figure 6K) shows significantly less sensitivity to mC than motifs containing CG dinucleotides only (CA− CG+) (Figure 6M). The results suggest that certain TFs may be able to bind hypermethylated regions through the interaction with mCA sites. The modulation of TF binding by mCA has not been systematically explored since previous studies have focused on the effect of mCG sites.^45,53^

### Cortical cell regulatory genomes predict developmental and adult cell types associated with neuropsychiatric diseases

The strong enrichment of disease heritability in gene-regulatory elements has allowed the prediction of disease-associated cell types using epigenomic signatures,^54^ including neuropsychiatric disorders.^36^ By reconstructing mC and open chromatin maps from single-cell profiles, we used LD (linkage disequilibrium) score regression partitioned heritability to infer the relevant cell types for a set of neuropsychiatric traits using DMRs and ATAC-seq peaks (Tables S9 and S10).^54^ To capture regulatory elements active during early development that may be implicated in psychiatric disease, we further included lowly methylated regions identified from bulk fetal (PCW 19) human cortex methylome^37^ and DNase-seq peaks identified from fetal brain samples.^55^ We first compared the set of DMRs in each brain cell type individually to a baseline containing DMRs identified across non-brain human tissues.^52^ Using a statistical threshold of FDR < 1 × 10^−5^, we identified 72 disease-cell-type associations across 21 cortical cell types or bulk samples for 16 neuropsychiatric traits (Figure S7A). Each association corresponds to a significant enrichment of disease heritability within the corresponding cell type’s active regulatory regions. By contrast, no association was found in DMRs identified from 18 bulk non-brain tissues (Figure S7A).^52^ This result strongly suggests that our partitioned heritability analysis has correctly identified the brain as the relevant tissue types for neuropsychiatric traits.

**Figure 7.**
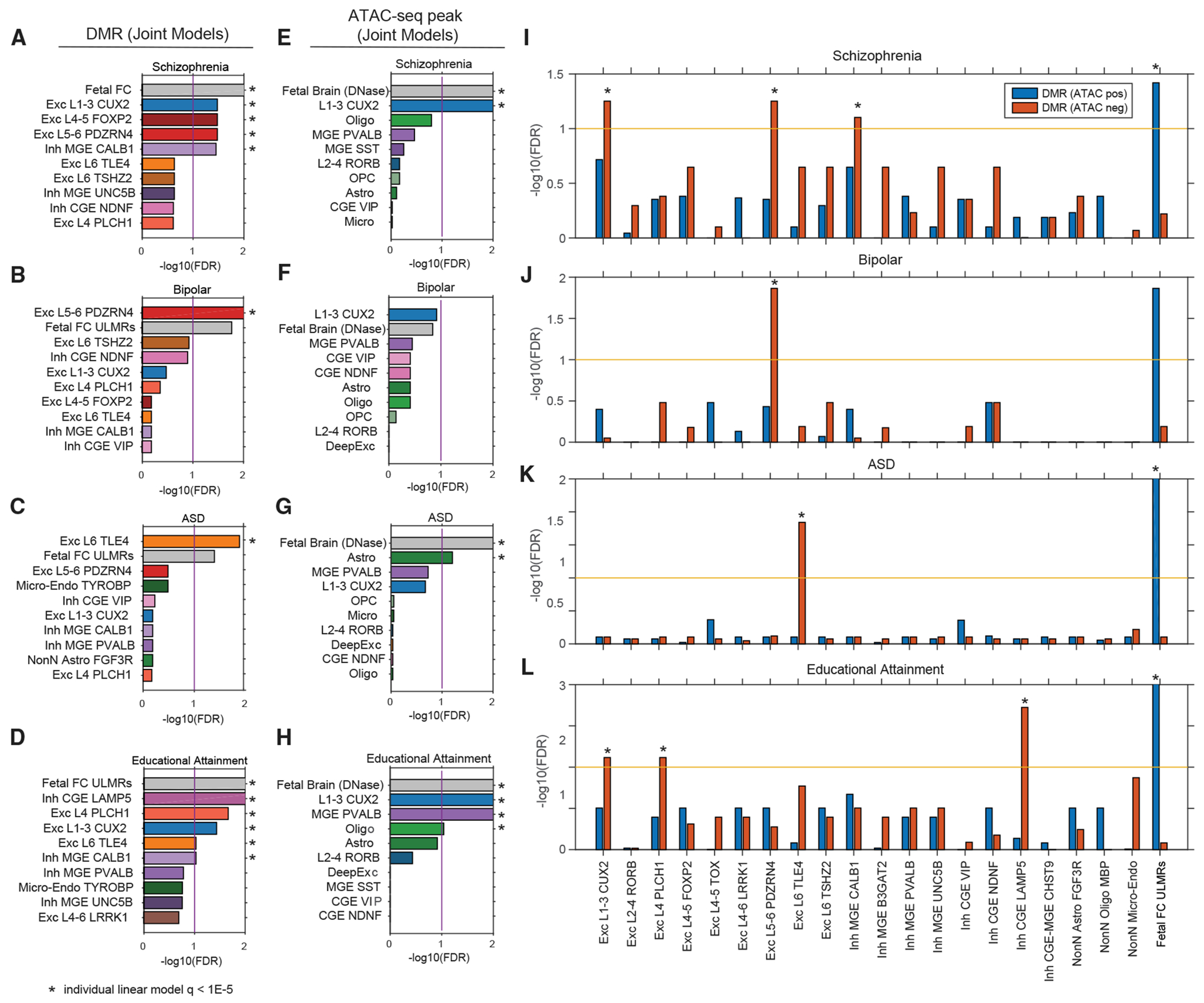
Identification of brain cell types involved in neuropsychiatric traits (A–D) Multiple regression partitioned heritability analysis using cell-type-specific DMRs for schizophrenia (A), bipolar disorders (B), autism spectrum disorder (C), or educational attainment (D). (E–H) Multiple regression partitioned heritability analysis using ATAC-seq peaks for schizophrenia (E), bipolar disorders (F), autism spectrum disorder (G), or educational attainment (H). (I–L) Multiple regression partitioned heritability analysis using DMRs stratified for the overlap with open chromatin regions. Heritability enrichment with a p value < 1E–5 compared to the baseline was indicated by asterisks.

To discern the relative enrichment of disease risk between brain cell types, we further constructed multiple regression models including all adult brain cell types and the fetal brain (Figures 7A–7D). In most cases, our partitioned heritability analyses enhanced the cell-type resolution compared to previous efforts. For example, using single-cell RNA-seq datasets, the genetic risk of schizophrenia was previously mapped to broad cortical neuronal populations, including neocortical somatosensory pyramidal cells, and cortical interneurons.^36,56^ Our analysis further identified the enrichment of schizophrenia heritability in multiple types of intratelencephalic (IT) neuron types (L1-3 CUX2, L4-5 FOXP2. and L5-6 PDZEN4), in addition to a MGE-derived inhibitory cell type (MGE CALB1) (Figure 7A). Intriguingly, the heritability of bipolar disorder was specifically enriched in a deep-layer neuron type L5-6 PDZEN4 (Figure 7B). We also found a specific enrichment of autism spectrum disorder risk in a deep-layer thalamic-projecting neuronal population L6 TLE4 (Figure 7C). By contrast, the heritability of educational attainment was broadly distributed across multiple types of neurons, including excitatory cells (L1-3 CUX2, L4 PLCH1, and L6 TLE4) and inhibitory neurons derived from both CGE (CGE LAMP5) and MGE (MGE CALB1) (Figure 7D). Consistent with the neurodevelopmental hypothesis that gene misregulation during brain development underlies certain psychiatric disorders,^57^ lowly methylated regions in fetal cortex DMRs are enriched in the heritability for schizophrenia and educational attainment (Figure S7A). However, the partitioned heritability analysis using the fetal cortex sample is likely underpowered because of the cell-type heterogeneity. To corroborate our results that were generated using LD score regression partitioned heritability, we applied RolyPoly^58^ to prioritize trait-relevant cell types using genome-wide association study (GWAS) SNP effect sizes and cell-type-specific mCG levels at DMRs (Figures S7E and S7F). The analysis using RolyPoly recapitulated a number of predictions, such as the association between schizophrenia and L5-6 PDZRN4 cells and MGE-derived inhibitory cells, bipolar disorder with L5-6 PDZRN4 cells, autism spectrum disorder with L6 TLE4 cells, and educational attainment with the L1-3 CUX2 population (Figures S7E and S7F).

We performed partitioned heritability analyses using three complementary types of molecular signatures (Figures S7B–S7D): genes with cell-type-specific expression (Figures S7B and S7D), DMRs, and open chromatin regions identified with both snATAC-seq and NOMe-seq (Figures S7B and S7C) (or DNase-seq peaks for the prenatal brain sample). To our surprise, the results obtained using DMRs (Figures 7A–7D) and ATAC-seq peaks (Figures 7E–7H) were substantially different. For example, the partition of schizophrenia heritability across DMRs identified enrichment in four adult cell types in addition to the fetal cortex (Figure 7A), whereas the analysis using open chromatin regions only found enrichment in L1-3 CUX2 cells and the fetal brain (Figure 7E). To understand this discrepancy, we stratified DMR regions into two groups (DMR [ATAC-pos] and DMR [ATAC-neg]) by their overlap with open chromatin regions. Partitioned heritability across the stratified DMR regions revealed that, in adult cells, DMR regions without open chromatin signature are more strongly enriched in heritability for the neuropsychiatric traits (Figures 7I–7L). In the fetal cortex, however, a stronger enrichment of schizophrenia and educational attainment heritability was found in DMRs associated with open chromatin.

We speculate that DMRs without open chromatin contain vestigial enhancers,^59^ which contribute to the enrichment of disease heritability. Vestigial enhancers are active regulatory elements during embryonic development but become dormant in adult tissues.^59^ However, vestigial enhancers remain lowly methylated in adult tissues and can be identified as DMRs. Thus, vestigial enhancers can be strongly enriched in the genetic risk of neuropsychiatric traits because these regions are active regulatory elements during brain development. We identified the fraction of adult brain DMRs that correspond to vestigial enhancers, i.e., overlapping with open chromatin regions in the embryonic, but not the adult, brain (Figures S7G–S7J). Consistent with our speculation, in many cases, vestigial enhancers show stronger enrichment of disease heritability (Figures S7K–S7N). In particular, the enrichment of autism spectrum disorder genetic risk in L4 PLCH1 and L6 TLE4 cells can only be identified in vestigial enhancers (Figure S7M). In summary, we found that single cell-type DMRs integrate regulatory information during brain development and in the adult brain and can be used to predict cell types involved in neuropsychiatric disorders. However, our predictions should be considered in light of important limitations. Statistical approaches such as LD score regression partitioned heritability^54^ and RolyPoly^58^ have been validated for the prioritization of trait-associated tissues, but their application to fine-grained cell types remains preliminary. In addition, experimental validation of the association between disease and cell types is challenging because of the difficulty in accurately recapitulating disease phenotypes and modeling diverse cell populations in cell cultures.^60^ Together, the investigation of disease-associated cell types is still in its infancy and will require further methodological breakthroughs in cell culture and gene-editing approaches.

## DISCUSSION

Epigenomic studies often incorporate multiple molecular profiles from the same sample to explore possible correlations between gene-regulatory elements and expression. The need for multi-omic comparison poses a challenge for single-cell analysis because most existing single-cell techniques terminally consume the cell, precluding multi-dimensional analysis. To address this challenge, we have developed a single-nucleus multi-omic assay, snmCAT-seq, to jointly profile the transcriptome, DNA methylome, and chromatin accessibility and that can be applied to either single cells or nuclei isolated from frozen human tissues. snmCAT-seq requires no physical separation of DNA and RNA and is designed to be a “single-tube” reaction for steps before bisulfite conversion to minimize material loss. snmCAT-seq is fully compatible with high-throughput single-cell methylome techniques, such as snmC-seq2,^16^ and can be readily scaled to analyze thousands of cells and/or nuclei.

The continuous development of multi-omic profiling techniques, such as scNMT-seq^14^ and snmCAT-seq, and several methods for joint RNA and chromatin accessibility profiling sci-CAR,^61^ SNARE-seq,^62^ Paired-seq,^63^ and SHARE-seq^64^ provide the opportunity to classify cell types with multiple molecular signatures. Our study developed computational methods to cross-validate clustering-based cell-type classifications using multi-modal data. Through cross-validation between matched single-cell mC and RNA profiles, we found that between 20 and 50 human cortical cell types can be identified from our moderately sized snmCAT-seq dataset (4,358 cells) with sound cluster robustness. This is consistent with the number of human frontal cortex cell types we reported in our previous (21 major types^4^) and current (20 major types and 63 subtypes) studies. Determining the optimal number of clusters for any dataset should consider statistical robustness, the need of the biological questions, and the cell-type resolution of companion data modalities. Practical factors could also impact the choice of clusters, such as the requirement of certain minimum coverage for the pseudo-bulk methylome for DMR analysis. Together, although statistical robustness is essential for any cell-type classification using clustering methods, the optimal number of clusters is, to some extent, an investigator-driven choice depending on the context of the study. Using snmCAT-seq as a “ground truth,” we determined that computational multi-modal data fusion tools perform well at the major cell-type level but show variable accuracy for the fusion of fine-grain subtypes. The computational strategies developed in this study can be applied to other types of multi-omic profiling, including methods involving physiological measurement such as Patch-seq.^65,66^

Epigenomic studies at both bulk and single-cell levels have established both mC and open chromatin as reliable markers for regulatory elements.^1^ However, the difference between the information provided by the two epigenomic marks has been less clear in the context of normal development and diseases. Our study found that DMRs contain disease-related regulatory information of both adult and embryonic tissues, with vestigial enhancers^59^ as a possible mechanism that informs developmental gene regulation. The strong enrichment of genetic risks for neuropsychiatric disorders in vestigial enhancers enabled the prediction of cellular lineages associated with diseases using DMRs for partitioned heritability analyses and identified more diverse disease-associated brain cell populations than similar analyses using open chromatin regions. The abundance of developmental information in DNA methylome suggests the possibility of studying developmental processes and gene regulation in cell lineages using methylome profiling of adult tissues, especially given the practical and ethical challenges for obtaining primary human tissues from developmental stages.

### Limitations

The transcriptome assay of snmCAT-seq was based on the Smart-seq2 method^18^ published more than 7 years ago. The incorporation of further optimized single-cell RNA approaches such as Smart-seq3^67^ may enhance the performance of transcriptome profiling for snmCAT-seq. Similar to other bisulfite sequencing-based approaches, the relatively high cost of resequencing the bisulfite-converted genome limits the number of cells that can be profiled with snmCAT-seq. However, with the continuous reduction of sequencing cost, it will become feasible to routinely profile hundreds of thousands of snmCAT-seq libraries. Although the current plate-based library preparation method of snmCAT-seq has a maximum throughput of approximately 10,000 cells per week,^16^ the molecular partitioning design of snmCAT-seq is a simple “single-tube” reaction and can be readily combined with combinatorial indexing-based methylome preparation methods such as sci-MET.^68^ In snmCAT-seq, the ratio between transcriptome and methylome reads is determined by the absolute quantity of mRNA and pre-mRNA in a single nucleus because the amount of genomic DNA is a constant, ~5 pg per nucleus in diploid human cells. Therefore, the application of snmCAT-seq to a new tissue type requires testing of the number of cycles of cDNA amplification necessary to achieve an optimized representation of transcriptome reads in the sequencing library.

Although we have successfully incorporated NOMe-seq in snmCAT-seq for the profiling of chromatin accessibility, the single-nucleus NOMe-seq profiles have moderate signal-to-noise ratio and may be better suited for identifying open chromatin regions using pseudo-bulk profiles rather than for the *de novo* clustering of single-cell using chromatin accessibility information (Figures S2H and S2K). This could be due to our use of frozen tissue providing an intrinsically lower signal-to-noise ratio than experiments using freshly harvested cells.^69^ Nevertheless, we have demonstrated that following the robust identification of cell types using the methylome and transcriptome components of snmCAT-seq, the quantitative analysis of pseudo-bulk NOMe-seq profiles has generated insights about the modulation of TF binding by methylcytosines (Figures 6F–6I), suggesting the unique applications of single-cell multi-modal datasets.

## STAR★METHODS

### RESOURCE AVAILABILITY

#### Lead contact

Further information and requests for resources and reagents should be directed to and will be fulfilled by the lead contact, Joseph R. Ecker (ecker@salk.edu).

#### Materials availability

This study did not generate new unique reagents.

#### Data and code availability

- Raw and processed data included in this study were deposited to NCBI GEO/SRA with accession number GSE140493. Methylome and transcriptomic profiles generated by snmCAT-seq from H1 and HEK293T cells can be visualized at http://neomorph.salk.edu/Human_cells_snmCT-seq.php. snmCAT-seq generated from brain tissues can be visualized at http://neomorph.salk.edu/human_frontal_cortex_ensemble.php. snRNA-seq data is available for download from the Neuroscience Multi-omics Archive (https://assets.nemoarchive.org/dat-s3creyz).
- The code for SingleCellFusion is available from https://github.com/mukamel-lab/SingleCellFusion. The code benchmarking computational integration methods are available from https://github.com/lhqing/snmCAT-seq_integration. The code reproducing the over- and under-splitting analysis are available from https://github.com/FangmingXie/mctseq_over_under_splitting/blob/master/over-under-splitting-analysis.ipynb.
- A detailed bench protocol for snmCAT-seq and future updates to the method can be found at https://www.protocols.io/view/snmcat-v1-bwubpesn. Any additional information required to reanalyze the data reported in this paper is available from the lead contact upon request.

### EXPERIMENTAL MODEL AND SUBJECT DETAILS

#### Cell cultures

HEK293T cells were cultured in DMEM with 15% FBS and 1% Penicillin-Streptomycin and dissociated with 1X TrypLE. H1 human ESCs (WA01, WiCell Research Institute) were maintained in a feeder-free mTesR1 medium (StemCell Technologies, Inc.). HEK293T and H1 cells were cultured at 37°C and with 5% CO2. hESCs (passage 26) were dispersed with 1U/mL Dispase and collected for single-cell sorting or nuclei isolation. For the sorting of single H1 and HEK293T cells, equal amounts of H1 and HEK293T cells were mixed and stained with anti-TRA-1-60 (Biolegend, Cat#330610) antibody.

#### Human brain tissues

Postmortem human brain biospecimens GUID: NDARKD326LNK and NDARKJ183CYT were obtained from NIH NeuroBioBank at the University of Miami Brain Endowment Bank. Postmortem human brain biospecimens UMB4540, UMB5577 and UMB5580 were obtained from NIH NeuroBioBank at the University of Maryland Brain and Tissue Bank. All tissue donors provided consent in accordance with the policies of the NIH NeuroBioBank. Published snmC-seq was generated from frontal cortex (medial frontal gyrus) tissue obtained from a 25-year-old Caucasian male (UMB4540, labeled as M_25yr_1 in this study) with a postmortem interval (PMI) = 23 h. The snATAC-seq dataset was generated from specimen UMB4540. Additional snmC-seq data was generated in frontal cortex (superior frontal gyrus, Brodmann area 10) tissues obtained from a 58-year-old Caucasian male (GUID: NDARKD326LNK, labeled as M_58yr in this study) with a postmortem interval (PMI) = 23.4 h. snmC-seq2 data was generated from frontal cortex (Brodmann area 10) tissue from a 25-year-old Caucasian male (GUID: NDARKJ183CYT, labeled as M_25yr_2 in this study) with a PMI = 20.8 h. snmCAT-seq and sn-m3C-seq data were generated from a 21-year-old Caucasian male (UMB5577, labeled as M_21yr in this study) with a PMI = 19 h, and a 29-year-old Caucasian male (UMB5580, labeled as M_29yr in this study) with a PMI = 8 h. The samples were taken from unaffected control subjects who died from accidental causes. The snRNA-seq dataset was generated from postmortem brain specimen H18.30.002 from the Allen Institute for Brain Science. The frontal cortex (BA44-45, 46) from this donor was used for the generation of single nucleus RNA-seq data. The donor was a 50 year old male with a PMI = 12 h.

### METHOD DETAILS

#### Nuclei isolation from cultured cells for snmCAT-seq

Cell pellets containing 1 million cells were resuspended in 600 μl NIBT [250 mM Sucrose, 10 mM Tris-Cl pH = 8, 25 mM KCl, 5mM MgCl_2_, 0.1% Triton X-100, 1mM DTT, 1:100 Proteinase inhibitor (Sigma-Aldrich P8340), 1:1000 SUPERaseIn RNase Inhibitor (ThermoFisher Scientific AM2694), 1:1000 RNaseOUT RNase Inhibitor (ThermoFisher Scientific 10777019)]. The lysate was transferred to a pre-chilled 2 mL Dounce homogenizer (Sigma-Aldrich D8938) and Dounced using loose and tight pestles for 20 times each. The lysate was then mixed with 400 μl of 50% Iodixanol (Sigma-Aldrich D1556) and gently pipetted on top of 500 μl 25% Iodixanol cushion. Nuclei were pelleted by centrifugation at 10,000 x g at 4°C for 20 min using a swing rotor. The pellet was resuspended in 2 mL of DPBS supplemented with 1:1000 SUPERaseIn RNase Inhibitor and 1:1000 RNaseOUT RNase Inhibitor. Hoechst 33342 was added to the sample to a final concentration of 1.25 nM and incubated on ice for 5 min for nuclei staining. Nuclei were pelleted by 1,000 x g at 4°C for 10 min and resuspended in 1 mL of DPBS supplemented with RNase inhibitors.

#### Nuclei isolation from human brain tissues and GpC methyltransferase treatment for snmCAT-seq

Brain tissue samples were ground in liquid nitrogen with cold mortar and pestle, and then aliquoted and store at −80°C. Approximately 100mg of ground tissue was resuspended in 3 mL NIBT (250 mM Sucrose, 10 mM Tris-Cl pH = 8, 25 mM KCl, 5mM MgCl_2_, 0.2% IGEPAL CA-630, 1mM DTT, 1:100 Proteinase inhibitor (Sigma-Aldrich P8340), 1:1000 SUPERaseIn RNase Inhibitor (ThermoFisher Scientific AM2694), 1:1000 RNaseOUT RNase Inhibitor (ThermoFisher Scientific 10777019)). The lysate was transferred to a pre-chilled 7 mL Dounce homogenizer (Sigma-Aldrich D9063) and Dounced using loose and tight pestles for 40 times each. The lysate was then mixed with 2 mL of 50% Iodixanol (Sigma-Aldrich D1556) to generate a nuclei suspension with 20% Iodixanol. Gently pipet 1 mL of the nuclei suspension on top of 500 μl 25% Iodixanol cushion in each of the 5 freshly prepared 2ml microcentrifuge tubes. Nuclei were pelleted by centrifugation at 10,000 x g at 4°C for 20 min using a swing rotor. The pellet was resuspended in 1ml of DPBS supplemented with 1:1000 SUPERaseIn RNase Inhibitor and 1:1000 RNaseOUT RNase Inhibitor. A 10 μl aliquot of the suspension was taken for nuclei counting using a Biorad TC20 Automated Cell Counter. One million nuclei aliquots were pelleted by 1,000 x g at 4°C for 10 min and resuspended in 200 ml of GpC methyltransferase M.CviPI (NEB M0227L) reaction containing 1X GC Reaction Buffer, 0.32 nM S-Adenoslylmethionime, 80U 4U/μl M.CviPI, 1:100 SUPERaseIn RNase Inhibitor and 1:100 RNaseOUT RNase Inhibitor and incubated at 37°C for 8 min. The reaction was stopped by adding 800 μl of ice-cold DPBS with 1:1000 RNase inhibitors and mixing. Hoechst 33342 was added to the sample to a final concentration of 1.25 nM and incubated on ice for 5 min for nuclei staining. Nuclei were pelleted by 1,000 x g at 4°C for 10 min, resuspended in 900 ml of DPBS supplemented with 1:1000 RNase inhibitors and 100 μl of 50mg/mL Ultrapure™ BSA (Ambion AM2618) and incubated on ice for 5 min for blocking. Neuronal nuclei were labeled by adding 1 μl of AlexaFluor488-conjugated anti-NeuN antibody (clone A60, MilliporeSigma MAB377XMI) for 20 min.

#### Reverse transcription for snmCAT-seq

Single cells or single nuclei were sorted into 384-well PCR plates (ThermoFisher 4483285) containing 1 μl snmCAT-seq reverse transcription reaction per well. The snmCAT-seq reverse transcription reaction contained 1X Superscript II First-Strand Buffer, 5mM DTT, 0.1% Triton X-100, 2.5 mM MgCl_2_, 500 μM each of 5’-methyl-dCTP (NEB N0356S), dATP, dTTP and dGTP, 1.2 μM dT30VN_4 oligo-dT primer (5’-AAGCAGUGGUAUCAACGCAGAGUACUTTTTTUTTTTTUTTTTTUTTTTTUTTTTTVN-3’ was used the cultured cell snmCAT-seq experiments; 5’-/5SpC3/AAGCAGUGGUAUCAACGCAGAGUACUTTTTTUTTTTTUTTTTTUTTTTTUTTTTTVN-3’ was used for human brain snmCAT-seq experiments), 2.4 μM TSO_3 template switching oligo (5’-/5SpC3/AAGCAGUGGUAUCAACGCAGAGUGAAUrGrG+G-3’), 1U RNaseOUT RNase inhibitor, 0.5 U SUPERaseIn RNase inhibitor, 10U Superscript II Reverse Transcriptase (ThermoFisher 18064-071). For snmCAT-seq performed with nuclei samples, the reaction further included 2 μM N6_2 random primer (5’-/5SpC3/AAGCAGUGGUAUCAACGCAGAGUACNNNNNN-3’). After sorting, the PCR plates were vortexed and centrifuged at 2000 x g. The plates were placed in a thermocycler and incubated using the following program: 25°C for 5 min, 42°C for 90min, 70°C 15min followed by 4°C.

#### cDNA amplification for snmCAT-seq

3 μl of cDNA amplification mix was added into each snmCAT-seq reverse transcription reaction. Each cDNA amplification reaction containing 1X KAPA 2G Buffer A, 600 nM ISPCR23_2 PCR primer (5’-/5SpC3/AAGCAGUGGUAUCAACGCAGAGU-3’), 0.08U KAPA2G Robust HotStart DNA Polymerase (5 U/μL, Roche KK5517). PCR reactions were performed using a thermocycler with the following conditions: 95°C 3min -> [95°C 15 s -> 60°C 30 s -> 72°C 2min] -> 72°C 5min -> 4°C. The cycling steps were repeated for 12 cycles for snmCAT-seq using H1 or HEK293 whole cells, 15 cycles for snmCAT-seq using H1 or HEK293 nuclei and 14 cycles for snmCAT-seq using human brain tissue nuclei.

#### Digestion of unincorporated DNA oligos for snmCAT-seq

For snmCAT-seq using H1 and HEK293 cells, 1 μl uracil cleavage mix was added into cDNA amplification reaction. Each 1 μl uracil cleavage mix contains 0.25 μl Uracil DNA Glycosylase (Enzymatics G5010) and 0.25 μl Endonuclease VIII (Enzymatics Y9080) and 0.5 μl Elution Buffer (QIAGEN 19086). Unincorporated DNA oligos were digested at 37°C for 30 min using a thermocycler. We have found that Endonuclease VIII is dispensable for the digestion of unincorporated DNA oligos since the alkaline condition during the desulfonation step of bisulfite conversion can effectively cleave abasic sites created by Uracil DNA Glycosylase.^72^ Therefore for snmCAT-seq using human brain tissues, each cDNA amplification reaction was treated with 1μl uracil cleavage mix containing 0.5 μl Uracil DNA Glycosylase (Enzymatics G5010-1140) and 0.5 μl Elution Buffer (QIAGEN 19086).

#### Bisulfite conversion and library preparation

Detailed methods for bisulfite conversion and library preparation are previously described for snmC-seq2.^4,16^ The following modifications were made to accommodate the increased reaction volume of snmCAT-seq: Following the digestion of unused DNA oligos, 25 μl instead of 15 μl of CT conversion reagent was added to each well of a 384-well plate. 90 μl instead of 80 μl M-binding buffer was added to each well of 384-well DNA binding plates. snmCAT-seq libraries performed using whole H1 or HEK293 cells were generated using the snmC-seq method as described in Luo et al., 2017.^4^ The rest of the snmCAT-seq libraries were generated using the snmC-seq2 method as described in Luo et al., 2018.^16^ The snmCAT-seq libraries generated from H1 and HEK293 cells were sequenced using an Illumina HiSeq 4000 instrument with 150 bp paired-end reads. The snmCAT-seq libraries generated from human brain specimens were sequenced using an Illumina Novaseq 6000 instrument with S4 flowcells and 150 bp paired-end mode.

### QUANTIFICATION AND STATISTICAL ANALYSIS

#### The mapping pipeline for snmC-seq, snmC-seq2, and snmCAT-seq

We implemented a versatile mapping pipeline (http://cemba-data.readthedocs.io/) for all the methylome based technologies developed by our group.^4,16^ The main steps of this pipeline include: 1) Demultiplexing FASTQ files into single-cell; 2) Reads level QC; 3) Mapping; 4) BAM file processing and QC; 5) final molecular profile generation.

For snmC-seq and snmC-seq2, the details of the five steps are described previously.^4,16^ For snmCAT-seq, steps 1 and 2 are identical as snmC-seq2, steps 3 to 5 are split into “a” for methylome and “b” for transcriptome as following:

Step 3a (methylome). To map methylome reads, reads from step 2 were mapped onto the human hg19 genome using Bismark^70^ with the same setting as snmC-seq2.

Step 3b (transcriptome). To map transcriptome reads, reads from step 2 were mapped to GENCODE human v28 indexed hg19 genome using STAR 2.7.2b^71^ with the following parameters: -*alignEndsType EndToEnd-outSAMstrandField intronMotif–outSAM-type BAM Unsorted–outSAMunmapped Within–outSAMattributes NH HI AS NM MD–sjdbOverhang 100–outFilterType BySJout–outFilterMultimapNmax 20–alignSJoverhangMin 8–alignSJDBoverhangMin 1–outFilterMismatchNmax 999–outFilterMismatchNoverLmax 0.04–alignIntronMin 20–alignIntronMax 1000000–alignMatesGapMax 1000000–outSAMattrRGline ID:4 PL:Illumina*.

Step 4a (methylome). PCR duplicates were removed from mapped reads using Picard MarkDuplicates. The non-redundant reads were then filtered by MAPQ > 10. To select genomic reads from the filtered BAM, we used the “XM-tag” generated by Bismark to calculate reads methylation level and keep reads with mCH ratio < 0.5 and the number of cytosines ≥ 3.

Step 4b (transcriptome), the STAR mapped reads were first filtered by MAPQ > 10. To select RNA reads from the filtered BAM, we used the “MD” tag to calculate reads methylation level and keep reads with mCH ratio > 0.9 and the number of cytosines ≥ 3. The stringency of read partitioning was determined by applying the criteria for identifying snmCAT-seq transcriptome reads to snmC-seq2 data (SRR6911760, SRR6911772, SRR6911776),^16^ which contains no transcriptomic reads. Similarly, the criteria for identifying snmCAT-seq methylome reads were applied to Smart-seq data (SRR944317, SRR944318, SRR944319, SRR944320),^18^ which contains no methylome reads.

Step 5a (methylome), Tab-delimited (ALLC) files containing methylation level for every cytosine position was generated using methylpy *call_methylated_sites* function^52^ on the BAM file from the step 4a. For snmCAT-seq, an additional base was added before the cytosine in the context column of the ALLC file using the parameter “–num_upstr_bases 1,” to distinguish GpC sites from HpC sites for the NOMe-seq modality.

Step 5b (transcriptome), BAM file from step 4b were counted across gene annotations using featureCount 1.6.4^73^ with the default parameters. Gene expression was quantified using either only exonic reads with “-t exon” or both exonic and intronic reads with “-t gene.”

#### Methylome feature generation

After allc files were generated, the methylcytosine (*mc*) and total cytosine basecalls (*cov*) were summed up for each 100kb bin across the hg19 genome. For snmC-seq and snmC-seq2, cytosine and methylcytosine basecalls in CH (H = A, T, C) and CG context were counted separately. For snmCAT-seq, the HCH context was counted for CH methylation and HCG is counted for CG methylation. The GCY (Y = T, C) context was counted as the chromatin accessibility signal (NOMe-seq in snmCAT-seq) and the HCY context was counted as the endogenous mCH background. In addition to the 100kb feature set, we also counted gene body methylation levels using gene annotation from GENCODE v28. The 100kb feature set was used in methylation-based clustering analysis and data integration; the gene body feature set was used in methyl-marker identification, cluster annotation and data fusion between methylome and transcriptome.

#### Preprocessing of snmC-seq and snmC-seq2 data for clustering analyses

##### Cell filtering

We filtered the cells based on these main mapping metrics: 1) mCCC rate < 0.03. mCCC rate reliably estimates the upper bound of bisulfite non-conversion rate,^4^ 2) overall mCG rate > 0.5, 3) overall mCH rate < 0.2, 4) total final reads > 500,000, 5) Bismark mapping rate > 0.5. Other metrics such as genome coverage, PCR duplicates rate, index ratio were also generated and evaluated during filtering. However, after removing outliers with the main metrics 1-5, few additional outliers can be found.

##### Feature filtering

100kb genomic bin features were filtered by removing bins with mean total cytosine base calls < 300 or > 3000. Regions that overlap with the ENCODE blacklist^74^ were also removed from further analysis.

##### Computation and normalization of the methylation rate

For CG and CH methylation, the computation of methylation rate from the methylcytosine and total cytosine matrices contains two steps: 1) prior estimation for the beta-binomial distribution and 2) posterior rate calculation and normalization per cell.

Step 1, for each cell we calculated the sample mean, *m*, and variance, *v*, of the raw mc rate *(mc / cov)* for each sequence context (CG, CH). The shape parameters (*α*, *β*) of the beta distribution were then estimated using the method of moments:

α=m(m(1−m)/v−1)

β=(1−m)(m(1−m)/v−1)

This approach used different priors for different methylation types for each cell, and used weaker prior to cells with more information (higher raw variance).

Step 2, We then calculated the posterior: $\hat{mc}=\frac{\alpha +mc}{\alpha +\beta +cov}.,$We normalized this rate by the cell’s global mean methylation, m=α/(α+β). Thus, all the posterior $\hat{mc}$ with 0 *cov* will be constant 1 after normalization. The resulting normalized *mc* rate matrix contains no NA (not available) value and features with less *cov* tend to have a mean value close to 1.

##### Selection of highly variable features

Highly variable methylation features were selected based on a modified approach using the scanpy package *scanpy.pp.highly_variable_genes* function.^75^ In brief, the *scanpy.pp.highly_variable_genes* function normalized the dispersion of a gene by scaling with the mean and standard deviation of the dispersions for genes falling into a given bin for mean expression of genes. In our modified approach, we reasoned that both the mean methylation level and the mean *cov* of a feature (100kb bin or gene) can impact *mc* rate dispersion. We grouped features that fall into a combined bin of mean and *cov*, and then normalized the dispersion within each mean-*cov* group. After dispersion normalization, we selected the top 3000 features based on normalized dispersion for clustering analysis.

##### Dimension reduction and combination of different mC types

For each selected feature, *mc* rates were scaled to unit variance and zero mean. PCA was then performed on the scaled *mc* rate matrix. The number of significant PCs was selected by inspecting the variance ratio of each PC using the elbow method. The CH and CG PCs were then concatenated together for further analysis in clustering and manifold learning.

#### Preprocessing of snmCAT-seq data for clustering analysis

##### Methylome preprocessing

The methylome modality preprocessing is similar to snmC-seq2 with one major modification: non-CG methylation is quantified using the HCH context; CG methylation is quantified using the HCG context. Chromosome 100kb bin features with mean total cytosine base calls between 250 and 2500 were included in downstream analyses.

##### Transcriptome preprocessing

The whole gene RNA read count matrix is used for snmCAT-seq transcriptome analysis. Cells are filtered by the number of genes expressed > 200 and genes are filtered by the number of cells expressed > 10. The count matrix X is then normalized per cell and transformed by ln(X + 1). After log transformation, we use the *scanpy.pp.highly_variable_genes* to select the top 3000 genes based on normalized dispersion, using a process similar to the selection of highly variable methylation features. The selected feature matrix is scaled to unit variance and zero mean per feature followed by PCA calculation.

##### Chromatin accessibility (NOMe-seq) preprocessing

For clustering analysis, cytosine methylation in the GCY context (GmCY) is counted as the open chromatin signal from NOMe-seq. For each 5 kb bin, we modeled its GmCY basecall in a single cell using a binomial distribution Bi(*cov*, *global*), where *cov* represents the total GCY basecall of the bin in the cell, and *global* represents the global GmCY level of the cell. We then computed the probability of observing equal or greater GmCY basecall than observed as the survival function of the binomial distribution. The bins with this probability smaller than 0.05 were marked as 1, and otherwise 0, by which we generated a *#cell×#bin* binarized matrix as the open chromatin signals. Latent semantic analysis with log term frequency was used to compute the embedding. Specifically, we selected the bins that are open in > 10 cells, then computed the column sum of the matrix and kept only the bins with Z-scored column sum < 2. The filtered matrix *A* was row normalized to *B* by dividing the row sum, and $C_{ij}=log\left(B_{ij}+1\right)\times log\left(1+\frac{\#cells}{\sum_{i\prime =1}^{\#cells}A_{i\prime j}}\right)$ was used for dimension reduction by singular value decomposition. We used the first 15 dimensions of the left singular vector matrix as the input of UMAP for visualization.

#### General strategies for clustering and manifold learning

##### Consensus clustering on concatenated PCs

We used a consensus clustering approach based on multiple Leiden-clustering^76^ over K-Nearest Neighbor (KNN) graph to account for the randomness of the Leiden clustering algorithms. After selecting dominant PCs from PCA in all available modalities of different technologies (mCH, mCG for snmC-seq and snmC-seq2; mCH, mCG, RNA, NOMe-seq for snmCAT-seq, etc.), we concatenated the PCs together to construct KNN graph using scanpy.pp.neighbors. Given fixed resolution parameters, we repeated the Leiden clustering 200 times on the KNN graph with different random starts and combined these cluster assignments as a new feature matrix, where each single Leiden result is a feature. We then used the outlier-aware DBSCAN algorithm from the scikit-learn package to perform consensus clustering over the Leiden feature matrix using the hamming distance. Different epsilon parameters of DBSCAN are traversed to generate consensus cluster versions with the number of clusters that range from minimum to the maximum number of clusters observed in the 200x Leiden runs. Each version contains a few outliers that usually fall into three categories: 1. Cells located between two clusters that have gradient differences instead of clear borders, e.g., L2-3 IT to L4 IT; 2. Cells with a low number of reads that potentially lack information in important features to determine the exact cluster. 3. Cells with a high number of reads that are potential doublets. The number of type 1 and 2 outliers depends on the resolution parameter and is discussed in the choice of the resolution parameter section, the type 3 outliers are very rare after cell filtering. The final consensus cluster version is then determined by the supervised model evaluation.

##### Supervised model evaluation on the clustering assignment

For each consensus clustering version, we performed a Recursive Feature Elimination with Cross-Validation (RFECV)^77^ process from the scikit-learn package to evaluate clustering reproducibility. We first removed the outliers from this process, then we held out 10% of the cells as the final testing dataset. For the remaining 90% of the cells, we used tenfold cross-validation to train a multiclass prediction model using the input PCs as features and *sklearn.metrics.balanced_accuracy_score*^78^ as an evaluation score. The multiclass prediction model is based on BalancedRandomForestClassifier from the imblearn package that accounts for imbalanced classification problems.^79^ After training, we used the 10% testing dataset to test the model performance using the balanced_accuracy_score score. We kept the best model and corresponding clustering assignments as the final clustering version. Finally, we used this prediction model to predict outliers’ cluster assignments, we rescued the outlier with prediction probability > 0.5, otherwise labeling them as outliers.

##### Choice of resolution parameter

Choosing the resolution parameter of the Leiden algorithm is critical for determining the final number of clusters. We selected the resolution parameter by three criteria: 1. The portion of outliers < 0.05 in the final consensus clustering version. 2. The final prediction model performance > 0.95. 3. The average cell per cluster ≥ 30, which controls the cluster size in order to reach the minimum coverage required for further epigenome analysis such as DMR calling. All three criteria prevent the over-splitting of clusters thus we selected the maximum resolution parameter under meeting the criteria using grid search in each specific clustering analysis below.

##### Cluster marker gene identification and cluster trimming

After clustering, we used a one-versus-rest strategy to calculate methylation (methyl-marker) and RNA (rna-marker, for snmCAT-seq only) marker genes for each cluster. We used all the protein-coding and long non-coding RNA genes with evidence level 1 or 2 from gencode v28. For the rna-marker, we used the *scanpy.tl.rank_genes_group* function with the Wilcoxon test and Benjamini-Hochberg multi-test correction, and filtered the resulting marker gene by adjusted P value < 0.01 and log2(fold-change) > 1, we also used AUROC score as a measure of marker gene’s predictability of corresponding cluster, and filtered genes by AUROC > 0.8. For the methyl-marker, we used the normalized gene body mCH rate matrix to calculate markers for neuronal clusters and the normalized gene body mCG rate matrix for non-neuronal clusters, and we modified the original Wilcoxon test function to used a reverse score to select genes that have significant decrease (hypomethylation). Marker gene is chosen based on adjusted P value < 0.01, delta methylation level change < −0.3 (hypo-methylation), AUROC > 0.8. The delta methylation level is calculated as the normalized methylation rate change between the cluster and the mean value of the rest clusters. For the ensemble methylome clustering, if a cluster with the number of methyl-markers < 10 is detected, the cluster with the minimum total marker genes are merged to the closest clusters based on cluster centroids euclidean distance in the PC space, then the marker identification process is repeated until all clusters found enough marker genes.

##### Manifold learning

The T-SNE and UMAP embedding are run on the PC matrix the same as the clustering input using the scanpy package.

#### Identification of open chromatin regions using snmCAT-seq GCY methylation profiles

Methylated GCY sites were identified using a Hidden Markov Model (HMM) method gNOMePeaks,^30^ with the methylation level of GCY sites modeled using binomial distribution. The accessibility state of each GCY site was modeled with a three-state HMM model with state 3 indicating accessible chromatin. To tune the HMMmodel for the different background mCH levels between neuronal and non-neuronal cell types, we skipped the expectation-maximization (EM) algorithm for estimating the p parameter of binomial distribution. Instead, for neuronal cell types, p parameters were specified as 0.1, 0.2 and 0.5 for states 1-3, respectively; for non-neuronal cell types, p parameters were specified as 0.1, 0.25 and 0.4 for states 1-3, respectively. The density of methylated GCY sites across the genome was modeled using Poisson distribution by MACS2^80^ and regions with a significant enrichment of methylated GCY sites were identified with MACS2 *callpeak* with a p value < 0.01. Peaks with q-value < 0.01 were selected for downstream analyses.

#### snATAC-seq data generation

Combinatorial barcoding single nucleus ATAC-seq was performed as described previously in Fang et al.^81^ Isolated brain nuclei were pelleted with a swinging bucket centrifuge (500 x g, 5 min, 4°C; 5920R, Eppendorf). Nuclei pellets were resuspended in 1 mL nuclei permeabilization buffer (5% BSA, 0.2% IGEPAL-CA630, 1mM DTT and cOmplete™, EDTA-free protease inhibitor cocktail (Roche) in PBS) and pelleted again (500 x g, 5 min, 4°C; 5920R, Eppendorf). Nuclei were resuspended in 500 μL high salt tagmentation buffer (36.3 mM Tris-acetate (pH = 7.8), 72.6 mM potassium-acetate, 11 mM Mg-acetate, 17.6% DMF) and counted using a hemocytometer. Concentration was adjusted to 4500 nuclei/9 μl, and 4,500 nuclei were dispensed into each well of a 96-well plate. For tagmentation, 1 μL barcoded Tn5 transposomes^81^ added using a BenchSmart 96 (Mettler Toledo), mixed five times and incubated for 60 min at 37°C with shaking (500 rpm). To inhibit the Tn5 reaction, 10 μL of 40 mM EDTA was added to each well with a BenchSmart 96 (Mettler Toledo) and the plate was incubated at 37°C for 15 min with shaking (500 rpm). Next, 20 μL 2 x sort buffer (2% BSA, 2 mM EDTA in PBS) was added using a BenchSmart 96 (Mettler Toledo). All wells were combined into a FACS tube and stained with 3 μM Draq7 (Cell Signaling). Using a SH800 Fluorescence-activated cell sorter (Sony), 40 nuclei were sorted per well into eight 96-well plates (total of 768 wells) containing 10.5 μL EB (25 pmol primer i7, 25 pmol primer i5, 200 ng BSA (Sigma). Preparation of sort plates and all downstream pipetting steps were performed on a Biomek i7 Automated Workstation (Beckman Coulter). After the addition of 1 μL 0.2% SDS, samples were incubated at 55°C for 7 min with shaking (500 rpm). 1 μL 12.5% Triton-X was added to each well to quench the SDS. Next, 12.5 μL NEBNext High-Fidelity 2 × PCR Master Mix (NEB) were added and samples were PCR-amplified (72°C 5 min, 98°C 30 s, (98°C 10 s, 63°C 30 s, 72°C 60 s) × 12 cycles, held at 12°C). After PCR, all wells were combined. Libraries were purified according to the MinElute PCR Purification Kit manual (QIAGEN) using a vacuum manifold (QIAvac 24 plus, QIAGEN) and size selection was performed with SPRI beads (Beckman Coulter, 0.55x and 1.5x). Libraries were purified one more time with SPRI beads (Beckman Coulter, 1.5x). Libraries were quantified using a Qubit fluorometer (Life technologies) and the nucleosomal pattern was verified using a Tapestation (High Sensitivity D1000, Agilent). The library was sequenced on a HiSeq2500 sequencer (Illumina) using custom sequencing primers, 25% spike-in library and following read

$$lengths:50+43+37+50{(Read1+Index1+Index1+Index2+Read2).}^{5}$$

#### snATAC-seq data processing

Using a custom python script, we first demulticomplexed FASTQ files by integrating the cell barcode (concatenate reads pair in I1.fastq and I2.fastq) into the read name (R1.fastq and R2 fastq) in the following format: “@”+”barcode”+””:+”original_read_name.” Demulticomplexed reads were aligned to the corresponding reference genome (hg19) using bwa (0.7.13-r1126)^82^ in pair-end mode with default parameter settings. Alignments were then sorted based on the read name using samtools (v1.9).^83^ Pair-end reads were converted into fragments and only those that are 1) properly paired (according to SMA flag value); 2) uniquely mapped (MAPQ > 30); 3) with length less than 1000bp were kept. Since fragments were sorted by barcode (integrated into the read name), fragments belonging to the same cell (or barcode) were automatically grouped together which allowed for removing PCR duplicates for each cell separately. Using the remaining fragments, a snap-format (Single-Nucleus Accessibility Profiles) file was generated. snap file is hierarchically structured hdf5 file that contains the following sessions: header (HD), cell-by-bin matrix (BM), cell-by-peak matrix (PM), cell-by-gene matrix (GM), barcode (BD) and fragment (FM). HD session contains snap-file version, date, alignment and reference genome information. BD session contains all unique barcodes and corresponding metadata. BM session contains cell-by-bin matrices of different resolutions (or bin sizes). PM session contains a cell-by-peak count matrix. PM session contains a cell-by-gene count matrix. FM session contains all usable fragments for each cell. Fragments are indexed for fast search. A detailed documentation of snap file can be found here: https://github.com/r3fang/SnapATAC/wiki/FAQs#whatissnap. After generating the SNAP file, we filtered cell barcodes based on the following criteria 1) Total Sequencing Fragments (> 1,000); 2) Mapping Ratio (> 0.8); 3) Properly Paired Ratio (> 0.9); 4) Duplicate Ratio (< 0.5); 5) Mitochondrial Ratio (< 0.1).^81^

#### Clustering analysis of snATAC-seq data

We used the snapATAC package for the clustering analysis of snATAC-seq data, the detail steps were described in.^81^ Briefly, we used the binarized cell-by-bin matrix of the whole genome 5kb non-overlapping bins as input (1 means open, 0 means close or missing data). We first determined the coverage of each bin and converted the coverage distribution to log-normal distribution and converted the bin coverage to z-score. Bins with extremely high (zscore > 1.5) or low coverage (zscore < −1.5), or overlap with ENCODE blacklist^74^ are removed. We then converted the cell-by-bin matrix into a cell-by-cell similarity matrix by calculating the Jaccard index between cells. To normalize the cell coverage impact on the Jaccard index, we used the observed over expected (OVE) method from snapATAC, which calculates the residual of the linear regression model between the expected Jaccard matrix given cell coverage and the overserved matrix. We then performed PCA on a standardized residual matrix and used the top 25 PCs for Leiden clustering (resolution = 1) and UMAP visualization.

#### Open chromatin peak calling using snATAC-seq data

Open chromatin peaks were identified using snATAC-seq reads combined for each cell type using MACS *callpeak* with the following parameters -f BED-nomodel-shift 37-ext 73-pvalue 1e-2. Peaks with q-value < 0.01 were further selected for downstream analyses.

#### snRNA-seq data generation

Nuclei were isolated from human postmortem brain tissues and sorted based on NeuN fluorescence as previously described.^38^ Each sample contained approximately 80% NeuN-positive and 20% NeuN-negative nuclei. snRNA-seq data was generated using 10x Genomics v3 single-cell chemistry per the manufacturer’s protocol. RNA-seq reads were aligned with Cell Ranger v3 using the human GRCh38.p2 reference genome, and intronic and exonic mapped reads were included in gene expression quantification.

#### snRNA-seq clustering and annotation

Nuclei were included in downstream analysis if they passed the following QC thresholds: > 500 genes detected (UMI > 0) in non-neuronal nuclei or > 1000 genes detected (UMI > 0) in neuronal nuclei; and doublet score < 0.3. Cells were grouped into transcriptomic cell types using the iterative clustering procedure described in.^7^ Briefly, genes from the mitochondrial and sex chromosomes were excluded, and expression was normalized to UMI per million and log2-transformed. Nuclei were clustered using the following steps: high variance gene selection, dimensionality reduction, dimension filtering, Jaccard-Louvain or hierarchical (Ward) clustering, and cluster merging. Differential gene expression (DGE) was computed for every pair of clusters, and pairs that did not meet the DGE criteria were merged. Differentially expressed genes were defined using two criteria: 1) significant differential expression (> 2-fold; Benjamini-Hochberg false discovery rate < 0.01) using the R package limma and 2) binary expression (CPM > 1 in more than half of the cells in one cluster and < 30% of this proportion in the other cluster). We define the deScore as the sum of the −log10(false discovery rate) of all differentially expressed genes (each gene contributes to no more than 20), and pairs of clusters with deScore < 150 were merged. This process was repeated within each resulting cluster until no more child clusters met DGE or cluster size criteria (minimum of 10 cells). The entire clustering procedure was repeated 100 times using 80% of all cells sampled at random, and the frequency with which nuclei co-cluster was used to generate a final set of clusters, again subject to differential gene expression and cluster size termination criteria. Clusters were identified as outliers if more than 40% of nuclei co-expressed markers of inhibitory (GAD1, GAD2) and excitatory (SLC17A7) neurons or were NeuN+ but did not express the pan-neuronal marker SNAP25. Median values of total UMI counts and gene counts were calculated for each cluster and used to compute the median and inter-quartile range (IQR) of all cluster medians. Clusters were also identified as outliers if the cluster median QC metrics deviated by more than three times the IQRs from the median of all clusters. In total, 23,379 nuclei passed QC criteria and were split into three broad classes of cells (13,997 excitatory neurons, 7,094 inhibitory neurons, and 1,914 non-neuronal cells) based on NeuN staining and cell class marker-gene expression. A final merge step required at least 4 marker genes to be more highly expressed in each pair of clusters. The clustering pipeline is implemented in an R package publicly available at github (https://github.com/AllenInstitute/scrattch.hicat). The clustering method is provided by the run_consensus_clust function.

#### Cell line dataset analysis

##### Clustering

For snmCAT-seq dataset generated from the whole cell and nucleus of H1 and HEK293 cells (Figure S1), PCA was used for the dimension reduction of the mCG and RNA matrices. Since only two cell types (H1 and HEK293T) need to be separated, only the first 5 PCs from each matrix were selected to construct K-Nearest Neighbor (KNN) graphs (K = 25). On each KNN graph for mCG and RNA, Leiden clustering (r = 0.5) is used to determine the two clusters and tSNE was used to visualize the PCs. Clusters were annotated by examining the genome-wide methylation levels and marker gene expression. Data acquired from single cells or nuclei were then merged for each cluster for comparisons with bulk methylome and transcriptome data.

##### Comparison to bulk H1 and HEK293 methylome

The bulk HEK293 cell whole-genome bisulfite sequencing (WGBS-seq) data were downloaded from Libertini et. al. (GSM1254259).^84^ The bulk WGBS-seq data of the H1 cell was downloaded from Schultz et al. (GSE16256).^52^ Methylpy was used to call CG-DMRs between these two cell lines.^52^ DMRs were filtered by DMS (differentially methylated sites)^3^ 5 and methylation level difference^3^ 0.6.

##### Bulk H1 and HEK293 RNA data analysis

The bulk HEK293 cell RNA-seq data was downloaded from Aktas et. al. (GSE85161),^85^ the bulk H1 cell RNA-seq data was downloaded from encodeproject.org (ENCLB271KFE, generated by Roadmap Epigenome). Gene count tables and bigwig tracks were generated using human GENCODE v19 gene annotation.

#### snmCAT-seq baseline clustering

To perform clustering analysis on the human frontal cortex snmCAT-seq dataset only, we first preprocessed three modalities separately as described in the preprocessing section above. We then concatenate all the dominant PCs together to run the consensus clustering identification (resolution = 1). We annotated the clusters based on marker genes reported in the previous studies.^4,38^ We also calculated the UMAP coordinates based on concatenated PCs and PCs from every single modality separately.

#### Methylome ensemble clustering

To generate an ensemble cell type taxonomy for the human frontal cortex (Figure 4), we combine four methylome-based technologies (Figure 4A, snmCAT-seq, snmC-seq, snmC-seq2, sn-m3C-seq) in this study. Due to the high cell-type diversity, we performed a two-level iterative clustering analysis.

##### Level 1 clustering to identify major cell types

We first preprocessed the methylation matrix as described above for each technology separately to obtain the corresponding highly variable feature matrix. We then used Scanorama to integrate all cells using the union of highly variable features from all technologies, with K = 25 and default values for other parameters. After the integration, we performed PCA on the integrated matrix and used the dominant PCs for the subsequent consensus clustering analysis (resolution = 0.5) as described above. We also calculated UMAP coordinates using the ensemble PCs (Figure 4C).

##### Level 2 clustering to identify subtypes for each major cell type

After level 1 clustering, we selected cells from each major cell type and repeated all the steps from highly variable feature selection to final clustering (K = 20, resolution = 0.8) including Scanorama integration. The highly variable features selected in this step are more specific to the intracluster diversity of each major type, which helps to better separate the subtype. The subcluster UMAP coordinates are calculated from PCs in each subtype analysis (Figure 4C insets).

#### Cross-validation of cell clusters

The cross-validation analysis in Figure 2 starts with 2 cell-by-gene data matrices: one for gene-body non-CG DNA methylation (mCH) and the other for RNA expression. We first filter out low-quality cells and low-coverage genes. After removing glia and outliers in the snmCAT-seq dataset, we get 3,898 high-quality neuronal cells. By selecting genes expressed in > 1% of cells and with > 20 cytosines coverage at gene body in > 95% of cells, we get 13,637 sufficiently covered genes. Then we normalize themCH matrix by dividing the raw mCH level by the global mean mCH level of each cell; and we normalize the RNA matrix by (log_10_(TPM+1)).

The goal of cluster cross-validation is to cluster cells with one part of the features, and to validate clustering results with the other part of features. We first generate clusterings with different granularity, ranging from coarse to very fine, using DNA methylation features. Clusterings are generated by the Leiden method applied to the top 20 principal components with different settings of the resolution parameter controlling granularity. Following clustering, we randomly split cells into training and test sets. Using the training set, we estimate the cluster centroids of RNA expression. Using the test set, we calculated the mean squared error between the RNA expression profile of individual cells and that of cluster centroids. This procedure can be reversed by clustering with RNA features and evaluation with DNA methylation features.

To summarize the results, we plotted a curve of the number of clusters versus the mean squared error. To ensure robustness, clustering is repeated with five different random seeds, with each of the 5 clusters followed by 5 repetitions of 5-fold cross-validation on different random splits of training and test sets.

#### AIC and BIC metrics in the cluster cross-validation analysis

In Figure 2, Akaike information criterion (AIC) and Bayesian information criterion (BIC) are metrics to estimate (in-sample) prediction error without a test set. The general definition of the two metrics are as follows,

AIC=−2·loglik+2d

BIC=−2·loglik+(logN)d

where *loglikis* the log-likelihood of the model trained on a specific dataset, *d* is the model dimension, Nis the sample size. For both metrics, the first term evaluates the quality of fitting, whereas the second term penalizes model complexity.

In our case, we assume gene features of a single cell follows a Gaussian distribution around its cluster centroid:

$$y_{cell}=f\left(x\right)+\varepsilon =y_{centroid}+\varepsilon \;with\;\varepsilon ∼N\left(0,\sigma I\right)$$

and with *σ*being the standard deviation in the Gaussian distribution that is the same across all dimensions (genes). Combining the model with the definitions of AIC and BIC, we get

$$AIC∼\frac{1}{N}{\left(y_{cell}-y_{centroid}\right)}^{2}+2d\cdot \frac{\sigma^{2}}{N}$$

$$BIC∼\frac{1}{N}{\left(y_{cell}-y_{centroid}\right)}^{2}+\left(log\;N\right)\;d\cdot \frac{\sigma^{2}}{N}$$

where the first term is the mean squared error of the model fit, i.e., the training error, Nis the number of cells, *d* is the number of cell clusters, and *σ*^2^ is the variance of the dataset (assuming all cells are from the same cell clusters).

We applied this to 3,898 neuronal cells from the snmCAT-seq dataset. As for gene features, we include genes that have at least 1 RNA count in > 1% cells, and with at least 20 methylation coverage in 95% of cells. This leaves 13,651 genes with both DNA methylation and RNA features. The DNA methylation features are calculated as the gene body non-CG methylation level (mHCH) normalized by the global mCHC level of each cell. The RNA features are log_10_(CPM+1) normalized. Using Leiden clustering with different resolutions, we generated clusters with different granularities. As a result, we report AIC, BIC, train and test error as functions of the number of clusters. Errorbars are estimated from running the same settings repeatedly: [clustering with 10 different random seeds] x [10-time, 3-fold cross validations].

#### Quantification of over-splitting and under-splitting of cell clusters

Clustering of cell types requires a balance between over-splitting and under-splitting; this is the perennial tension between so-called lumpers and splitters as described by Darwin.^86^ Over-splitting occurs when the noise in the data, for example due to random sampling of RNA or DNA molecules, drives the separation of cells which are not distinct. Under-splitting occurs when coarse-grained clusters fail to capture a meaningful biological distinction among subpopulations. The previous section described a cross-validation method to objectively pick a good clustering granularity for a given dataset. Here, we extend this to provide more detailed metrics of the degree of potential over- or under-splitting for particular cell clusters.

Our approach proceeds from the assumption that an ideal cluster should satisfy two requirements. First, all the cells within a cluster should be similar, with no clear discrete subdivisions that would indicate under-splitting. Second, the cells in one cluster should not resemble too closely the cells in any other cluster, which would indicate over-splitting. Unfortunately, no general methods for quantifying over- and under-splitting are available.^87^ Taking advantage of the multimodal (RNA + DNA methylation) data, we defined metrics for over-splitting (S_over_) and under-splitting (S_under_), based on cross-validation analysis of the two data modalities. We have also added a supplementary tutorial (https://github.com/FangmingXie/mctseq_over_under_splitting/blob/master/over-under-splitting-analysis.ipynb) of the over- and under-splitting analysis to allow users to reproduce our results.

##### Cross-modality k-partner graph

First, we treat the two data modalities (mC and RNA) as independent measurements, as if they came from separate DNA methylation and transcriptome assays performed on independent groups of cells. We embed cells from the two modalities into the same low-dimensional space using canonical correlation analysis:^32^

$$XY^{T}\approx USV^{T},$$

where *X* and *Y* are cell-by-gene feature matrices for mC and RNA, respectively. For mC, the gene features are normalized mCH levels at the gene bodies; For RNA, the gene features are normalized RNA expression levels (log_10_(TPM+1)). *U* and *V* are cell-by-component matrices (number of components = 20). Mathematically this procedure is equivalent to a singular value decomposition of *XY*^*T*^, where *U* and *V* are orthogonal and *S* is diagonal. One can interpret *U* and *V* as the coordinates of cells from the 2 data modalities in the shared low dimensional space.

After co-embedding, we calculated cell-cell distances between cells in the two modalities and defined k-nearest neighbors between cells. If we denote all the cells in the mC modality as *I*, and all the cells in the RNA modality as *J,* the distance between a cell *i*∈*I* and a cell *j*∈*J* is given by their Euclidean distance in the shared low dimensional space:

$$d_{ij}=\sqrt{{\left(u_{i}-v_{j}\right)}^{T}\left(u_{i}-v_{j}\right)},$$

where *u_i_* and *v_j_* are the *i*’th column of *U* and the *j*’th column of *V*, respectively. We build a bipartite graph, connecting each cell’s profile in one modality with its k-nearest neighbors in the other modality. We refer to the these cross-modality neighbors as “*k-partners,*” $P_{i}^{\left(k\right)}=\{j|d_{ij}\text{ are the k smallest distances for}j\in J\}$.

##### Over-splitting score

The over-splitting score for a cluster is the fraction of the *k-partners* of cells in that cluster that are *not* from the same cluster. This metric captures the intuition that clusters should include all of the cells with a similar molecular profile, and not divide cells with similar profiles into distinct clusters. the over-splitting score is:

$$S_{over}\left(C_{i}\right)=1-\frac{1}{{\left|C_{i}\right|}^{2}}\sum_{i=1}^{\left|C_{i}\right|}\sum_{j\in P_{i}^{\left(k\right)}}I\left[C_{i}=C_{j}\right],$$

where *i,j* are indices of individual cells, *C_i_* is the cluster containing cell *i*, and |*C_i_*| to represent the cluster size, *I* is the indicator function, and $P_{i}^{\left(k\right)}$ are the k-partners of cell *i* (with *k* = |*C_i_*| = cluster size). In other words, the over-splitting score is one minus the mean fraction of a cell’s k-partners (with *k* = |*C_i_*| = cluster size) that are also from the same cluster (*C_i_*). Therefore, the over-splitting score is bounded between zero and one. *S*_*over*_ = 0 indicates no over-splitting, while larger values of *S*_*over*_ indicate less cross-modality stability for a cluster (i.e., more over-splitting).

##### Under-splitting score

If a cluster cannot be further split, its cells should be biologically equivalent to each other and differ only in terms of measurement noise. Otherwise, the cluster may be under-split. To quantify the equivalence of the cells within a cluster, we define the *self-radius* of a cell as the number of cells which appear equivalent to it in terms of consistent multimodal features. We first measured the distance,*d_ij_*, between the mC and RNA profiles of all cell pairs (*i,j*) after embedding in the common CCA space (see above). We reasoned that any cell pair whose distance is smaller than the distance between the mC and RNA profiles of cell *i* (i.e., *d*_*ij*_< *d_ii_*) can be considered equivalent; these cells are as similar to each other as they are to themselves. We thus define a cell’s self-radius, *r*_*i*_, as the number of equivalent cells; in terms of k-partners, this can be expressed as:

$$r_{i}=\arg\;\underset{k}{max}\left\{d_{ij}<d_{ii}\forall j\in P_{i}^{\left(k\right)}\right\}.$$

The distribution of the self-radii for cells in a cluster will inform us the extent to which the cluster under-split (Figure 2F). For example, if a cluster is not under-split at all, its cells’ self-radii should be uniformly distributed between zero and the cluster size. We verified this empirically with simulation: if we take a group of cells and randomly shuffle their gene-level profiles, we create a homogeneous cluster with no under-splitting. When we do this to all 17 major neuronal clusters, they all behave like ideal clusters without under-splitting (pink line in Figure 2F). Compared to the uniform distribution in the ideal case, an under-split cluster should have an overall much smaller self-radii, indicating it can be potentially further split into several sub-clusters (yellow line in Figure 2F). Therefore the slope of the cumulative distribution of self-radius informs us to what extent a cluster under-split. For an ideal cluster, its cumulative distribution of self-radii is a straight-line, therefore its slope is one. For an under-split cluster, the slope should be greater than one (Figure 2F). We, therefore, defined as the slope of the cumulative distribution of self-radius:

$$S_{under}\left(C_{i}\right)=\frac{Cumulative\;fraction\;of\;cells\;with\;r<=\left|C_{i}\right|/4}{\left|C_{i}\right|/4}$$

where the slope is evaluated at *r* = |*C_i_*|/4, as indicated in the above equation. For an ideal cluster, this score should be one; for an under-split cluster, it should be greater than one.

#### Computational data fusion with SingleCellFusion

Several computational methods have been proposed for integrating multiple single-cell sequencing datasets across batches, sequencing technologies, and modalities.^9,10,29,32,34^ Many of these methods share a basic strategy of identifying neighbor cells across datasets. However, existing methods have not been optimized to integrate single cells from multiple transcriptomic and epigenomic data modalities, with potentially large systematic differences in the features measured for each dataset. Here, we fused the transcriptomes and DNA methylomes of the snmCAT-Seq dataset, treating the two data modalities as if they were acquired by two independent single-modality experiments in different cells. We developed a new data fusion method, *SingleCellFusion,* for this task (available at: https://github.com/mukamel-lab/SingleCellFusion), which is based on finding k-partners, i.e., nearest neighbors across data modalities (see the previous section). Nearest neighbor based data integration has been successfully applied to combine multiple RNA-Seq datasets,^9,29^ while other approaches including canonical correlation analysis (CCA) and non-negative matrix factorization (NMF) have previously been used for the fusion of transcriptomic and epigenomic data.^10,32^ Single Cell Fusion is designed to robustly fuse DNA methylation, ATAC-Seq and/or RNA-Seq data. The procedure comprises 4 major steps: preprocessing: within-modality smoothing, cross-modality imputation, and clustering and visualization.

1. Preprocessing. We defined a gene-by-cell feature matrix for both transcriptomes and epigenomes. Transcriptomic features are log_10_(TPM+1) normalized. DNA methylation data is represented by the mean gene body mCH level, normalized by the global (genome-wide) mean mCH level for each cell. We selected genes with significantly correlated gene body mCH and RNA expression (FDR < 0.05) across neuronal cells as features (n = 5,107 genes).
2. Within-modality smoothing. To reduce the sparsity and noise of feature matrices, we share information among cells with similar profiles using data diffusion.^88^ First, we generate a kNN graph of cells based on Euclidean distances in PC space [ndim = 50, k = 30]. We next construct a sparse weighted adjacency matrix ***A***. We first apply a Gaussian kernel on the distance between cell i and cell j: $A_{ij}^{\left(1\right)}\;\propto \;exp\left(-d_{ij}^{2}/\sigma_{i}^{2}\right)$, where *σ_i_* is the distance to the ***k_a_***-th [***k_a_*** = 5] nearest neighbor of cell i. We set diagonal elements to zero, $A_{ii}^{\left(1\right)}=0$, and also set all elements to zero if they are not part of the kNN. We then symmetrize the matrix, ***A***^(2)^ = ***A***^(1)^ + ***A***^(1)^*τ*^^, and normalize each row: $A_{\mathrm{ij}}^{\left(3\right)}=A^{\left(2\right)}/a_{i}$, where $a_{i}=\underset{j}{\Sigma }A_{ij}^{\left(2\right)}$. Finally, we reweight the adjacency matrix with a parameter, ***p***, that explicitly controls the relative contribution of diagonal and non-diagonal elements: ***A*** = ***p I*** + (1 – *p*) ***A***^(3)^, where ***I*** is the identity matrix. We chose p = 0.9 for DNA methylation; p = 0.7 for RNA. Finally, we smooth the raw feature matrix by matrix multiplication with the adjacency matrix.
3. Cross-modality imputation by Restricted k-Partners (RKP). Each cell has a set of measured features in one data modality (RNA or mC), which we call the “source modality.” The goal of this step of the analysis is to impute the missing features from the other data type, called the “target modality.” For each cell in the source modality, we select a set of k-partners in the target modality and use the average of the k-partners’ features to estimate the missing modality for the original cell. However, care must be taken to avoid hub cells in the target modality which form k-partner relationships with a large fraction of all cells in the source modality. One way to avoid hub cells is by including only mutual nearest neighbors (MNN).^29^ We developed an alternative approach, restricted k-partners (RKP), that efficiently finds a set of k-partners for every source modality cell, while ensuring that every target-modality cell is connected with a roughly equal number of source modality cells.
  As above, we first reduce the dimensionality of both source and target data matrices by canonical correlation analysis, retaining the top 50 canonical components. We then iterate over all cells in the source modality (in random order) k times, connecting each with its most similar partner cell in the target modality. Whenever a target modality cell is partnered with more than ***k***′ source modality cells, we remove it from the pool of eligible target cells so that it will not be the partner of additional source cells. We set ***k***′ = [***z k N_source_/N_target_***]_+_, where and ***z*** ≥ 1 is a relaxation parameter that determines how much variability in the number of partners is allowed across target modality cells and []_+_ is the ceiling function. If ***z*** = 1 then every target cell will be connected to exactly ***k***′ or ***k***′ – 1 cells. We set ***z*** = 3, meaning that any individual target modality cell can have at most 3 times as many partners as the average. This algorithm is efficient and, in our analyses, provides robust k-partner graphs for cross-modality data imputation. Having determined each source cell’s restricted k-partners, we next impute the target features by averaging over the smoothed feature vectors of each cell’s k-partners.
4. Clustering and visualization. After imputation, we cluster and visualize cells from the 2 data modalities as if they are from the same dataset. We reduce dimensionality for all cells by performing PCA, keeping the top 50 PCs of the (measured and imputed) DNA methylation features. This cell-by-PCs matrix is further used for downstream embedding and clustering. Next, we perform UMAP embedding^29^ on the PC matrix [n_neighbors = 30, min_dist = 0.5]. Finally, we perform Leiden clustering (Traag^29^ on the kNN graph (symmetrized, unweighted) generated from the final PC matrix [Euclidean distance, k = 30, resolution = 0.3, 1, 2, 4].

#### Evaluation of Computational Data Fusion Methods

In Figure S3, we tested five data fusion tools: 1) Scanorama;^9^ 2) Harmony;^34^ 3) Seurat;^8^ 4) LIGER^10,89^ and 5) SingleCellFusion (the present study). For tools 1 to 3, we used the same set of highly variable genes (HVG, Top 2000 genes identified by Seurat FindVariableGenes function) identified from the transcriptome matrix as starting features; for algorithms 4 and 5, we used top 5000 genes having the highest correlation between their RNA and mCH level. These genes were chosen based on their overall accuracy (see below). We reversed the methylation values (i.e., max(X) - X, where X denotes the cell-by-gene mCH fraction matrix) before data fusion to account for the negative correlation of mCH fraction and RNA expression.

Below we describe the data fusion process of each tool, starting from the per cell normalized RNA-HVG matrix and reversed mCH-HVG matrix. After obtaining the decomposed matrix (PCs from 1,2,3,5 or H matrix from 4), we then evaluate the data fusion performance using metrics described below. For reproducibility, we uploaded all the steps and input files here: https://github.com/lhqing/snmCAT-seq_integration

1. For Scanorama, we used these parameters (sigma = 100, alpha = 0.1, knn = 30) to perform the data fusion and dimension reduction using Scanorama V1.7 on the scaled (via scanpy.pp.scale) mC and RNA matrix. We used the top 20 fused PCs (n_components = 20) for data fusion evaluation.
2. Unlike Scanorama, Harmony directly takes dimension reduction matrices as input. Therefore, we first run PCA separately (n_components = 20) on the scaled mCH and RNA matrix first, and run Harmony (pyharmony from https://github.com/jterrace/pyharmony) with default parameters on the concatenated PCs. Fused PCs generated by Harmony were then used for evaluation.
3. For Seurat, we followed the Seurat (v4.0.0) vignette steps to perform data fusion (https://satijalab.org/seurat/articles/integration_introduction.html). When calculating anchors for data fusion (FindTransferAnchors), we use the RNA matrix as the reference matrix and mCH matrix as the query matrix and using CCA as the dimension reduction method. We then transfer the mCH matrix to the RNA space using the anchors and run PCA (n_components = 20) on the concatenated (mCH and RNA) matrix after the transfer.
4. For LIGER, we followed the tutorial from developers (http://htmlpreview.github.io/?https://github.com/welch-lab/liger/blob/master/vignettes/online_iNMF_tutorial.html) and used the online_iNMF algorithm (Gao et al., 2020) with default parameters to perform data fusion and used the normalized matrix H (the cells’ decomposed matrix from the online iNMF algorithm) for fusion evaluation.

Finally, the SingleCellFusion analysis was described in the manuscript, we used the fused PCs for evaluation.

##### Metrics for evaluation of data fusion

We used three different approaches to evaluate the single-cell data fusion results. First, We ran UMAP on the decomposed matrix from each tool to provide an overview of the fused dataset.

Second, We utilize the ground-truth information from the snmCAT-seq to calculate a self-radius at the single-cell level. Specifically, we first construct a nearest-neighbor index using Annoy (v1.17.0) on the decomposed matrix (euclidean distance). For the same cell, if its RNA vector is the mCH vector’s Kth neighbor, we then use d = K as the self-radius. The quality of the data fusion can be normalized by d/2N, where N is the total number of snmCAT-seq cells involved in the analysis. The value of d/2N ranges from 0 to 1, with smaller values indicating good fusion and larger values indicating inadequate fusion of mC and RNA profiles of the same cell.

Finally, we performed Leiden co-clustering on the decomposed matrix (with different resolution parameters to obtain 17 co-clusters in all tools, which is the number of major neuronal cell types) and calculated the co-cluster accuracy as the fraction of cells whose RNA and mC profiles were assigned to the same cluster. This accuracy can be calculated for each co-cluster or the whole dataset. Higher accuracy means good fusion, and a low accuracy indicates inadequate fusion.

#### Correlation analysis of RNA expression and gene body DNA methylation

For each gene, we compute the Spearman correlation coefficient between RNA expression (log_10_(TPM+1)) and gene body mCH (normalized to global mCH of each cell). To determine if a correlation is statistically significant, we randomly shuffled cell labels to generate an empirical null distribution. Significantly correlated genes are defined with empirical FDR < 0.05. Applying this method to 3,898 neurons in the snmCAT-seq dataset, we get 5,145 genes with a significant negative correlation between RNA and mCH (RNA-mCH coupled).

#### Eta Squared of Genes Across Clusters

For each gene used for correlation analysis (Figure 3) we compute the *η*^2^ across neuronal sub clusters (n = 52) generated from ensemble methylomes (Figure 4) for both RNA (log_10_(TPM+1)) and gene body mCH (normalized by global mCH of each cell) signals. We also compute *η*^2^ across 10X RNA-seq clusters for the same genes.

#### H3K27me3 ChIP-Seq data processing

We downloaded published H3K27me3 ChIP-Seq data of purified excitatory and inhibitory neurons from the human prefrontal cortex.^36^ We calculated the average ChIP-Seq signal intensity (RPKM) across the gene body for excitatory and inhibitory neurons.

#### Fusion of DNA methylome and snATAC-Seq data

Ensemble methylomes and snATAC-seq data from neurons and glia were fused separately using our recently developed Single Cell Fusion method (see section Computational data fusion with Single CellFusion). The top 4000 variable genes across clusters in the snmCAT-seq and snATAC-seq data were identified using a Kruskal-Wallis test; 1,652 genes were identified as being variable in both datasets and were used for the subsequent data fusion. For snATAC-seq the gene body was extended to include the promoter region (2kb upstream TSS). Prior to the fusion of mCH and open chromatin levels at gene bodies were smoothed to reduce sparseness (k = 20, ka = 4, epsilon = 1, p = 0.9; see section Within-modality smoothing) using a diffusion-based smoothing method adapted from MAGIC.^88^ A constrained k-nearest neighbors graph was generated among cells across 2 datasets (k = 20, z = 10; see section Cross-modality imputation by Restricted k-Partners). Instead of calculating Euclidean distance in reduced dimensions, here we simply used Spearman correlation across 1,652 genes as the distance measure between cells. We used the kNN graph to impute the gene body mCH profile for each ATAC-Seq nucleus. The observed (ensemble methylomes) and imputed (snATAC-Seq nuclei) gene body mCH levels were then jointly used for Leiden clustering and UMAP embedding. Each snATAC-seq nucleus was assigned to a major cell type if at least half of its restricted k-Partners belonged to that cell type, the remaining cells were removed from subsequent analysis (n = 499, 3.98%).

#### snmCAT-seq - snRNA-seq integration

To perform the integration analysis of snmCAT-seq transcriptome and snRNA-seq, we separate the cells into three broad classes: excitatory neurons, inhibitory neurons, and non-neuronal cells. The RNA features used for the integration by Scanorama come from two sources for each cell class: 1) highly variable genes across individual cells; 2) cluster level RNA marker genes. To validate that the cluster level RNA marker genes are relevant for neuronal processes, we performed a synapse-specific GO enrichment test using the SynGO terms and all brain expressed genes as background.^40^ The -log(adjusted P value) of SynGO biological process enrichment in each selected gene set is color-coded on the sunburst chart of the hierarchical SynGO terms (Figures 5E and 5F).

We then used the union of RNA features found in snmCAT-seq transcriptome and snRNA-seq for Scanorama integration and PCA calculation. The dominant PCs were then used to perform a co-clustering analysis on the cells profiled by snmCAT-seq cell or snRNA-seq. Instead of directly using the co-clustering results, we used this intermediate clustering assignment to calculate the overlap score between the original methylome ensemble clusters and the snRNA-seq clusters. The overlap score range from 0 to 1 is defined as the sum of the minimum proportion of samples in each cluster that overlapped within each co-cluster,^38^ a higher score between one methylome cluster and one snRNA-seq cluster indicate they consistently co-clustered.

#### Cell type dendrogram and sub-cluster merge along the lineage

The cell-type hierarchy of inhibitory and excitatory cells was calculated separately using the concatenated PCs from mCG and mCH as the features used for computing cluster centroids. We used *scipy.cluster.hierarchy.linkage* function to calculate the ward linkage. Based on the linkage results, we merged the CpG sites from single-cell ALLC files in 2 steps: 1) we merged the single-cell ALLC files into each of the sub-clusters, 2) we then merge the sub-clusters into all nodes that appeared in the dendrograms. The merged CpG ALLC files are then used in the lineage-DMR analysis.

#### Neural lineage-specific DMR calling and motif enrichment analysis

We used the *methylpy findDMR* function^52^ to identify mCG lineage-DMRs for each pair of lineages using merged ALLC files. The DMRs identified by methylpy in each branch comparison are further filtered by mCG rate difference > 0.3 and the number of differentially methylated sites (DMS) > = 2. Lineage pairs with > 10^4^ DMRs identified were used for motif enrichment analysis and TF marker identification. For each of these DMR sets, we use AME^90^ to perform motif enrichment (fisher’s exact test) analysis with the motifs’ Position Weight Matrix (PWM) from the JASPAR database (JASPAR2018 CORE Vertebrates).^91^ The DMRs are length standardized into ± 250bp of region center before motif scanning. Tissue-specific DMRs (without brain tissue, and standardized in the same way) from the Roadmap Epigenomics project^52,55^ were used as the background.

#### TF binding preference to methylated motifs

To further investigate the methylation level impact on the potential TF binding sites, we selected all the mCG DMSs ± 25bp regions from the branch-DMRs and ran motif enrichment using motifs identified from the methyl-SELEX experiment.^45^ In each branch pair, we used the left-DMSs as the background of right-DMS to find the right-branch-specific motif and vice versa. The significantly enriched “TF motif - branch” combinations were then intersected with the corresponding branch pair’s DEG and DMG list to infer their gene mCH or RNA specificity.

#### Chromatin accessibility analysis of TF binding motifs

Genome-wide sites matching TF binding motifs (motif matches) identified by methyl-SELEX^45^ were identified using FIMO 4.11.4^92^ with the following parameters–max-stored-scores 500000–max-strand–thresh 1e-5. Methyl-SELEX only quantified the effect of CpG methylation on TF binding. Therefore only genomics sites containing CG dinucleotides were selected for further analyses. For each major cell type, the density of methylated GCY sites or ATAC-seq reads was quantified for motif matches that overlap with hypomethylated or hypermethylated DMRs. Figures 6F and S6I show the average chromatin accessibility at motif matches across major cell types. TF binding motifs were ranked by the difference of chromatin accessibility between motif matches located in hypomethylated and hypermethylated DMRs. To test the enrichment of MethylPlus and MethylMinusTFs, the ranked motif list was divided into 5 bins and the enrichment or depletion in each bin was tested using MATLAB *hygecdf* function.

#### Partitioned heritability analysis

Bulk human fetal frontal cortex methylomes from a PCW 20 donor^22^ and a PCW 19 donor^37^ were previously published. Fetal frontal cortex DMRs were identified using *methylpy findDMR* function^52^ by comparing to adult bulk neuronal (NeuN+) and non-neuronal (NeuN−) methylomes.^22^ Fetal brain Dnase-seq samples included fetal day 85d (GSM595922, GSM595923), 96d (GSM595926, GSM595928), 101d (GSM878650), 104d (GSM878651), 105d (GSM1027328), 109d (GSM878652), 112d (GSM665804), 117d (GSM595920) and 142d (GSM665819).^55^ Mapped reads files (BED format) were downloaded followed by DNase-seq peak calling using MACS2 2.0.10 with q-value < 0.01. Fetal brain DNase-seq peaks were defined as the union DNase-seq peaks of fetal brain DNase-seq datasets and were supported by at least two samples.

Summary statistics were downloaded from the Psychiatric Genomics Consortium portal (https://www.med.unc.edu/pgc/) for neuropsychiatric trait GWAS - ADHD,^93^ Aggression,^94^ Anorexia nervosa,^95^ Anxiety,^96^ ASD,^97^ Bipolar,^98^ Cognitive Performance,^99^ Educational Attainment,^99^ Alzheimer’s,^100^ Internalizing, Loneliness,^101^ Major Depression,^102^ Neuroticism,^103^ OCD,^104^ Schizophrenia (PGC2)^105^ and Schizophrenia (PGC1).^106^

The partitioned heritability analysis was performed using LD Score Regression (LDSC) Partitioned Heritability.^54^ The partitioned heritability analysis was performed by constructing joint linear models by providing multiple regulatory element annotations in addition to the “baseline” annotation. Alternatively, we performed analyses by constructing individual models by comparing each annotation of regulatory elements individually against the “baseline.” We built a “baseline” annotation using tissue-specific DMRs from non-brain human tissues^52^ to control for generic gene regulation characteristics. Partitioned heritability analyses using cell-type specifically expressed genes were performed as described in.^56^ The reported q-values were derived from the *“Coefficient_z.score”* values reported by LDSC Partitioned Heritability.

#### Prioritization of trait-associated cell types using RolyPoly

Although RolyPoly was originally developed to associate GWAS summary statistics with transcriptome data, we adapted the method to analyze epigenomic features such as DMRs and ATAC-seq peaks. RolyPoly analysis was performed using summary statistics for schizophrenia,^105^ bipolar disorder,^98^ ASD^97^ and educational attainment.^99^ DMRs or ATAC-seq peaks identified for each cell type that overlapped with the top 10,000 variants with the smallest p value were provided as the feature list. As recommended by the RolyPoly tutorial (https://github.com/dcalderon/rolypoly), the absolute value of Z-scores computed for CG methylation level or ATAC-seq signal across samples were provided as in place of expression data. The analysis was performed with 100 times bootstrapping.

## Supplementary Material

1

Table S9

Table S10

Table S1

Table S2

Table S3

Table S4

Table S5

Table S6

Table S7

Table S8

## Figures and Tables

**Table 1.** Genomic profiling methods discussed in this study

Method	Description	Reference
snmC-seq	Multiplexed single-nucleus DNA methylome profiling	Luo et al.^4^
snmC-seq2	Improved single-nucleus DNA methylome profiling methods with increased read mapping and enhanced throughput	Luo et al.^16^
snmCAT-seq	Single-nucleus joint profiling of DNA methylome, chromatin accessibility (based on NOMe-seq), and transcriptome	This study
sn-m3C-seq	Single-nucleus joint profiling of chromatin conformation and DNA methylome	Lee et al.^15^
NOMe-seq	Profiling of nucleosome footprint and chromatin accessibility using *in vitro* GpC methyltransferase labeling	Kelly et al.^17^
Smart-seq2	The generation and amplification of full-length cDNA and sequencing libraries	Picelli et al.^18^
snRNA-seq	Single-nucleus RNA-seq. The human brain snRNA-seq in this study was generated using the 10x Genomics Chromium platform.	This study
ATAC-seq	Assay for chromatin accessibility using Tn5 transposon	Buenrostro et al.^19^
snATAC-seq	Combinatorial indexing-assisted single-cell assay for transposase-accessible chromatin	Preissl et al.^5^

**Table:** KEY RESOURCES TABLE

REAGENT or RESOURCE	SOURCE	IDENTIFIER
Experimental models: Cell lines		
HEK293T cells	Salk Institute Stem Cell Core	N/A
H1 hESC cells	WiCell	WA01
Experimental models: Organisms/strains		
Brodmann area 10 (M_21yr)	NIH NeuroBioBank at University of Maryland Brain and Tissue Bank	UMB5577
Brodmann area 10 (M_29yr)	NIH NeuroBioBank at University of Maryland Brain and Tissue Bank	UMB5580
Medial Frontal Gyrus (M_25yr_1)	NIH NeuroBioBank at University of Maryland Brain and Tissue Bank	UMB4540
Brodmann area 10 (M_58yr)	NIH NeuroBioBank at University of Miami Brain Endowment Bank	NDARKD326LNK
Brodmann area 10 (M_25yr_2)	NIH NeuroBioBank at University of Miami Brain Endowment Bank	NDARKJ183CYT
Brodmann area 44-45, Brodmann area 46	Allen Institute for Brain Science	H18.30.002
Chemicals, peptides, and recombinant proteins		
Custom Tn5 Transposase	MacroLab, University of California Berkeley	Custom Protein Purification
Deposited data		
snmCAT-seq data generated from HEK293T and H1 hESC cells	This Study	GEO: GSE140493
snmCAT-seq data generated from UMB5577 and UMB5580	This Study	GEO: GSE140493
snmC-seq and snmC-seq2 data generated from NDARKD326LNK and NDARKJ183CYT	This Study	GEO: GSE140493
snATAC-seq data generated from UMB4540	This Study	GEO: GSE140493
scRNA-seq data generated from H18.30.002	This Study	NeMO: dat-s3creyz
sn-m3C-seq data generated from UMB5577 and UMB5580	Lee et al.^15^	GEO: GSE130711
snmC-seq data generated from UMB4540	Luo et al.^4^	GEO: GSE97179
H3K27me3 ChIP-seq	Kozlenkov et al.^36^	Synapse (syn12034263)
Oligonucleotides		
dT30VN_4	Integrated DNA Technologies	5′-/5SpC3/AAGCAGUGGUAUCAACGCAGAGUACUTTTTTUTTTTTUTTTTTUTTTTTUTTTTTVN-3′ (HPLC purified)
N6_2	Integrated DNA Technologies	5′-/5SpC3/AAGCAGUGGUAUCAACGCAGAGUACNNNNNN-3′ (HPLC purified)
TSO_3	Exiqon (now QIAGEN)	5′-/5SpC3/AAGCAGUGGUAUCAACGCAGAGUGAAUrGrG+G-3′ (HPLC purified)
ISPCR23_2	Integrated DNA Technologies	5′-/5SpC3/AAGCAGUGGUAUCAACGCAGAGU-3′ (HPLC purified)
Software and algorithms		
SingleCellFusion	This Study	https://github.com/mukamel-lab/SingleCellFusion
LIGER	Welch et al.^10^	https://github.com/welch-lab/liger
Bismark v0.14.4	Krueger and Andrews^70^	https://www.bioinformatics.babraham.ac.uk/projects/bismark/; RRID: SCR_005604
STAR 2.5.2b	Dobin et al.^71^	https://github.com/alexdobin/STAR; RRID: SCR_015899
YAP	Liu et al.^23^	https://hq-1.gitbook.io/mc/
ALLCools	Liu et al.^23^	https://github.com/lhqing/ALLCools
methylpy	Schultz et al.^52^	https://github.com/yupenghe/methylpy
Seurat v4.0.0	Stuart et al.^8^	https://satijalab.org/seurat/; RRID: SCR_016341
Scanorama v1.7	Hie et al.^9^	https://github.com/brianhie/scanorama
Harmony (pyharmony)	Korsunsky et al.^34^	https://github.com/iandday/pyharmony

## Highlights

- Joint single-nucleus multi-omic profiling in human tissues
- Assessment of computational data integration methods using multi-modal measurements
- Diverse relationships between DNA methylation and gene expression in individual cells
- Reconstruction of regulatory hierarchy for 63 human cortical cell populations
