# Supplementary material for: Single nucleus multi-omics identifies human cortical cell regulatory genome diversity
Source: Cell Genom. 2022 Mar 9;2(3):100107. doi: 10.1016/j.xgen.2022.100107 (PMC9004682; doi:10.1016/j.xgen.2022.100107)
Supplement: Document S1. Figures S1–S7 [file mmc1.pdf]

Cell Genomics, Volume *2*

Supplemental information

Single nucleus multi-omics identifies human cortical cell regulatory genome diversity

Chongyuan Luo, Hanqing Liu, Fangming Xie, Ethan J. Armand, Kimberly Siletti, Trygve E. Bakken, Rongxin Fang, Wayne I. Doyle, Tim Stuart, Rebecca D. Hodge, Lijuan Hu, Bang-An Wang, Zhuzhu Zhang, Sebastian Preissl, Dong-Sung Lee, Jingtian Zhou, Sheng-Yong Niu, Rosa Castanon, Anna Bartlett, Angeline Rivkin, Xinxin Wang, Jacinta Lucero, Joseph R. Nery, David A. Davis, Deborah C. Mash, Rahul Satija, Jesse R. Dixon, Sten Linnarsson, Ed Lein, M. Margarita Behrens, Bing Ren, Eran A. Mukamel, and Joseph R. Ecker

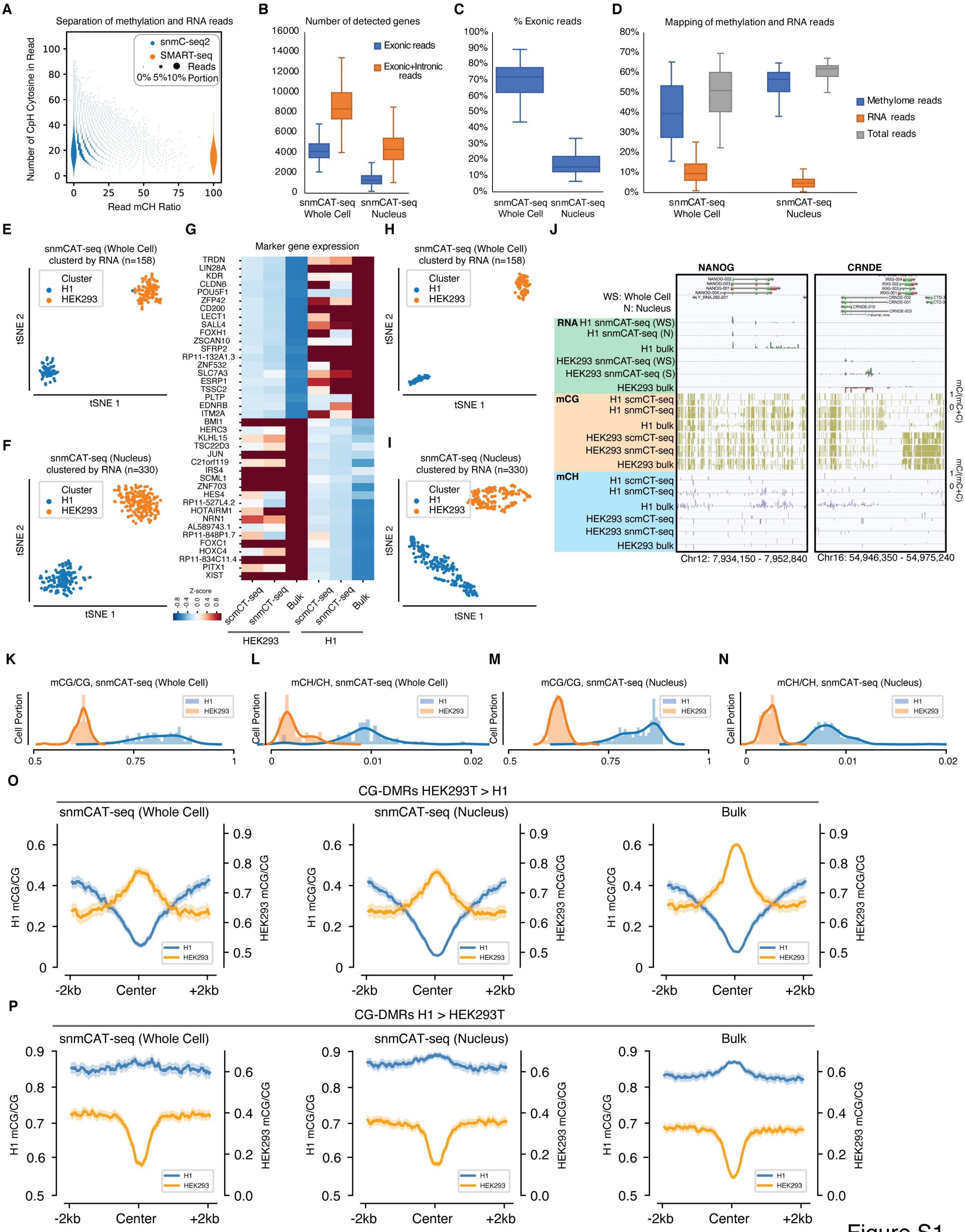
A
Separation of methylation and RNA reads
Number of CpH Cytosine in Read
Read mCH Ratio
snmC-seq2
SMART-seq
Reads
0% 5%10% Portion
B
Number of detected genes
Exonic reads
Exonic+Intronic reads
snmCAT-seq Whole Cell
snmCAT-seq Nucleus
C
% Exonic reads
snmCAT-seq Whole Cell
snmCAT-seq Nucleus
D
Mapping of methylation and RNA reads
Methylome reads
RNA reads
Total reads
snmCAT-seq Whole Cell
snmCAT-seq Nucleus
E
snmCAT-seq (Whole Cell) clustered by RNA (n=158)
Cluster
H1
HEK293
tSNE 1
tSNE 2
F
snmCAT-seq (Nucleus) clustered by RNA (n=330)
Cluster
H1
HEK293
tSNE 1
tSNE 2
G
Marker gene expression
TRDN
LIN28A
KDR
CLDN6
POU5F1
ZFP42
CD200
LECT1
SALL4
FOXH1
ZSCAN10
SFRP2
RP11-132A1.3
ZNF532
SLC7A3
ESRP1
TSSC2
PLTP
EDNRB
ITM2A
BMI1
HERC3
KLHL15
TSC22D3
JUN
C21orf119
IRS4
SCML1
ZNF703
HES4
RP11-527L4.2
HOTAIRM1
NRN1
AL589743.1
RP11-848P1.7
FOXC1
HOXC4
RP11-834C11.4
PITX1
XIST
Z-score
scmCT-seq
snmCT-seq
Bulk
HEK293
H1
H
snmCAT-seq (Whole Cell) clustered by RNA (n=158)
Cluster
H1
HEK293
tSNE 1
tSNE 2
I
snmCAT-seq (Nucleus) clustered by RNA (n=330)
Cluster
H1
HEK293
tSNE 1
tSNE 2
J
NANOG
CRNDE
WS: Whole Cell
N: Nucleus
RNA H1 snmCAT-seq (WS)
H1 snmCAT-seq (N)
H1 bulk
HEK293 snmCAT-seq (WS)
HEK293 snmCAT-seq (S)
HEK293 bulk
mCG H1 scmCT-seq
H1 snmCT-seq
H1 bulk
HEK293 scmCT-seq
HEK293 snmCT-seq
HEK293 bulk
mCH H1 scmCT-seq
H1 snmCT-seq
H1 bulk
HEK293 scmCT-seq
HEK293 snmCT-seq
HEK293 bulk
mC/(mC+C)
Chr12: 7,934,150 - 7,952,840
Chr16: 54,946,350 - 54,975,240
K
mCG/CG, snmCAT-seq (Whole Cell)
Cell Portion
H1
HEK293
L
mCH/CH, snmCAT-seq (Whole Cell)
Cell Portion
H1
HEK293
M
mCG/CG, snmCAT-seq (Nucleus)
Cell Portion
H1
HEK293
N
mCH/CH, snmCAT-seq (Nucleus)
Cell Portion
H1
HEK293
O
CG-DMRs HEK293T > H1
snmCAT-seq (Whole Cell)
snmCAT-seq (Nucleus)
Bulk
H1 mCG/CG
HEK293 mCG/CG
H1
HEK293
-2kb
Center
+2kb
P
CG-DMRs H1 > HEK293T
snmCAT-seq (Whole Cell)
snmCAT-seq (Nucleus)
Bulk
H1 mCG/CG
HEK293 mCG/CG
H1
HEK293
-2kb
Center
+2kb

Figure S1

Figure. S1 snmCAT-seq captures transcriptome and DNA methylation signatures of H1 & HEK293 cells, related to Figure 1. (A) The specificity for classifying methylation (snmC-seq2) and transcriptome (snRNA-seq) reads plotted as a function of the number of CpH cytosine in the reads. (B-D) The number of detected genes (B), percentage of mapped reads that are located in exons (C), and mapping rates of methylation and RNA reads (D) for snmCAT-seq (Whole Cell) and snmCAT-seq (Nucleus). (E-F) Separation of H1 and HEK293T cells by tSNE using transcriptome reads extracted from snmCAT-seq (Whole Cell) (E) or snmCAT-seq (Nucleus) (F) datasets. (G) snmCAT-seq (Whole Cell) and snmCAT-seq (Nucleus) detect genes specifically expressed in H1 or HEK293T cells. (H-I) Separation of H1 and HEK293T cells by tSNE using DNA methylation information extracted from snmCAT-seq (Whole Cell) (H) or snmCAT-seq (Nucleus) (I) datasets. (J) Browser view of NANOG and CRNDE loci. (K-N) Distribution of mCG and mCH levels for single H1 and HEK293 cells/nuclei as determined by snmCAT-seq (Whole Cell) and snmCAT-seq (Nucleus). (O-P) snmCAT-seq (Whole Cell) and snmCAT-seq (Nucleus) recapitulate bulk mCG patterns at CG-DMRs showing greater mCG levels in HEK293T (O) or H1 (P) cells.

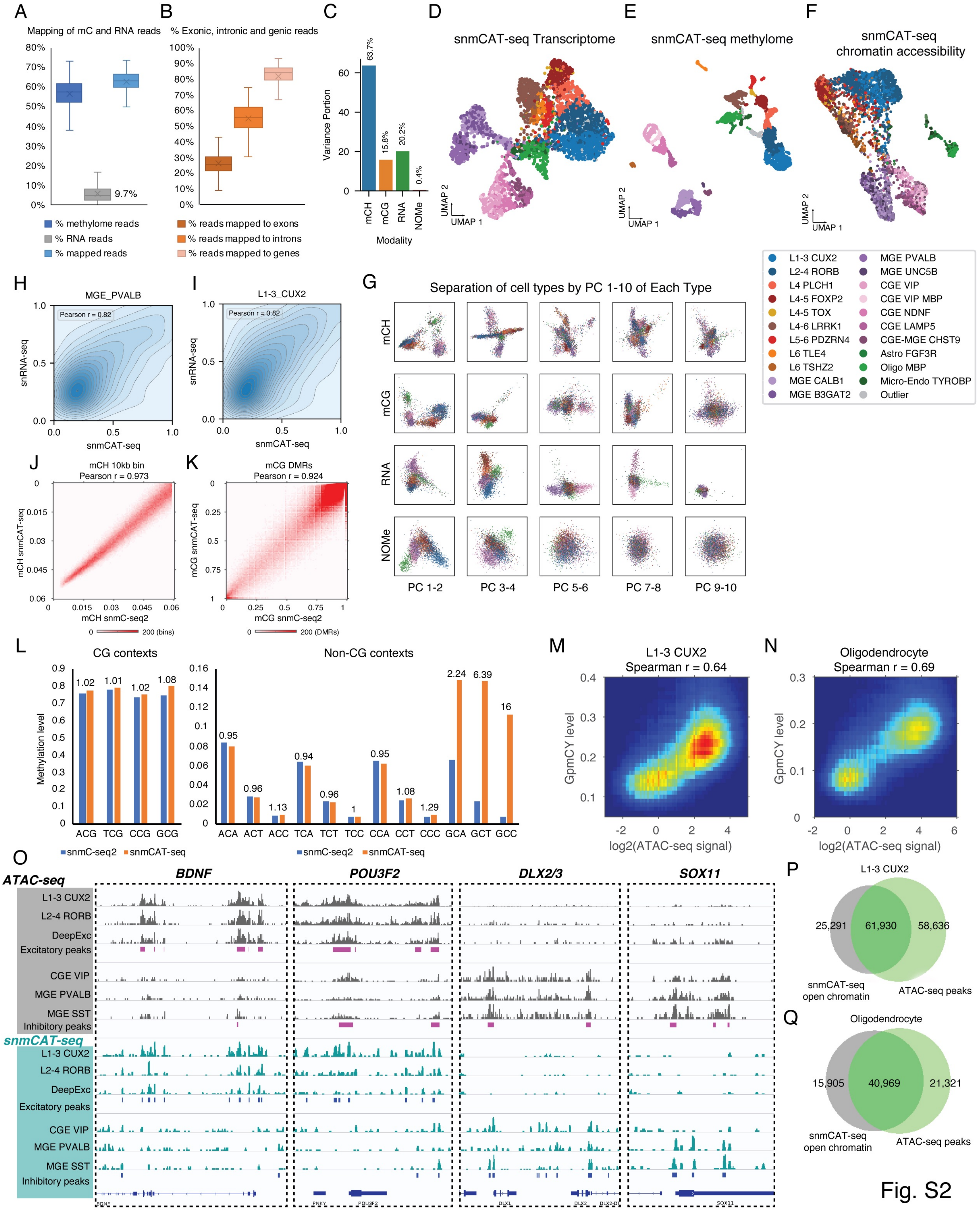

A
Mapping of mC and RNA reads
% methylome reads
% RNA reads
% mapped reads
9.7%
B
% Exonic, intronic and genic reads
% reads mapped to exons
% reads mapped to introns
% reads mapped to genes
C
Variance Portion
Modality
63.7%
15.8%
20.2%
0.4%
mCH
mCG
RNA
NOMe
D
snmCAT-seq Transcriptome
E
snmCAT-seq methylome
F
snmCAT-seq chromatin accessibility
UMAP 1
UMAP 2
L1-3 CUX2
L2-4 RORB
L4 PLCH1
L4-5 FOXP2
L4-5 TOX
L4-6 LRRK1
L5-6 PDZRN4
L6 TLE4
L6 TSHZ2
MGE CALB1
MGE B3GAT2
MGE PVALB
MGE UNC5B
CGE VIP
CGE VIP MBP
CGE NDNF
CGE LAMP5
CGE-MGE CHST9
Astro FGF3R
Oligo MBP
Micro-Endo TYROBP
Outlier
H
MGE_PVALB
Pearson r = 0.82
snRNA-seq
snmCAT-seq
I
L1-3_CUX2
Pearson r = 0.82
G
Separation of cell types by PC 1-10 of Each Type
mCH
mCG
RNA
NOMe
PC 1-2
PC 3-4
PC 5-6
PC 7-8
PC 9-10
J
mCH 10kb bin
Pearson r = 0.973
mCH snmCAT-seq
mCH snmC-seq2
200 (bins)
K
mCG DMRs
Pearson r = 0.924
mCG snmCAT-seq
mCG snmC-seq2
200 (DMRs)
L
CG contexts
Non-CG contexts
Methylation level
1.02
1.01
1.02
1.08
ACG
TCG
CCG
GCG
0.95
0.96
1.13
0.94
0.96
1
0.95
1.08
1.29
2.24
6.39
16
ACA
ACT
ACC
TCA
TCT
TCC
CCA
CCT
CCC
GCA
GCT
GCC
snmC-seq2
snmCAT-seq
M
L1-3 CUX2
Spearman r = 0.64
GpmCY level
log2(ATAC-seq signal)
N
Oligodendrocyte
Spearman r = 0.69
O
ATAC-seq
BDNF
POU3F2
DLX2/3
SOX11
L1-3 CUX2
L2-4 RORB
DeepExc
Excitatory peaks
CGE VIP
MGE PVALB
MGE SST
Inhibitory peaks
snmCAT-seq
P
L1-3 CUX2
25,291
61,930
58,636
snmCAT-seq open chromatin
ATAC-seq peaks
Q
Oligodendrocyte
15,905
40,969
21,321
snmCAT-seq open chromatin
ATAC-seq peaks

Fig. S2

Figure S2. snmCAT-seq generates single-nucleus multi-omic profiles from human brain tissues, related to Figure 1. (A) The fraction of total snmCAT-seq reads derived from methylome and transcriptome. (B) The fraction of snmCAT-seq transcriptome reads mapped to exons, introns or gene bodies. (C) The portion of variance explained by each data modality. (D-F) UMAP embedding of 4253 snmCAT-seq cells using single modality information: transcriptome (D), methylome (mCH and mCG, E) and chromatin accessibility (F). (G) The separation of cell types by the top 10 principal components (PCs) of each data type. (H-I) Pearson correlation of gene expression quantified by snmCAT-seq transcriptome and snRNA-seq in MGE PVALB (H) and L1-3 CUX2 (I) cells. (J) Pearson correlation of gene body non-CG methylation quantified with snmCAT-seq methylome and snmC-seq for MGE PVALB cells. (K) Pearson correlation of CG methylation at DMRs quantified with snmCAT-seq methylome and snmC-seq for MGE PVALB cells. (L) Genome-wide methylation level for all tri-nucleotide context (-1 to +2 position) surrounding cytosines shows the sequence specificity of GpC methyltransferase M.CviPI. (M-N) Spearman correlation between GCY methylation level and ATAC-seq signal at open chromatin sites in L1-3 CUX2 (M) and Oligodendrocyte (N) cells. (Q) Browser views of chromatin accessibility profiles generated by snmCAT-seq and snATAC-seq at cell-type-specific genes. (P-Q) Overlap of open chromatin peaks identified by snmCAT-seq and snATAC-seq in L1-3 CUX2 (P) and oligodendrocyte (Q) cells.

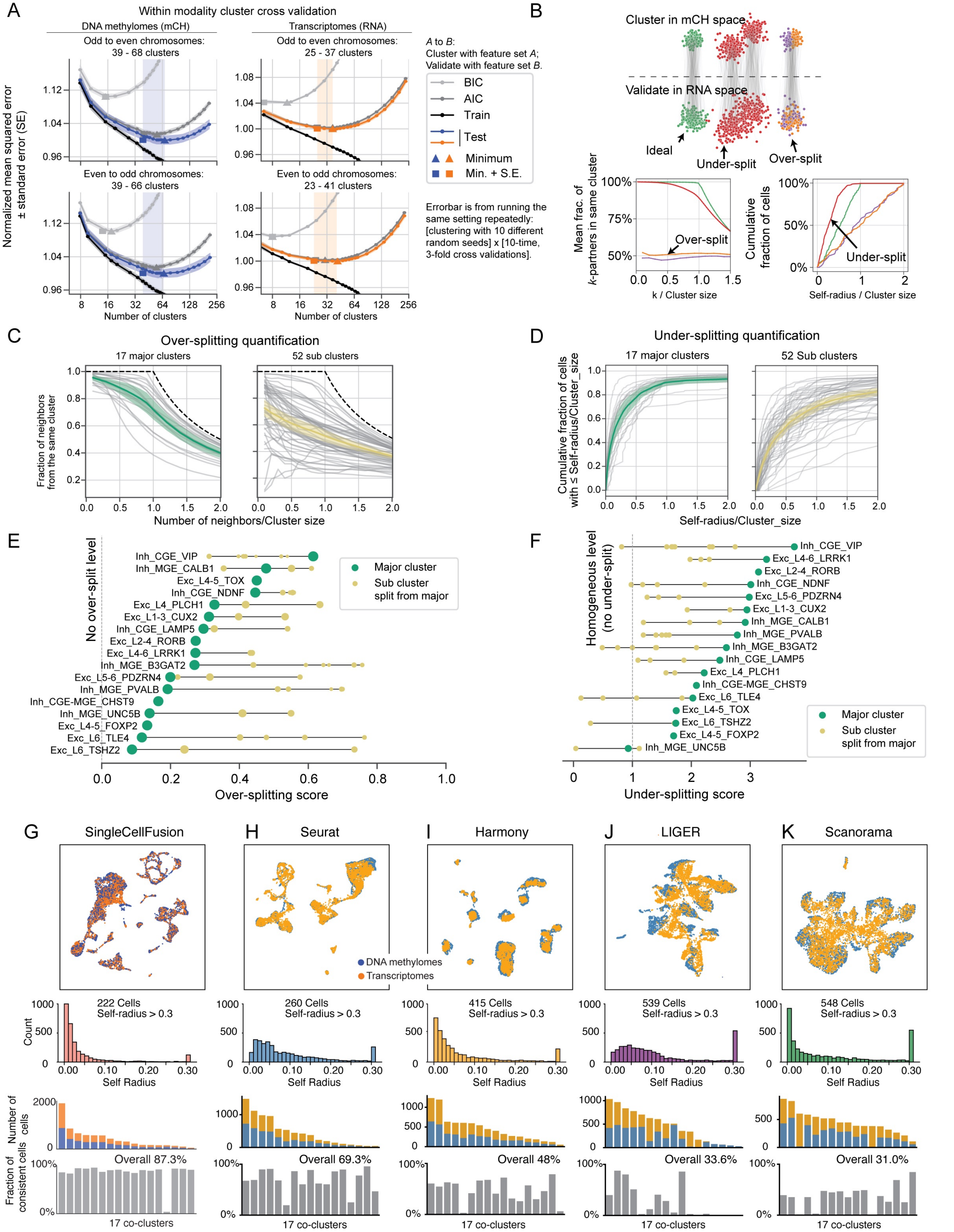
A
Within modality cluster cross validation
DNA methylomes (mCH)
Transcriptomes (RNA)
Odd to even chromosomes: 39 - 68 clusters
Odd to even chromosomes: 25 - 37 clusters
Even to odd chromosomes: 39 - 66 clusters
Even to odd chromosomes: 23 - 41 clusters
Normalized mean squared error ± standard error (SE)
Number of clusters
A to B: Cluster with feature set A; Validate with feature set B.
BIC
AIC
Train
Test
Minimum
Min. + S.E.
Errorbar is from running the same setting repeatedly: [clustering with 10 different random seeds] x [10-time, 3-fold cross validations].
B
Cluster in mCH space
Validate in RNA space
Ideal
Under-split
Over-split
Mean frac. of k-partners in same cluster
k / Cluster size
Cumulative fraction of cells
Self-radius / Cluster size
C
Over-splitting quantification
17 major clusters
52 sub clusters
Fraction of neighbors from the same cluster
Number of neighbors/Cluster size
D
Under-splitting quantification
17 major clusters
52 Sub clusters
Cumulative fraction of cells with ≤ Self-radius/Cluster_size
Self-radius/Cluster_size
E
No over-split level
Major cluster
Sub cluster split from major
Inh_CGE_VIP
Inh_MGE_CALB1
Exc_L4-5_TOX
Inh_CGE_NDNF
Exc_L4_PLCH1
Exc_L1-3_CUX2
Inh_CGE_LAMP5
Exc_L2-4_RORB
Exc_L4-6_LRRK1
Inh_MGE_B3GAT2
Exc_L5-6_PDZRN4
Inh_MGE_PVALB
Inh_CGE-MGE_CHST9
Inh_MGE_UNC5B
Exc_L4-5_FOXP2
Exc_L6_TLE4
Exc_L6_TSHZ2
Over-splitting score
F
Homogeneous level (no under-split)
Inh_CGE_VIP
Exc_L4-6_LRRK1
Exc_L2-4_RORB
Inh_CGE_NDNF
Exc_L5-6_PDZRN4
Exc_L1-3_CUX2
Inh_MGE_CALB1
Inh_MGE_PVALB
Inh_MGE_B3GAT2
Inh_CGE_LAMP5
Exc_L4_PLCH1
Inh_CGE-MGE_CHST9
Exc_L6_TLE4
Exc_L4-5_TOX
Exc_L6_TSHZ2
Exc_L4-5_FOXP2
Inh_MGE_UNC5B
Major cluster
Sub cluster split from major
Under-splitting score
G SingleCellFusion
H Seurat
I Harmony
J LIGER
K Scanorama
DNA methylomes
Transcriptomes
222 Cells Self-radius > 0.3
260 Cells Self-radius > 0.3
415 Cells Self-radius > 0.3
539 Cells Self-radius > 0.3
548 Cells Self-radius > 0.3
Count
Self Radius
Number of cells
Overall 87.3%
Overall 69.3%
Overall 48%
Overall 33.6%
Overall 31.0%
Fraction of consistent cells
17 co-clusters

Figure S3

Figure S3. Evaluation of cluster quality with paired transcriptome and methylome profiles, related to Figure 2. (A) Intra-modality cross-validation of mCH- or RNA-defined clusters. Line plots show mean squared error between the single-cell profiles and cluster centroid, Akaike Information Criterion (AIC) and the Bayesian Information Criterion (BIC) as a function of the number of clusters. The shaded region in each sub-plot highlights the range between the minimum and the minimum + standard error for the curve of test-set error. For the analysis using snmCAT-seq mC information (left panels), gene body mCH profiles of odd (even) chromosomes were used for clustering whereas even (odd) chromosomes were used for testing. A similar analysis was performed using snmCAT-seq transcriptome information (right panels). (B) Schematic diagram of the over- and under-splitting analysis using matched single-cell methylome and transcriptome profiles, complementing Figure 2D. (C) The over-splitting quantification of mC-defined major clusters (n=17) and subclusters (n=52) was quantified by the fraction of cross-modal neighbors found in the same cluster defined by RNA. (D) The under-splitting of clusters was quantified as the cumulative distribution function of normalized self-radius for mC-define major clusters and subclusters. For (C-D), gray lines represent individual clusters while colored lines represent means and confidence intervals. (E) Over-splitting score for each major cluster (in green) and associated sub-clusters (in yellow). Dot size of sub-clusters represents cluster size normalized by the size of their "mother" major cluster. (F) Under-splitting score for each major cluster (in green) and associated sub-clusters (in yellow). (G-K) Fusion of snmCAT-seq transcriptome and mC profiles using the Single Cell Fusion (G), Seurat (H), Harmony (I), LIGER (J), and Scanorama (K). For each computational data fusion

method, from top to bottom the first panel shows mC and RNA modalities on the joint UMAP embedding after data fusion. The second panel shows the normalized self-radius. The third and fourth panels show the co-cluster level cell composition and clustering accuracy.

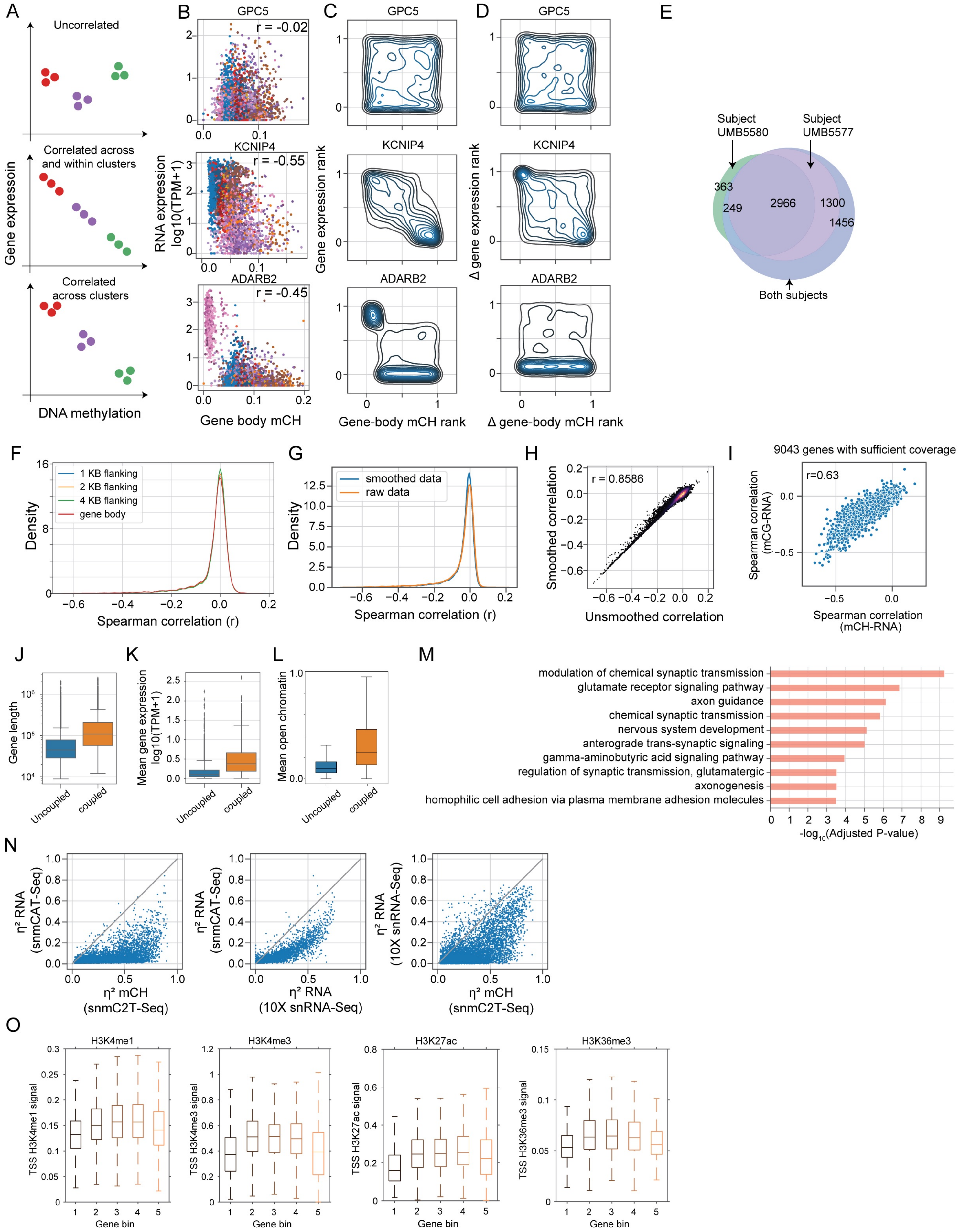
A
Uncorrelated
Correlated across and within clusters
Correlated across clusters
Gene expressoin
DNA methylation
B
GPC5
r = -0.02
KCNIP4
r = -0.55
ADARB2
r = -0.45
RNA expression log10(TPM+1)
Gene body mCH
C
Gene expression rank
Gene-body mCH rank
D
Δ gene expression rank
Δ gene-body mCH rank
E
Subject UMB5580
Subject UMB5577
363
249
2966
1300
1456
Both subjects
F
1 KB flanking
2 KB flanking
4 KB flanking
gene body
Density
Spearman correlation (r)
G
smoothed data
raw data
Density
Spearman correlation (r)
H
r = 0.8586
Smoothed correlation
Unsmoothed correlation
I
9043 genes with sufficient coverage
r=0.63
Spearman correlation (mCG-RNA)
Spearman correlation (mCH-RNA)
J
Gene length
Uncoupled
coupled
K
Mean gene expression log10(TPM+1)
L
Mean open chromatin
M
modulation of chemical synaptic transmission
glutamate receptor signaling pathway
axon guidance
chemical synaptic transmission
nervous system development
anterograde trans-synaptic signaling
gamma-aminobutyric acid signaling pathway
regulation of synaptic transmission, glutamatergic
axonogenesis
homophilic cell adhesion via plasma membrane adhesion molecules
-log10(Adjusted P-value)
N
η² RNA (snmCAT-Seq)
η² mCH (snmC2T-Seq)
η² RNA (10X snRNA-Seq)
O
H3K4me1
H3K4me3
H3K27ac
H3K36me3
TSS H3K4me1 signal
TSS H3K4me3 signal
TSS H3K27ac signal
TSS H3K36me3 signal
Gene bin

Figure S4

Figure S4. Diverse correlations between gene expression and gene body mCH, related to Figure 3. (A) Schematic diagram of 3 types of genes with different correlations between gene expression and DNA methylation. (B) Scatter plots of gene body mCH (unnormalized) and gene expression of example genes (KCNIP4, ADARB2, GPC5) across all neuronal cells. Cells are colored by major cell types defined in Figure 4. (C) Contour density plot of gene body mCH rank versus gene expression rank for 3 example genes: GPC5, KCNIP4, and ADARB2. (D) Contour density plot of delta gene body mCH rank (rank of gene body mCH - cluster mean gene body mCH) versus delta gene expression rank (rank of gene expression - cluster mean gene expression) for 3 example genes: GPC5, KCNIP4, and ADARB2. (E) Venn diagram showing a strong overlap of mCH-RNA coupled genes identified using cells from subject UMB5580, subject UMB5577, and from both subjects. (F) Distribution of Spearman correlation coefficient between gene expression and gene-level mCH quantified at gene body (red), gene body + 1 kilo-base upstream (blue), + 2 kilo-base upstream (orange) and + 4 kilo-base upstream (green). (G) Distribution of Spearman correlation coefficient between gene expression and gene-level mCH quantified at gene body with or without data smoothing before correlation analysis. (H) Scatter plot comparing the effect of data smoothing on Spearman correlation coefficients computed between gene expression and gene-level mCH. (I) Scatter plot comparing gene body mCH-RNA correlation versus gene body mCG-RNA correlation. (J-L) Boxplot of gene length (J), mean gene expression level (K) and mean open chromatin level (L) for mCH-RNA uncoupled and coupled genes. (M) Gene ontology enrichment of mCH-RNA coupled genes. (N) Scatter plots comparing the fraction of variance explained by cell type (η^2) for each gene from

different datasets or data modalities: RNA (from snmCAT-seq), mCH (from snmCAT-seq) and 10X (snRNA-Seq from 10X protocols). (O) The distribution of different histone marks at TSS over 5 gene bins grouped according to gene expression ratio of early fetal (PCW 8-9) to adult (>2 yrs).

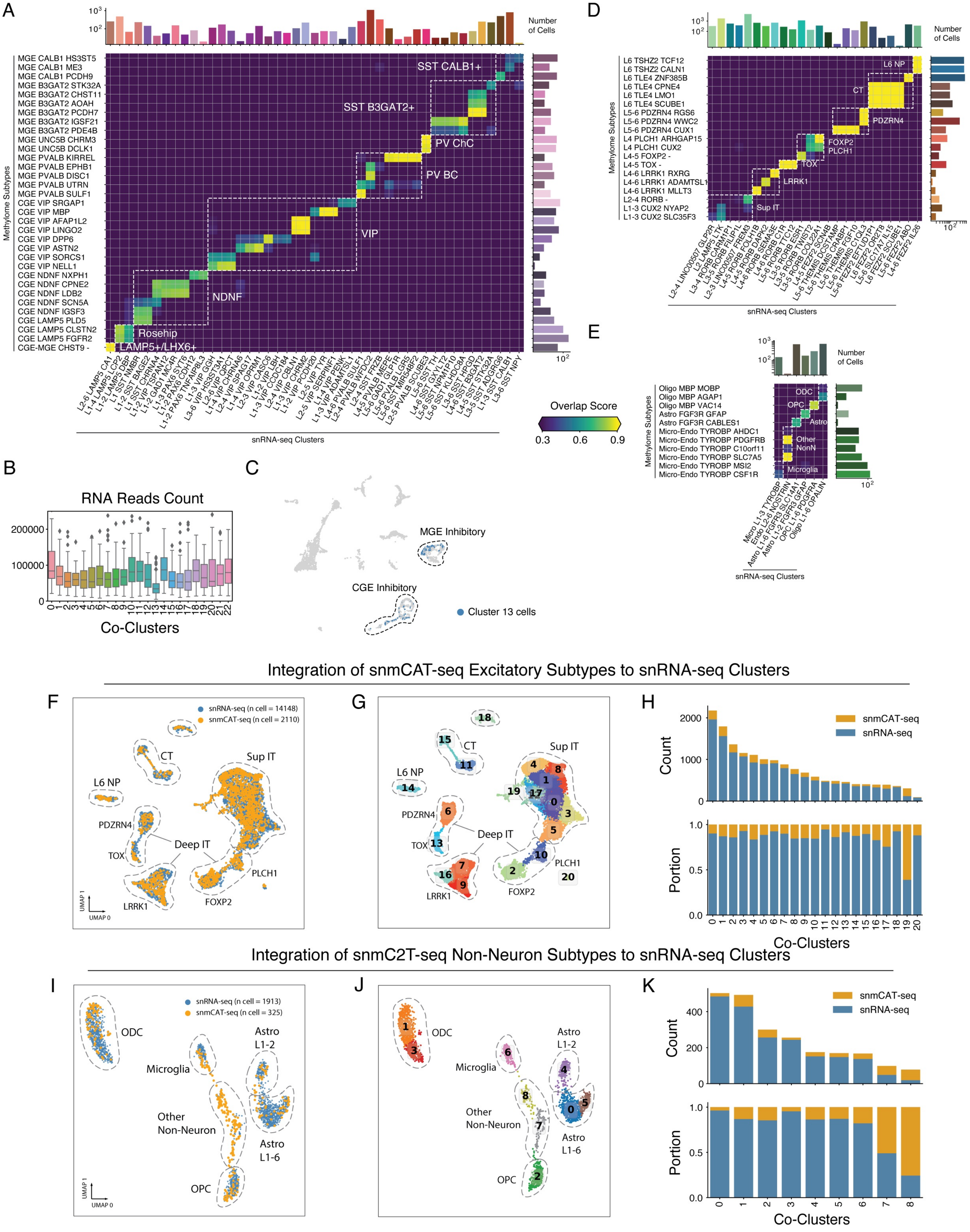
A
Number of Cells
Methylome Subtypes
MGE CALB1 HS3ST5
MGE CALB1 ME3
MGE CALB1 PCDH9
MGE B3GAT2 STK32A
MGE B3GAT2 CHST11
MGE B3GAT2 AOAH
MGE B3GAT2 PCDH7
MGE B3GAT2 IGSF21
MGE B3GAT2 PDE4B
MGE UNC5B CHRM3
MGE UNC5B DCLK1
MGE PVALB KIRREL
MGE PVALB EPHB1
MGE PVALB DISC1
MGE PVALB UTRN
MGE PVALB SULF1
CGE VIP SRGAP1
CGE VIP MBP
CGE VIP AFAP1L2
CGE VIP LINGO2
CGE VIP DPP6
CGE VIP ASTN2
CGE VIP SORCS1
CGE VIP NELL1
CGE NDNF NXPH1
CGE NDNF CPNE2
CGE NDNF LDB2
CGE NDNF SCN5A
CGE NDNF IGSF3
CGE LAMP5 PLD5
CGE LAMP5 CLSTN2
CGE LAMP5 FGFR2
CGE-MGE CHST9 -
SST CALB1+
SST B3GAT2+
PV ChC
PV BC
VIP
NDNF
Rosehip
LAMP5+/LHX6+
snRNA-seq Clusters
Overlap Score
0.3
0.6
0.9
B
RNA Reads Count
200000
100000
0
Co-Clusters
C
MGE Inhibitory
CGE Inhibitory
Cluster 13 cells
D
Number of Cells
Methylome Subtypes
L6 TSHZ2 TCF12
L6 TSHZ2 CALN1
L6 TLE4 ZNF385B
L6 TLE4 CPNE4
L6 TLE4 LMO1
L6 TLE4 SCUBE1
L5-6 PDZRN4 RGS6
L5-6 PDZRN4 WWC2
L5-6 PDZRN4 CUX1
L4 PLCH1 ARHGAP15
L4 PLCH1 CUX2
L4-5 FOXP2 -
L4-5 TOX -
L4-6 LRRK1 RXRG
L4-6 LRRK1 ADAMTSL1
L4-6 LRRK1 MLLT3
L2-4 RORB -
L1-3 CUX2 NYAP2
L1-3 CUX2 SLC35F3
L6 NP
CT
PDZRN4
FOXP2
PLCH1
TOX
LRRK1
Sup IT
snRNA-seq Clusters
E
Number of Cells
Methylome Subtypes
Oligo MBP MOBP
Oligo MBP AGAP1
Oligo MBP VAC14
Astro FGF3R GFAP
Astro FGF3R CABLES1
Micro-Endo TYROBP AHDC1
Micro-Endo TYROBP PDGFRB
Micro-Endo TYROBP C10orf11
Micro-Endo TYROBP SLC7A5
Micro-Endo TYROBP MSI2
Micro-Endo TYROBP CSF1R
ODC
OPC
Astro
Other NonN
Microglia
snRNA-seq Clusters
Integration of snmCAT-seq Excitatory Subtypes to snRNA-seq Clusters
F
snRNA-seq (n cell = 14148)
snmCAT-seq (n cell = 2110)
CT
Sup IT
L6 NP
PDZRN4
Deep IT
TOX
PLCH1
LRRK1
FOXP2
UMAP 1
UMAP 0
G
H
snmCAT-seq
snRNA-seq
Count
Portion
Co-Clusters
Integration of snmC2T-seq Non-Neuron Subtypes to snRNA-seq Clusters
I
snRNA-seq (n cell = 1913)
snmCAT-seq (n cell = 325)
ODC
Astro L1-2
Microglia
Other Non-Neuron
Astro L1-6
OPC
J
K
snmCAT-seq
snRNA-seq
Count
Portion
Co-Clusters

Figure S5

Figure S5. snmCAT-seq recapitulates transcriptome and methylome signatures of neuronal subtypes, related to Figure 5. (A) Confusion matrix showing the overlap scores between inhibitory subtypes identified by ensemble methylome analysis and snRNA-seq. Known inhibitory cell type groups are annotated by boxes. The upper bar plot indicates the snRNA-seq cell counts per cluster; the right bar plot indicates the snmCAT-seq cell counts per cluster. (B) Cluster 13 in Figure 5A-C includes snmCAT-seq transcriptome profiles with lower RNA read counts. (C) snmCAT-seq methylome profiles of single nuclei in cluster 13 (colored in blue) can be readily integrated with other inhibitory cells. (D-E) Confusion matrix showing the overlap scores between methylome ensemble subtypes and the snRNA-seq clusters for excitatory neuron clusters (D) and non-neuron clusters (E). Known cell type groups are annotated by boxes. The upper bar plot annotates the snRNA-seq cell counts per cluster, the right bar plot annotates the snmCAT-seq cell counts per cluster. (F-G) UMAP embedding of all excitatory neurons profiled by snmCAT-seq and snRNA-seq after MNN-based integration, colored by technology (F) and joint clusters (G). Known cluster groups are also circled and annotated on UMAP. (H) The composition of cells profiled by snmCAT-seq and snATAC-seq in excitatory neuron joint clusters. The upper and lower bar plots show the counts and portion of cells profiled by the two technologies in each joint cluster, respectively. (I-J) UMAP embedding of all non-neuronal cells from snmCAT-seq and snRNA-seq after integration, colored by technology (I) and joint clusters (J). (K) The composition of cells profiled by snmCAT-seq and snATAC-seq in non-neuronal cell joint clusters.

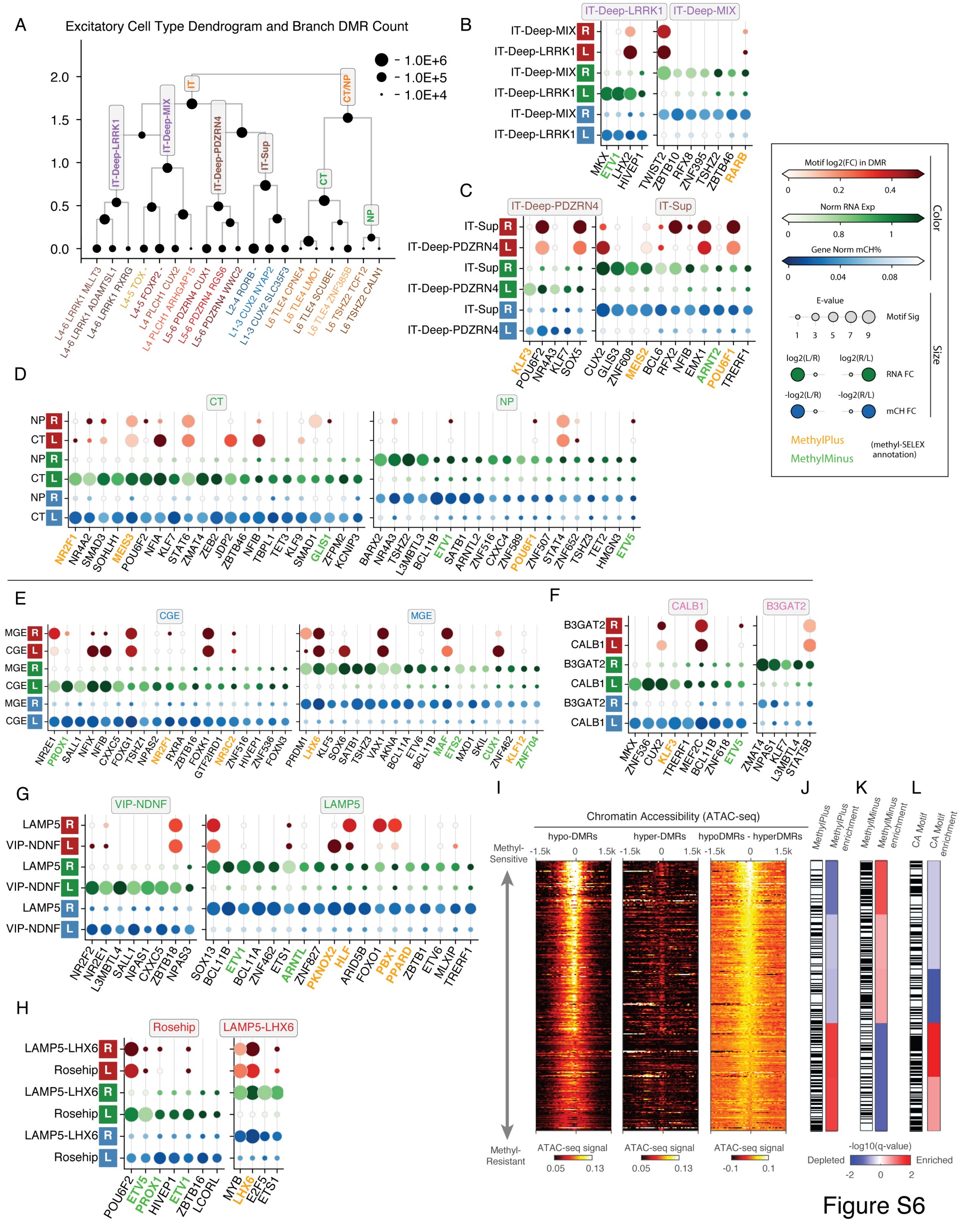
A
Excitatory Cell Type Dendrogram and Branch DMR Count
1.0E+6
1.0E+5
1.0E+4
IT-Deep-LRRK1
IT-Deep-MIX
IT
IT-Deep-PDZRN4
IT-Sup
CT/NP
CT
NP
B
C
D
E
F
G
H
I
J
K
L
Motif log2(FC) in DMR
Norm RNA Exp
Gene Norm mCH%
Color
E-value
Motif Sig
Size
RNA FC
mCH FC
MethylPlus
MethylMinus
(methyl-SELEX annotation)
Chromatin Accessibility (ATAC-seq)
hypo-DMRs
hyper-DMRs
hypoDMRs - hyperDMRs
Methyl-Sensitive
Methyl-Resistant
ATAC-seq signal
-log10(q-value)
Depleted
Enriched

Figure S6

Figure S6. TF binding motif enrichment across the human cortical neuronal hierarchy, related to Figure 6. (A) Excitatory neuron subtype dendrogram. The node size represents the number of DMRs detected between the left and right branches. (B-D) Dot plots view for TFs showing lineage-specific motif enrichment, expression and gene body mCH between lineages: intratelencephalic (IT)-Deep-LRRK1 vs IT-Deep-MIX (B); IT-Deep-PDZRN4 vs IT-Sup (C); Cortico-Thalamic projection (CT) vs. layer 5/6 near-projecting neurons (NP) (D). Colors for every two rows from top to top: lineage mean gene body mCH level, lineage mean expression log(1 + CPM), TF motif enrichment log2(fold change). Sizes for every two rows from bottom to top: relative fold change of mCH level from this branch to the other, relative fold change of expression level, E-value of the motif enrichment test. Colors for the motif names: TF motif methylation preference annotated by methyl-SELEX experiment (Yin et al., 2017), orange indicate MethylPlus, green indicate MethylMinus. (E-H) Dot plot view for TFs showing lineage-specific motif enrichment, expression and gene body mCH between CGE vs. MGE (E), CALB1 vs. B3GAT2 (F), VIP/NDNF vs. LAMP5 (G) and Rosehip vs LAMP5-LHX6 (H). (**I**) The binding of TFs to hypermethylated regions validated by chromatin accessibility measurement using the snATAC-seq profile. (**J-L**) Enrichment or depletion of MethylPlus TFs (J), MethyMinus TFs (K) and TFs whose binding motif containing CA dinucleotides (L).

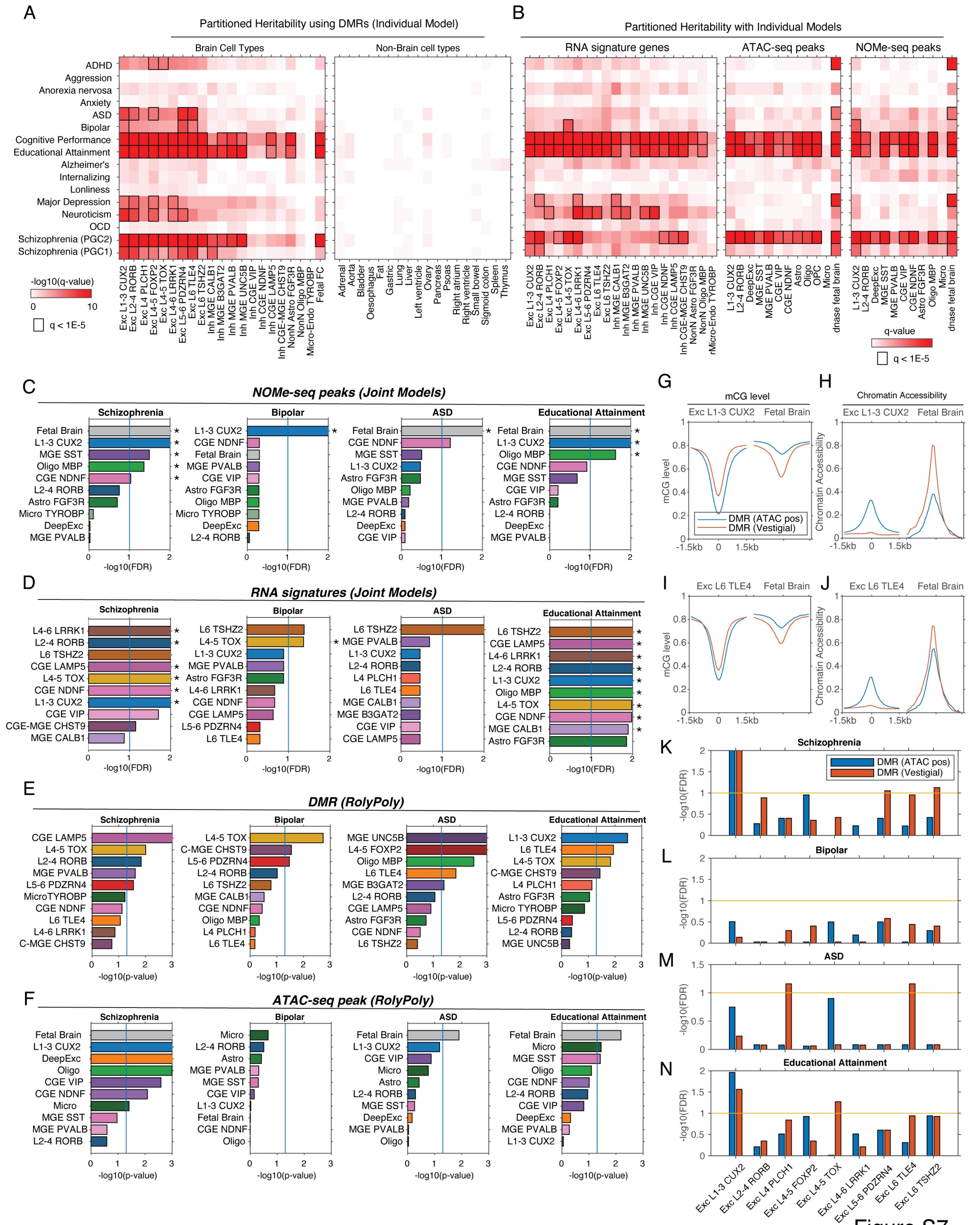

A
Partitioned Heritability using DMRs (Individual Model)
Brain Cell Types
Non-Brain cell types
B
Partitioned Heritability with Individual Models
RNA signature genes
ATAC-seq peaks
NOMe-seq peaks
C
NOMe-seq peaks (Joint Models)
D
RNA signatures (Joint Models)
E
DMR (RolyPoly)
F
ATAC-seq peak (RolyPoly)
G
mCG level
H
Chromatin Accessibility
I
J
K
Schizophrenia
L
Bipolar
M
ASD
N
Educational Attainment
DMR (ATAC pos)
DMR (Vestigial)
-log10(q-value)
q < 1E-5
-log10(FDR)
-log10(p-value)

Figure S7

Figure S7. Prediction of causal cell types for neuropsychiatric traits using partitioned heritability analysis, related to Figure 7. (A) Partitioned heritability analysis individually comparing (individual models) adult brain cell-type-specific DMRs, fetal cortex lowly methylated regions, and non-brain tissues to the baseline (B) Partitioned heritability analysis (individual models) using adult brain RNA signature genes, ATAC-seq peaks, and NOMe-seq peaks. (C-D) Multiple regression partitioned heritability analysis using NOMe-seq peaks (C) and gene expression signatures (D). (E-F) Prioritization of neuropsychiatric traits-associated brain cell types using RolyPoly. (G-J) CG methylation and chromatin accessibility profiles at adult brain regulatory elements [DMR (ATAC-pos)] and vestigial enhancers. (K-N) Multiple regression partitioned heritability analysis comparing adult regulatory elements and [DMR (ATAC-pos)] and vestigial enhancers.
